# ARACHNE: A neural-neuroglial network builder with remotely controlled parallel computing

**DOI:** 10.1371/journal.pcbi.1005467

**Published:** 2017-03-31

**Authors:** Sergey G. Aleksin, Kaiyu Zheng, Dmitri A. Rusakov, Leonid P. Savtchenko

**Affiliations:** 1 AMC Bridge LLC, Waltham MA, United States of America and Dnipro, Ukraine; 2 UCL Institute of Neurology, University College London, London WC1N 3BG, United Kingdom; 3 Institute of Neuroscience, University of Nizhny Novgorod, Nizhny Novgorod, Russia; UCSD, UNITED STATES

## Abstract

Creating and running realistic models of neural networks has hitherto been a task for computing professionals rather than experimental neuroscientists. This is mainly because such networks usually engage substantial computational resources, the handling of which requires specific programing skills. Here we put forward a newly developed simulation environment ARACHNE: it enables an investigator to build and explore cellular networks of arbitrary biophysical and architectural complexity using the logic of NEURON and a simple interface on a local computer or a mobile device. The interface can control, through the internet, an optimized computational kernel installed on a remote computer cluster. ARACHNE can combine neuronal (wired) and astroglial (extracellular volume-transmission driven) network types and adopt realistic cell models from the NEURON library. The program and documentation (current version) are available at GitHub repository **https://github.com/LeonidSavtchenko/Arachne** under the MIT License (MIT).

This is a *PLOS Computational Biology* Software paper.

## Introduction

Neural network simulation remains an important and powerful tool to understand principles that underpin the functional organisation and multi-faceted activities of the human brain. There have been at least several dozen successfully implemented large-scale scale network simulators enabling the exploration of multicellular assemblies at various levels of organisational and functional detail. These include Topographica [1], Nest [2], Brian [3], ANNarchy [4], NEURON [5], Genesis [6], Auryn [7], Nengo [8], PyNN’s [9], NeuroManager [10]. Among such tools, it appears that Genesis [6] (new version in the development stage) and NEURON [5] have been most frequently employed by a wide neuroscience community. NEURON in particular provides a tool to create some highly realistic, experimentally tested cell models and their networks, with parallel computation add-ons. These features have been successfully adopted by the Blue Brain project [11], the most ambitious attempt to recreate mammalian brain functions *in silico*. However, the degree of virtual reality that would satisfy a brain scientist (such as in Blue Brain) is a matter of having state-of-the-art supercomputers, the corresponding programming expertise, and the resources and skills for maintenance. These are not routinely available to experimental neuroscientists.

Among such modelling tools, NeuroManager [10] represents a simulation management software interfacing with other tools such as NEURON [12]; this normally requires professional knowledge of Python [3, 9], C++ [7] or Java [8]. Network modellers such as Brian, NEST, NEURON, GENESIS, Nengo, or Auryn focus on parallel simulations on shared memory systems (multi-core or multi-processor) or distributed systems (clusters) using either OpenMP (open multi-processing) or MPI (Message Passing Interface). Some of the more purpose-tuned neural simulators including GeNN1, MVAPICH [13], NeMo [14], and CARLsim [15] provide support for simulations on a single or multiple GPU architectures. Again, these diverse systems adapt the technical programming solutions specific to the task under study, which, in many cases, requires a specific programming language, often with a high degree of semantic and linguistic development. This in turn demands programming skills and experience. Furthermore, while reflecting the enormous complexity and diversity of brain circuits the narrow specialisation of the modelling paradigm can significantly narrow the users' pool.

A somewhat different approach to neural network modelling refers to a brain machine that incorporates standard logic devices and mathematical operators mimicked by the integrate-and-fire cell circuits adapted to produce a desired response function or operation, be it a filter, integrator, attractor, or else [16]. This ‘top-down’ modelling method is capable of successfully reproducing some key recognition and memory functions, from perceptive input to motor output [16]. However, such models create and connect elements of artificial neural networks in order to perform a desired behaviour rather than reproducing real-world brain circuits with an aim to understand their function. Similarly, neural network algorithms underpinning industrial robots do not generally aim at understanding how the brain circuitry works. The latter nonetheless is what neuroscientists strive to achieve. In contrast, network models implementing synaptic plasticity rules [17, 18] could provide conceptual insights into the principles of synaptic circuit functioning.

Another principal complexity in the field has recently transpired. All well-established neural network simulators deal with excitable nerve cells communicating via individual cell-cell connections representing synaptic inputs. However, it has emerged that the other common type of brain cells—glia, and especially astroglia—can directly influence brain circuits [19–21]. Most astroglia are non-excitable cells that handle physiological signals through intracellular Ca^2+^ waves [22, 23], occupy non-overlapping tissue domains (each hosting many thousands of synapses on different neurons) [24], and release a variety of signalling molecules into the extracellular space [25, 26]. Thus, astrocytes operate a diffuse, or 'volume-transmitted', type of extracellular signalling, which is qualitatively different from the 'wired' transmission underpinning classical neural networks [27]. When incorporated into the neural network, this volume-transmitted signals prompts neural network state transitions [28] which are yet to be understood. To our knowledge, there have been no systematic attempts to incorporate this (physiologically essential) type of cell-cell communication in the neural network software.

Here, in developing the modelling tool ARACHNE our aim was therefore to enable experimental neuroscientists to build, run and explore complex, realistic cellular networks incorporating neurons (wired connections) and astroglia (extracellular diffuse signalling), with little computational expertise and little computational resource available on site. In some respects, ARACHNE follows the logic of "neuroConstruct" [29], a neuroscientist-friendly shell (add-on) helping to create 3D networks of realistic cells using NEURON or GENESIS, but with an advantage of having its own computational kernel. We thus sought to build a simple interface for model creation and running combined with a powerful simulation tool adapted to extensive resources for parallel computing. To enable realistic cell representations, ARACHNE was to provide direct upload of membrane biophysical mechanisms from the NEURON library. This option allows an inexperienced user to take advantage of the NEURON database and the tools of ARACHNE in setting up a realistic cellular network. Finally, the interface was to enable full computational control of network simulations from a mobile device.

## Design and implementation

The host application is running under Windows. It can be launched from the same machine or a remote mobile device (Android or iOS). The HPC kernel (C++) operates under either Linux or Windows.

Currently ARACHNE provides four configuration types:

(GUI) Windows ↔ (kernel) Windows,

(GUI) Windows ↔ (kernel) Linux,

(CLI*) Android ↔ (No GUI) Windows ↔ (kernel) Windows,

(CLI*) Android ↔ (No GUI) Windows ↔ (kernel) Linux,

where *CLI—Command Line Interface.

ARACHNE also supports a silent mode in which the GUI is not used, and all the input parameters are transferred to the host entry point in a “struct” of MATLAB.

**Fig 1:**
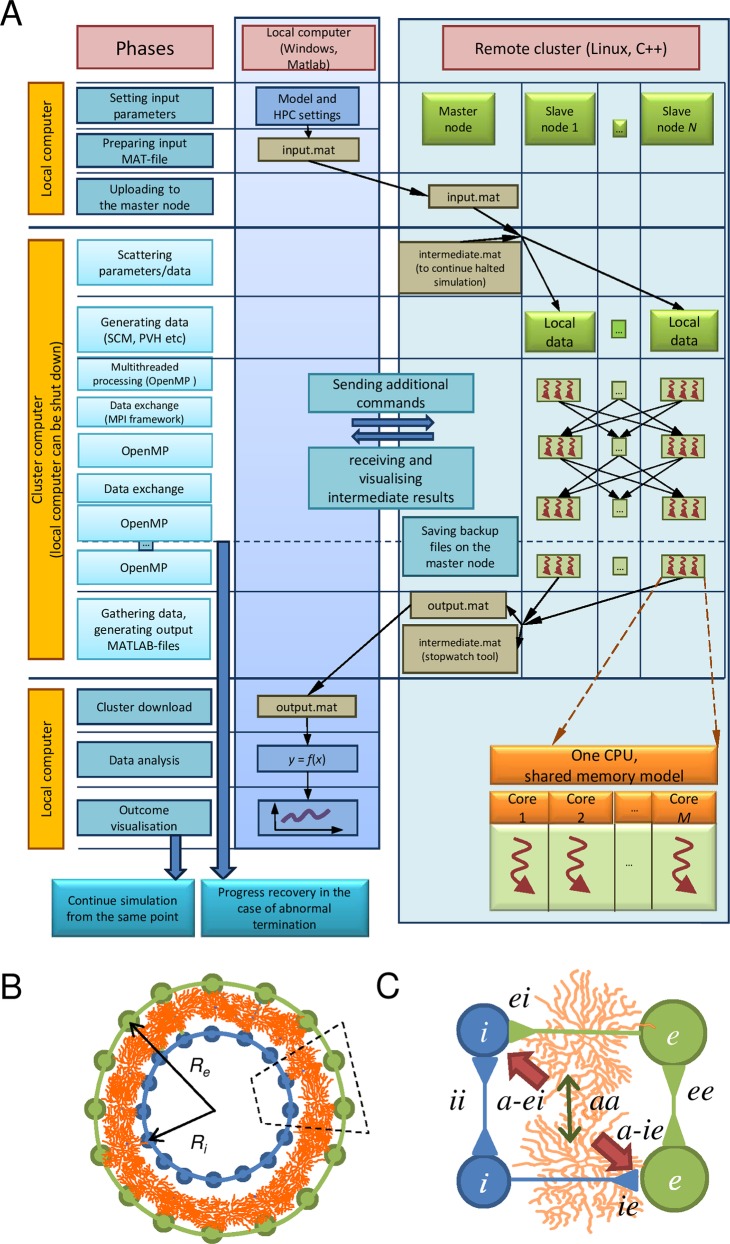
Structure of ARACHNE and simulated network types. (A) General diagram of the ARACHNE simulator. In brief, local computer generates the model and the HPC configuration as input.mat file, which is sent to the remote computer with master and slaves N clusters. Each slave computer has M processors. After the parallel computation has run the results recorded in output.mat file are sent back to the local computer. (B) Diagram depicting three key network types: principal neurons (*e-neurons)*, interneurons (*i-neurons*) and astrocytes (*a-cell*); *R*_*e*_ and *R*_*i*_, the network size (radius), respectively. (C) A network fragment depicted by dotted area in (B); different types of cell-cell signalling types are indicated including an *aa* connection reflecting (mostly) astrocyte gap junctions.

The Linux operating kernel was tested on a remote, ad hoc-built 12-node cluster [30], which we have previously used and optimized for Monte Carlo simulations [28, 31–35]. The kernel performs numerical integration of a massive system of ODEs describing the biophysical states and the topology of cell networks (Fig 1).

The GUI enables the user (a) to create a network model, (b) to control simulations, and (c) to keep all the network configurations, the input data and the results on a local drive using only the GUI. At this stage, the design of neuronal networks does not require programming skills. The user generates an input file (input.mat) via the GUI, including parameters of the network and settings for computation (S1 File, Supplementary Material). Once the input.mat has been sent to the cluster to execute computations, the user can either employ the interface-cluster link to monitor the computation process or disconnect it altogether. The input.mat file is small enough to be sent through a limited bandwidth connection such as 3G networks. Once the simulations have been completed, the results (output.mat) are sent to the interface computer and stored on the cluster for further analyses. The system architecture has sufficient provisions for a mobile applet that would enable general public sourcing and migration to cloud to help building realistic networks. The requirement to the cluster is the presence of either Linux or Windows and the availability of “Open MPI C++ wrapper compiler” and MATLAB for simple compilation.

Various hosts may interact with the same cluster sequentially. In brief, communication between the local computer and the remote cluster uses the SSH network protocol (Fig 1). We use PuTTY applications to execute commands on a remote computer and to exchange files in between. In particular, we use Plink (a command-line interface to the PuTTY back ends) and PSCP (a SCP client, i.e. command-line secure file copy). In the current version of software, we do not provide an interface to TORQUE, which enables control over batch jobs and distributed computing resources. ARACHNE currently assumes no simultaneous access of multiple users to a single cluster but allows individual access in a queue. Several users can monitor one simulation (i.e. visualise intermediate results on different local machines), but cannot run more than one simulation on the cluster at the same time. The software fully manages the communication between local MATLAB host and remote C++ worker, no user action required. The host automatically packages the data, uploads it to the cluster and tells the remote cluster to begin execution. This occurs once the user has set up simulation parameters in the GUI and clicked the “OK” button. ARACHNE is thus organised in such a way that it enables an untrained user to create a large network and to operate computation on and communication with the cluster. The GUI has a special option called HPC where the user can easily change the parameters of cluster computation.

The kernel solves the set of differential equations related to the membrane potential of neurons Eq (1) and intracellular calcium dynamics Eq (2) for astrocytes. When a new cellular mechanism is added to the kernel, the corresponding C++ code has to be appended and recompiled as required.

In order to expand the modelling capabilities, the ARACHNE GUI can incorporate membrane mechanisms from the NEURON database (mod-files). The incorporation of new mechanisms requires recompilation of C++ code of the HPC kernel located on the cluster.

To reduce computation time for a newly designed neural network, we have envisaged two distinct operating modes for the kernel. Mode ***I*** is designed for the optimization of any newly configured network. The key goal here is to determine how best to parallelise the network, and how much memory should be allocated in order to increase the speed and the accuracy of calculations. Mode ***II*** applies to the exploratory computations with a fixed cluster configuration. Here, users may also employ various nodes of the cluster at their discretion. This mode is highly suitable for exploring the parameter space for a given neural network architecture.

### The biophysical basis: A case study

ARACHNE includes basic pre-set parameters reproducing local cellular networks in hippocampal area CA1, a well-explored subject of neural modelling [36, 37]. Unlike previous models consisting of inter-connected neurons only, ours also incorporated astroglia (enabling a diffuse, volume-transmission extracellular signalling mechanism acting at subgroups of neighbouring neurons). Thus, each of the three cellular networks (Fig 1B)—***i-****neurons* (inhibitory neurons), ***e-****neurons* (excitatory neurons), and ***a-****cells* (electrically non-excitable astrocytes)—was equipped with a specific set of biophysical, architectural and topological features. Biophysical parameters describe known physiological mechanisms present in each cell type, such as ion channels and pumps [38–40], ion diffusion, receptor currents, etc (S2 File).

The basic dynamic variables represent the main cellular communication mechanisms in the network: these are membrane potential *V* for neurons (1) and the intracellular calcium concentration for astrocytes. The dynamics of *V* for both types of neurons is described by a set of equations with the Hodgkin–Huxley formalism:

$$C_{m}\frac{dV_{}}{dt}=\sum I_{n}+I_{syn}$$ (1)

where *C*_*m*_ is a membrane capacitance and $\sum I_{n}$ is a sum of transmembrane currents (S2 File) and *I*_*syn*_ is synaptic current from nearby neurons with plasticity mechanisms allowing self-organization of network connections with the bottom-up approach similar to that described earlier [17].

### Astrocyte network

Astrocytes, electrically non-excitable cells, can modify release probability of nearby synapses in tissue volume [41], likely by releasing signalling molecules ('gliotransmitters') in a Ca^2+^ dependent manner [42]. In turn, neurotransmitters released by neurons influence Ca^2+^ dynamics in astroglia [43]. The basic feedback between neurons and astrocytes has thus been incorporated in ARACHNE, in which astrocytes occurring next to an *e*-cell alter adjacent synapses (Fig 1C) [44]. For the sake of simplicity, the relationship between astrocyte calcium concentration ***Ca*** and neurotransmitter release probability at affected synapses, *p*, has been described with the simple formulism based on earlier suggestions [45] (S2 File); this relationship could be modified in accord with experimental data.

The dynamics of astrocyte calcium follows the equation

$$\frac{dCa}{dt}=-\sum_{n}J_{n}$$ (2)

where *J*_*n*_ are intracellular Ca fluxes [45]. The basic interaction between astroglial Ca^2+^ and synaptic circuitry modulation, which is implemented here mainly for illustration purposes, can be modified in accord with the emerging experimental data on astroglia-neuron communication.

## Results

### Optimizing the model configuration

**Fig 2:**
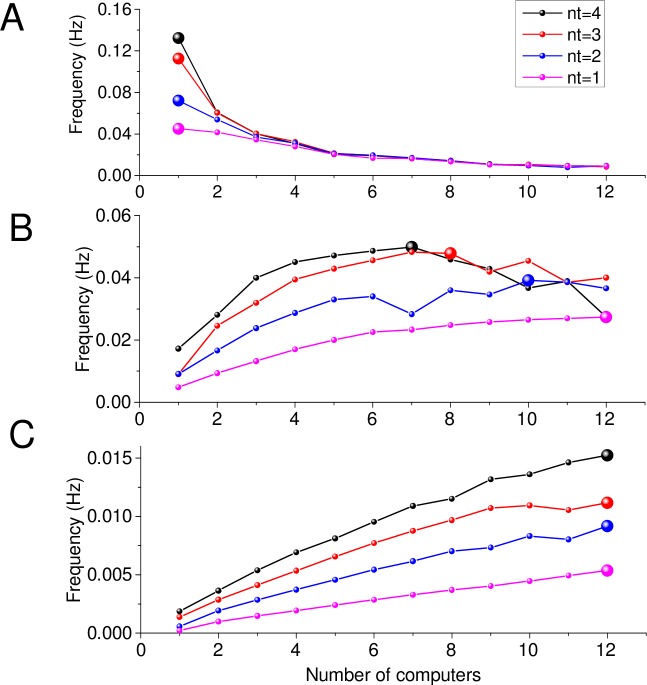
Tests to determine optimal performance. (A) Performance indicator versus number of computers: examples for small *i*- and *e*- networks (100 cells). Ordinate, frequency (1 / runtime). Large balls, the optimal number of computers; n_t_, the number of cores per processor. Scalability tests were performed on a cluster of 12 computers, each with 4-core processors. (B-C) Similar tests as in (A) for a medium (B, 1000 cells) and larger (C, 4000 cells) network. Other notations as in (A).

A newly created network model designed for a multiprocessor cluster with a parallel algorithm will require an initial optimisation step. The outcome of such optimisation is the number of cluster workers that provides the fastest possible computation. To explore and illustrate this optimisation strategy, we have tested three identical neural networks with the unchanged, 'basic set' of parameters (S2 File) but with different numbers of neurons (100, 1000 and 4000). The examples of optimization (Fig 2) illustrate a search for the number of cluster workers that provides the highest frequency of execution, for a given network (Fig 2).

The optimisation tests reveal that a computer with a large number of processor cores appears computationally optimal for relatively small networks (Fig 2A). As the network size increases (Fig 2B and 2C) the optimal number of computers tends to rise keeping the optimal computation. ARACHNE enables the user to specify the maximum size of the network for a given set of parameters and the size of the computer cluster. Parallelisation is critical for improving computational performance. At first glance, calculations are quicker and more accurate with larger computer numbers. In fact, our tests indicate that this is not always the case, in line with the Amdahl's law [46].

### Exploration example: Network size versus network dynamics

**Fig 3:**
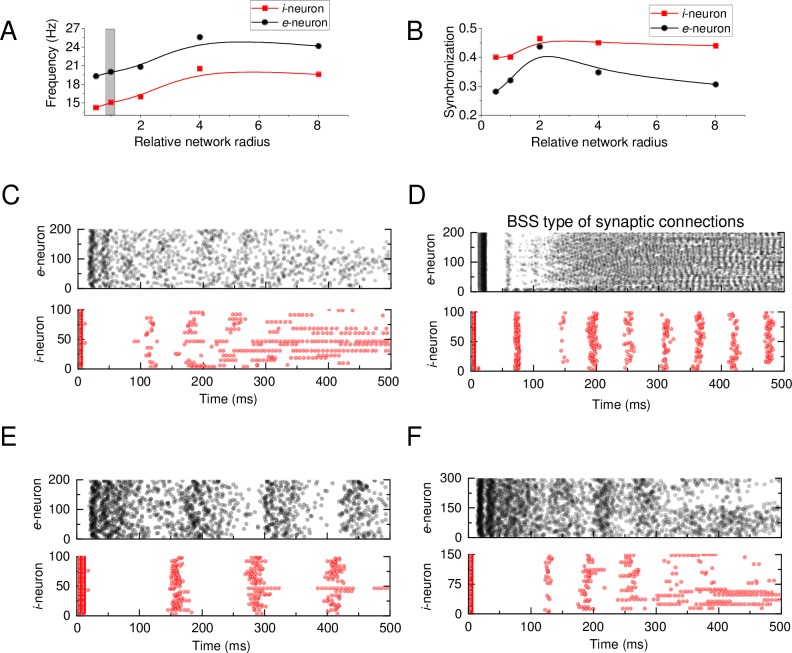
Network organisation versus rhythm genesis and synchronisation. (A-B) Frequency (A) and synchronization (B) indicators *versus* the relative radius of *e*-network and *i*-network (relative to their 'reference' radiii 250 μm and 200 μm, respectively). (C) Spiking raster plots of the ‘basic-set’ (S2 File Biophysical model) networks, including the BSD type synaptic weight distribution. (D) Spiking raster plots for ‘basic-set’ (S2 File) networks (ratio = 1), but with the BSS type synaptic distribution. (E) Spiking raster plots of ‘basic-set’ networks, but with the network radii increased two-fold (corresponds to the abscissa value of 2 in A-B). (F) Spiking raster plots for ‘basic-set’ networks, but with the total numbers of both *e*-neurons and *i*-neurons increased 1.5-fold.

The network size and the distribution of synapses could strongly affect the network activity, even when all other settings remain unchanged. To explore this relationship we focused on the network main spiking frequency (Fig 3A) and synchronization (Fig 3B) as readout parameters [35]. The network 'main' frequency was calculated as the average frequency $F=\frac{1}{N}\sum_{i=1}^{N}f_{i}$ of all neurons *N* with an individual frequency *f*_*i*_ during the computation time *T*. Synchronization was calculated as a correlation between spike timing for all neurons in the network during time *T*. The raster plots were therefore obtained for four characteristic cases: (i) the base network configuration (Fig 3C), (ii) doubled size (Fig 3E), (iii) increased numbers of neurons (Fig 3F), and (iv) *BSS* type of synaptic distribution (Fig 3D). Among other things, these results clearly indicate that the network size alone could have a significant impact on the network dynamics.

In the brain, the synaptic strength appears to depend on the distance between cortical neurons [47]: to recapitulate this observation, the model provides two complementary types of connectivity. The first type, termed bell-shaped strength (*BSS*) model, incorporates a Gaussian distribution of synaptic weights ***w*** (centred at the 'presynaptic' cell, standard deviation σ) with the uniform connection density between the nearest 50% of all network neurons (S2A Fig). The second type, a 'bell-shaped' connection density (*BSD*) model, incorporates uniform distribution of synaptic weights ***w*** but a Gaussian distribution of cell-cell connection density (S2B Fig), with the number of connections decreasing with distance from the 'presynaptic' cell.

### Exploration example: Network memorisation and recall

**Fig 4:**
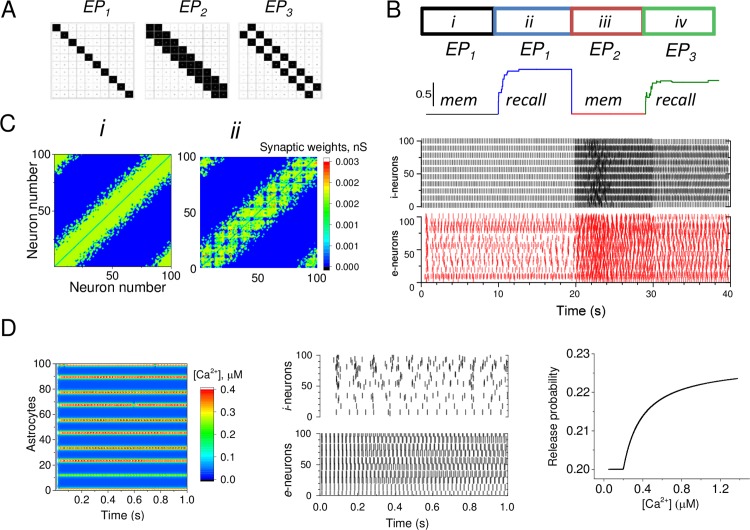
Exploring network memorisation, recall, and the effects of astroglial signalling. (A) External input patterns (EPs) used in simulations, as indicated. (B) *Top***,** four successive network stages (*i-iv*) of memorisation and recall, and the corresponding EPs, as indicated. *Middle trace*, dynamics of the recall quality (colours depict network stages). *Bottom*, spiking raster plots depicting the overall dynamics of *e-* and *i*-networks corresponding to the four stages as above. (C) Example of the *ee* synaptic connections matrixes corresponding to the end of stages *i* and *ii*, as shown in (B). In simulations shown in (A-C) astrocytes are switched off. (D) *Left*, Color-coded time map of astrocyte calcium dynamics during stage *i* shown in (B). *Middle*. Spiking raster plot of *e*- and *i*-networks that corresponds to the astrocyte calcium dynamics shown on the left. *Right*. The hypothetical relationship between the *ei*-connection synaptic released probability and the astrocyte calcium concentration.

The network memory formation is reflected in a change in the connectivity matrix (Fig 4C) resulting from an external input (Fig 4A). In this respect, ARACHNE includes two scenarios, one of memorisation (Fig 4B, *i* and *iii*) and the one of recall (Fig 4B, *ii* and *iv*). The modelled networks can in fact incessantly memorise and recall: the sequence of such events is shown in Fig 4.

The first scenario (Fig 4B, stage *i* and *ii*) deals with the memorisation of the external pattern (*EP)* only. When *EP*_*1*_ (Fig 4A) is applied to *e*-cells, both networks begin to generate action potentials (APs). This prompts activity-dependent plastic changes in synaptic connections depending on the correlations between the APs of presynaptic and postsynaptic neurons. After a memorisation period, the synaptic weighs are stabilised, thus forming a new memory matrix, such as the example *ee-*matrices that formed in response to *EP*_*1*_ and *EP*_2_ (Fig 4C).

The second scenario (Fig 4B, stage *ii*) was designed to simulate a recall process. In this mode, the mechanism of synaptic modification was switched off. When either *EP*_1_ or *EP*_3_ activates the networks the neurons start to generate a pattern of APs, with the matrix of synaptic weights remaining unchanged. At this stage, the model calculates the difference between two patterns of APs (Fig 4B, *ii* and *iv;* and *i* and *iii*, correspondingly) of the network dynamics to determine the recall quality C (Fig 4B). The initial pattern of APs is formed by the *EP* during the first stage of the network configuration, and the another pattern occurs in response to a new *EP*.

These examples reveal the following. When the memorised synaptic matrix is associated with the *EP*_*1*_ (Fig 4C, stages *i* and *ii*) which is used for the memory formation, the quality of recall of the same *EP*_1_ is perfect, *C =* 1 (Fig 4B, stage *ii*). In case of *EP*_*2*_ is used during memorisation, the quality of recall is relatively poor, *C = 0*.*6*, when *EP*_3_ is used for the association (Fig 4B stage *iii*).

When the astrocyte calcium dynamics (Fig 4D, *left*) is on and the functional relationship between the astrocyte Ca^2+^ concentration and the e-cells synapses is active (Fig 4D, *right*), astrocytes begin to modify release probability of excitatory synapses (Fig 4D, *middle*). The correlation between the astrocyte calcium dynamics and the neuronal dynamics is increased and synchronized (Fig 4D, left and middle spiking raster plots). This mode of ARACHNE enables exploration of the interaction between astrocytes and neurons networks, including the role of astroglia in memory formation.

## Concluding remarks

Our aim here was to develop a neuroscientist-friendly simulation tool that would enable the design and exploration of realistic brain networks of arbitrary complexity incorporating neurons and astroglia. The flexibility and ease of use by experimental neuroscientists was among the main goals in creating ARACHNE. An important distinction of the design is the physical separation of the model management and model computations. A standard low-cost host computing device can therefore be used for the model administration including the network synthesis, the formation of input and output files, and of visual presentation of the results. Once the network configuration has been prepared, it can be uploaded onto a remote cluster. The user remains within its familiar host computing environment throughout the computations and is free to break the link to the remote computer during computations. One of the key objectives was therefore to create computational algorithms, including optimal parallelisation, which would run equally efficiently for the models of varied complexity without having a complex model interface or without engaging in any architectural programming adjustments. Anticipating a high demand for computational power, we thus separated the programming (as well as physical) environment of model management from that of model computation.

Another key feature of ARACHNE is the ability for multiple users to connect to the remote cluster from a variety of computing devices, including mobile devices, using a conceptually simple user-interface. Different participants can thus share the same low-level kernels for their own calculations, store the result locally or remotely in different files, and continue their computations from any point of the previous run. This design significantly enhances the flexibility for users to manipulate the data according to the needs of their calculations.

ARACHNE appears to be one of the first modelling tools recognising an important role of astroglia in modifying the signal transfer across synaptic circuits of the brain. Much unlike the point-to-point, 'wired’ communication mode operated by synaptic circuitries, extracellular molecular signals generated by astrocytes are transmitted diffusily through the local tissue volume thus engaging multiple synapses on multiple neighbouring cells [48]. To date, only a few attempts have been made to introduce this type of volume-transmitted, astroglia-type signalling to the computational models of classical, wired neural networks [28].

An important trait of the ARACHNE is that it provides the ability of the neuron-astroglia networks explorations using the host GUI only, without changing the computational kernel located on the remote cluster. Thus, when the size and the topology of the network changes the system could, in principle automatically, optimise the entire computational process.

Parallel remote computations are emerging as an important direction for the computational exploration of complex biological systems. To account for this, ever advancing scientific quest, the present builder provides a flexible functionality to the user. We thus emphasise that ARACHNE as described here is not a final software product but a tool to advance one's exploration of the neural and neural-astroglia networks.

## Availability and future directions

ARACHNE is available online at GitHub with the explanatory documentation at https://github.com/LeonidSavtchenko/Arachne. The program is made available with an MIT license. ARACHNE is written in a way that allows users to run it with all common remote platforms. Whilst the program is designed for the MATLAB interface, the skeleton code provided in the package allow users to modify it for Python platform. We plan to untangle kernel and the graphical interface to use the GUI or the kernel with other tools such as NEURON or PyNN.

## Supporting information

S1 File Text Box. The boot file of communication between host and remote computers.
(DOCX)

S2 File Biophysics of model. A detailed description of the biophysical model of a neural-neuroglial network, the mathematical formalism and parameters.
(DOCX)

S1 Fig Graphic user interface. Example of GUI table of model parameters.
(TIF)

S2 Fig Rules of network and synaptic engagement. (a) BSS type includes a non-uniform density of synaptic weights (*red line*) and a uniform density of connections. (b) BSD type includes a uniform distribution of synaptic weights (*red line*) and non-uniformly distributed connections. (c) *EP* for large networks has been drawn in a graphic editor. (d) *EP* for small networks is prepared by the dynamic matrix. (e) (*Top*) Diagram of synaptic connections for STDP mechanisms. (*Bottom*) Examples of static rules of STDP. (f) (*Top*) Diagram of synaptic connections for the frequency dependent plasticity. (*Bottom*) Examples of rules for the frequency dependent plasticity.
(TIF)

S1 Code The code of ARACHNE, all versions. (ZIP)

## References

[1] Bednar JA. Topographica: Building and Analyzing Map-Level Simulations from Python, C/C++, MATLAB, NEST, or NEURON Components. Frontiers in neuroinformatics. 2009;3:8 10.3389/neuro.11.008.2009 19352443 PMC2666198

[2] Nowke C, Zielasko D, Weyers B, Peyser A, Hentschel B, Kuhlen TW. Integrating Visualizations into Modeling NEST Simulations. Frontiers in neuroinformatics. 2015;9:29 10.3389/fninf.2015.00029 26733860 PMC4681776

[3] Goodman D, Brette R. Brian: a simulator for spiking neural networks in python. Frontiers in neuroinformatics. 2008;2:5 10.3389/neuro.11.005.2008 19115011 PMC2605403

[4] Vitay J, Dinkelbach HU, Hamker FH. ANNarchy: a code generation approach to neural simulations on parallel hardware. Frontiers in neuroinformatics. 2015;9:19 10.3389/fninf.2015.00019 26283957 PMC4521356

[5] Carnevale NT, Hines ML. The NEURON book. Cambridge, UK; New York: Cambridge University Press; 2006 xix, 457 p. p.

[6] Bower JM, Beeman D, Hucka M. The GENESIS Simulation System In: Arbib MA, editor. The Handbook of Brain Theory and Neural Networks. Cambridge: The MIT Press; 2003 pp. 475–478.

[7] Zenke F, Gerstner W. Limits to high-speed simulations of spiking neural networks using general-purpose computers. Frontiers in neuroinformatics. 2014;8:76 10.3389/fninf.2014.00076 25309418 PMC4160969

[8] Bekolay T, Bergstra J, Hunsberger E, Dewolf T, Stewart TC, Rasmussen D, et al Nengo: a Python tool for building large-scale functional brain models. Frontiers in neuroinformatics. 2014;7:48 10.3389/fninf.2013.00048 24431999 PMC3880998

[9] Davison AP, Bruderle D, Eppler J, Kremkow J, Muller E, Pecevski D, et al PyNN: A Common Interface for Neuronal Network Simulators. Frontiers in neuroinformatics. 2008;2:11 10.3389/neuro.11.011.2008 19194529 PMC2634533

[10] Stockton DB, Santamaria F. NeuroManager: a workflow analysis based simulation management engine for computational neuroscience. Frontiers in neuroinformatics. 2015;9:24 10.3389/fninf.2015.00024 26528175 PMC4602303

[11] Markram H, Muller E, Ramaswamy S, Reimann MW, Abdellah M, Sanchez CA, et al Reconstruction and Simulation of Neocortical Microcircuitry. Cell. 2015;163(2):456–92. 10.1016/j.cell.2015.09.029 26451489

[12] Stockton DB, Santamaria F. Automating NEURON Simulation Deployment in Cloud Resources. Neuroinformatics. 2016. 10.1007/s12021-016-9315-8 PMC5669366 27655341

[13] Thibeault CM, Minkovich K, O'Brien MJ, Harris FC Jr., Srinivasa N. Efficiently passing messages in distributed spiking neural network simulation. Frontiers in computational neuroscience. 2013;7:77 10.3389/fncom.2013.00077 23772213 PMC3677129

[14] Fidjeland AK, Roesch EB, Shanahan MP, Luk W. NeMo: A Platform for Neural Modelling of Spiking Neurons Using GPUs. Ieee Int Conf Asap. 2009:137–44.

[15] Carlson KD, Nageswaran JM, Dutt N, Krichmar JL. An efficient automated parameter tuning framework for spiking neural networks. Frontiers in neuroscience. 2014;8:10 10.3389/fnins.2014.00010 24550771 PMC3912986

[16] Eliasmith C, Stewart TC, Choo X, Bekolay T, DeWolf T, Tang C, et al A Large-Scale Model of the Functioning Brain. Science. 2012;338(6111):1202–5. 10.1126/science.1225266 23197532

[17] Effenberger F, Jost J, Levina A. Self-organization in Balanced State Networks by STDP and Homeostatic Plasticity. PLoS computational biology. 2015;11(9):e1004420 10.1371/journal.pcbi.1004420 26335425 PMC4559467

[18] Miner D, Triesch J. Plasticity-Driven Self-Organization under Topological Constraints Accounts for Non-random Features of Cortical Synaptic Wiring. PLoS computational biology. 2016;12(2). 10.1371/journal.pcbi.1004759 PMC4750861 26866369

[19] Volterra A, Meldolesi J. Astrocytes, from brain glue to communication elements: the revolution continues. Nature Reviews Neuroscience. 2005;6(8):626–40. 10.1038/nrn1722 16025096

[20] Araque A, Parpura V, Sanzgiri RP, Haydon PG. Tripartite synapses: glia, the unacknowledged partner. Trends in neurosciences. 1999;22(5):208–15. Epub 1999/05/14. 10322493 10.1016/s0166-2236(98)01349-6

[21] Haydon PG. GLIA: listening and talking to the synapse. Nature reviews Neuroscience. 2001;2(3):185–93. Epub 2001/03/21. 10.1038/35058528 11256079

[22] Volterra A, Liaudet N, Savtchouk I. Astrocyte Ca^2+^ signalling: an unexpected complexity. Nature reviews Neuroscience. 2014;15(5):327–35. Epub 2014/04/18. 10.1038/nrn3725 24739787

[23] Rusakov DA. Disentangling calcium-driven astrocyte physiology. Nature Reviews Neuroscience. 2015;16(4):226–33. 10.1038/nrn3878 25757560

[24] Bushong EA, Martone ME, Jones YZ, Ellisman MH. Protoplasmic astrocytes in CA1 stratum radiatum occupy separate anatomical domains. The Journal of neuroscience: the official journal of the Society for Neuroscience. 2002;22(1):183–92. 11756501 10.1523/JNEUROSCI.22-01-00183.2002 PMC6757596

[25] Araque A, Carmignoto G, Haydon PG, Oliet SH, Robitaille R, Volterra A. Gliotransmitters travel in time and space. Neuron. 2014;81(4):728–39. Epub 2014/02/25. 10.1016/j.neuron.2014.02.007 24559669 PMC4107238

[26] Haydon PG, Carmignoto G. Astrocyte control of synaptic transmission and neurovascular coupling. Physiol Rev. 2006;86(3):1009–31. Epub 2006/07/04. 10.1152/physrev.00049.2005 16816144

[27] Zoli M, Jansson A, Sykova E, Agnati LF, Fuxe K. Volume transmission in the CNS and its relevance for neuropsychopharmacology. Trends Pharmacol Sci. 1999;20(4):142–50. 10322499 10.1016/s0165-6147(99)01343-7

[28] Savtchenko LP, Rusakov DA. Regulation of rhythm genesis by volume-limited, astroglia-like signals in neural networks. Philosophical transactions of the Royal Society of London Series B, Biological sciences. 2014;369(1654):20130614 10.1098/rstb.2013.0614 25225103 PMC4173295

[29] Gleeson P, Steuber V, Silver RA. neuroConstruct: a tool for modeling networks of neurons in 3D space. Neuron. 2007;54(2):219–35. 10.1016/j.neuron.2007.03.025 17442244 PMC1885959

[30] Zheng K, Scimemi A, Rusakov DA. Receptor actions of synaptically released glutamate: the role of transporters on the scale from nanometers to microns. Biophysical journal. 2008;95(10):4584–96. Epub 2008/08/12. 10.1529/biophysj.108.129874 18689452 PMC2576387

[31] Sylantyev S, Savtchenko LP, Niu YP, Ivanov AI, Jensen TP, Kullmann DM, et al Electric fields due to synaptic currents sharpen excitatory transmission. Science. 2008;319(5871):1845–9. 10.1126/science.1154330 18369150 PMC2685065

[32] Savtchenko LP, Sylantyev S, Rusakov DA. Central synapses release a resource-efficient amount of glutamate. Nature neuroscience. 2013;16(1):10–2. 10.1038/nn.3285 23242311 PMC3605778

[33] Vergnano AM, Rebola N, Savtchenko LP, Pinheiro PS, Casado M, Kieffer BL, et al Zinc dynamics and action at excitatory synapses. Neuron. 2014;82(5):1101–14. 10.1016/j.neuron.2014.04.034 24908489

[34] Sylantyev S, Savtchenko LP, Ermolyuk Y, Michaluk P, Rusakov DA. Spike-driven glutamate electrodiffusion triggers synaptic potentiation via a homer-dependent mGluR-NMDAR link. Neuron. 2013;77(3):528–41. 10.1016/j.neuron.2012.11.026 23395378 PMC3568920

[35] Pavlov I, Savtchenko LP, Song I, Koo J, Pimashkin A, Rusakov DA, et al Tonic GABAA conductance bidirectionally controls interneuron firing pattern and synchronization in the CA3 hippocampal network. Proceedings of the National Academy of Sciences of the United States of America. 2014;111(1):504–9. 10.1073/pnas.1308388110 24344272 PMC3890854

[36] Harnett MT, Makara JK, Spruston N, Kath WL, Magee JC. Synaptic amplification by dendritic spines enhances input cooperativity. Nature. 2012;491(7425):599–602. 10.1038/nature11554 23103868 PMC3504647

[37] Benke TA, Luthi A, Isaac JT, Collingridge GL. Modulation of AMPA receptor unitary conductance by synaptic activity. Nature. 1998;393(6687):793–7. 10.1038/31709 9655394

[38] Gloveli T, Dugladze T, Rotstein HG, Traub RD, Monyer H, Heinemann U, et al Orthogonal arrangement of rhythm-generating microcircuits in the hippocampus. Proceedings of the National Academy of Sciences of the United States of America. 2005;102(37):13295–300. Epub 2005/09/06. 10.1073/pnas.0506259102 16141320 PMC1201613

[39] Tort AB, Rotstein HG, Dugladze T, Gloveli T, Kopell NJ. On the formation of gamma-coherent cell assemblies by oriens lacunosum-moleculare interneurons in the hippocampus. Proceedings of the National Academy of Sciences of the United States of America. 2007;104(33):13490–5. Epub 2007/08/08. 10.1073/pnas.0705708104 17679692 PMC1948921

[40] Kopell N, Borgers C, Pervouchine D, Malerba P, Tort A. Gamma and Theta Rhythms in Biophysical Models of Hippocampal Circuits. Spr Ser Comput Neuro. 2010;5:423–57.

[41] Perea G, Araque A. GLIA modulates synaptic transmission. Brain research reviews. 2010;63(1–2):93–102. 10.1016/j.brainresrev.2009.10.005 19896978

[42] Araque A, Carmignoto G, Haydon PG, Oliet SHR, Robitaille R, Volterra A. Gliotransmitters Travel in Time and Space. Neuron. 2014;81(4):728–39. 10.1016/j.neuron.2014.02.007 24559669 PMC4107238

[43] Verkhratsky A, Kirchhoff F. Glutamate-mediated neuronal-glial transmission. J Anat. 2007;210(6):651–60. 10.1111/j.1469-7580.2007.00734.x 17504269 PMC2375757

[44] Fiacco TA, McCarthy KD. Intracellular astrocyte calcium waves in situ increase the frequency of spontaneous AMPA receptor currents in CA1 pyramidal neurons. The Journal of neuroscience: the official journal of the Society for Neuroscience. 2004;24(3):722–32. 14736858 10.1523/JNEUROSCI.2859-03.2004 PMC6729258

[45] Volman V, Ben-Jacob E, Levine H. The astrocyte as a gatekeeper of synaptic information transfer. Neural computation. 2007;19(2):303–26. Epub 2007/01/09. 10.1162/neco.2007.19.2.303 17206866

[46] Amdahl GM. Computer Architecture and Amdahl's Law. Computer. 2013;46(12):38–46.

[47] Holmgren C, Harkany T, Svennenfors B, Zilberter Y. Pyramidal cell communication within local networks in layer 2/3 of rat neocortex. The Journal of physiology. 2003;551(Pt 1):139–53. Epub 2003/06/19. 10.1113/jphysiol.2003.044784 12813147 PMC2343144

[48] Fuxe K, Agnati LF, Marcoli M, Borroto-Escuela DO. Volume Transmission in Central Dopamine and Noradrenaline Neurons and Its Astroglial Targets. Neurochemical research. 2015. Epub 2015/04/22. 10.1007/s11064-015-1574-5 25894681

