# Supplementary material for: ARACHNE: A neural-neuroglial network builder with remotely controlled parallel computing
Source: PLoS Comput Biol. 2017 Mar 31;13(3):e1005467. doi: 10.1371/journal.pcbi.1005467 (PMC5393895; doi:10.1371/journal.pcbi.1005467)
Supplement: S1 File — The boot file of communication between host and remote computers. (DOCX) [file pcbi.1005467.s001.docx]

## Boot file with parameters of initial simulation sent from a host computer to a remote computer

| @echo off |  |
| --- | --- |
|  |  |
|  | rem Run this BAT-script with one argument: |
|  | rem 1) the flag (0/1) indicating whether to compress MAT-file before and decompress after uploading, |
|  | rem 2) the MAT-file name (without the extension). |
|  |  |
|  | rem Initialize variables |
|  | call Code\scripts\win-lin\params.bat |
|  |  |
|  | set SOURCEFILE=%2 |
|  |  |
|  | rem Go to Matlab host directory |
|  | cd %MATLABHOSTDIR% |
|  |  |
|  | if %1 == 1 ( |
|  | rem Check if an old archive exists, if so -- delete |
|  | if exist %SOURCEFILE%.zip del %SOURCEFILE%.zip |
|  |  |
|  | rem Compress MAT-file |
|  | "%THIRDPARTYDIR%\7za.exe" a %SOURCEFILE%.zip %SOURCEFILE%.mat > NUL |
|  |  |
|  | rem The ZIP-archive will be uploaded instead of the MAT-file |
|  | set FILENAME=%SOURCEFILE%.zip |
|  | ) else ( |
|  | rem The MAT-file will be uploaded itself |
|  | set FILENAME=%SOURCEFILE%.mat |
|  | ) |
|  |  |
|  | rem Go to 3rd party software directory containing pscp.exe and plink.exe |
|  | cd %THIRDPARTYDIR% |
|  |  |
|  | rem Upload MAT-file or ZIP-file to the head node of HPC cluster |
|  | pscp -pw %PASSWORD% "%MATLABHOSTDIR%\%FILENAME%" %LOGIN%@%HEADNODEIP%:"%HEADNODEWORKERDIR%/iofiles/host-kernel" |
|  |  |
|  | if %1 == 1 ( |
|  | rem Unzip MAT-file and remove archive |
|  | plink -pw %PASSWORD% %LOGIN%@%HEADNODEIP% cd \"%HEADNODEWORKERDIR%/iofiles/host-kernel\"; rm %SOURCEFILE%.mat -f; unzip %SOURCEFILE%.zip > NUL; rm %SOURCEFILE%.zip -f; |
|  |  |
|  | rem Go to Matlab host directory. |
|  | rem (Remark: do not remove the quotes!) |
|  | cd "%MATLABHOSTDIR%" |
|  |  |
|  | rem Delete just uploaded archive |
|  | del %SOURCEFILE%.zip |
|  | ) |

## File of commands to run a simulation.

| @echo off |  |
| --- | --- |
|  |  |
|  | rem Run this BAT-script with six or seven arguments specifying: |
|  | rem 1) the number of processes, |
|  | rem 2) the number of threads per process, |
|  | rem 3) the flag (0/1) indicating whether previous simulation session should be continued, |
|  | rem 4) the number of iterations or the time interval (in ms) to compute in this simulation session, |
|  | rem 5) the flag (0/1) indicating whether the time interval (0) or the number of iterations (1) is specified, |
|  | rem 6) list of comma-separated cluster node names to run simulation on, |
|  | rem 7) (optional) the flag (0/1) indicating whether to disable STDP for this simulation session (image recall mode). |
|  |  |
|  | rem Initialize variables |
|  | call Code\scripts\win-lin\params.bat |
|  |  |
|  | rem Go to 3rd party software directory containing plink.exe |
|  | cd %THIRDPARTYDIR% |
|  |  |
|  | rem Execute two commands on remote server: |
|  | rem 1) go to HPC kernel directory, |
|  | rem 2) run helper SH-script in background mode. |
|  | plink -pw %PASSWORD% %LOGIN%@%HEADNODEIP% "cd \"%REMOTESCRIPTSDIR%\"; sh run.sh %1 %2 %3 %4 %5 %6 %7" |

## File of uploading of results to the local computers.

| @echo off |  |
| --- | --- |
|  |  |
|  | rem Run this BAT-script with two arguments: |
|  | rem 1) the flag (0/1) indicating whether file "output.mat" should be taken from the snapshot directory, |
|  | rem 2) the flag (0/1) indicating whether to compress MAT-file before and decompress after downloading. |
|  |  |
|  | rem Initialize variables |
|  | call Code\scripts\win-lin\params.bat |
|  |  |
|  | rem Go to 3rd party software directory containing plink.exe, pscp.exe and 7za.exe |
|  | cd %THIRDPARTYDIR% |
|  |  |
|  | if %1 == 1 ( |
|  | set "DIR=/snapshot" |
|  | ) else ( |
|  | set "DIR=" |
|  | ) |
|  |  |
|  | if %2 == 1 ( |
|  | rem Compress file output.mat |
|  | plink -pw %PASSWORD% %LOGIN%@%HEADNODEIP% cd \"%HEADNODEWORKERDIR%/iofiles/kernel-host%DIR%\"; rm output.zip -f; zip output.zip output.mat > NUL; |
|  |  |
|  | rem The archive output.zip will be downloaded instead of the file output.mat |
|  | set FILENAME=output.zip |
|  | ) else ( |
|  | rem The file output.mat will be downloaded itself |
|  | set FILENAME=output.mat |
|  | ) |
|  |  |
|  | rem Download output MAT-file or ZIP-file from the head node of HPC cluster |
|  | pscp -pw %PASSWORD% %LOGIN%@%HEADNODEIP%:"%HEADNODEWORKERDIR%/iofiles/kernel-host%DIR%/%FILENAME%" "%MATLABHOSTDIR%" |
|  |  |
|  | if %2 == 1 ( |
|  | rem Go to Matlab host directory. |
|  | rem (Remark: do not remove the quotes!) |
|  | cd "%MATLABHOSTDIR%" |
|  |  |
|  | rem Delete old output MAT-file |
|  | if exist output.mat del output.mat |
|  |  |
|  | rem Unzip just downloaded MAT-file |
|  | "%THIRDPARTYDIR%"\7za.exe e output.zip > NUL |
|  |  |
|  | rem Delete the archive on local machine |
|  | del output.zip |
|  |  |
|  | rem Go to 3rd party software directory |
|  | cd "%THIRDPARTYDIR%" |
|  |  |
|  | rem Delete the archive on remote machine |
|  | plink -pw %PASSWORD% %LOGIN%@%HEADNODEIP% cd \"%HEADNODEWORKERDIR%/iofiles/kernel-host%DIR%\"; rm output.zip -f; |
|  | ) |
