# Supplementary material for: ARACHNE: A neural-neuroglial network builder with remotely controlled parallel computing
Source: PLoS Comput Biol. 2017 Mar 31;13(3):e1005467. doi: 10.1371/journal.pcbi.1005467 (PMC5393895; doi:10.1371/journal.pcbi.1005467)
Supplement: S2 File — A detailed description of the biophysical model of a neural-neuroglial network, the mathematical formalism and parameters. (DOCX) [file pcbi.1005467.s002.docx]

ARACHNE: A neural-neuroglial network builder with remotely controlled parallel computing

**Basic pre-installed biophysical model of networks**

Basic model of networks pre-installed in ARACHNE

**Network organization: Topology**

Similar to a great majority of previously explored non-hierarchical networks [1], the basic connectivity is represented by circle (Fig 1 B). This (a) enables full cell inter-connectivity, without edge effects or specific boundary conditions, (b) makes all cell locations a priori equivalent, and (c) can be described by a single size parameter, radius *R*.

The geometrical sizes of the ***i***- and ***e***-neuronal networks can vary independently. In contrast, the ***a-*** and ***e-*** networks have similar sizes and are positioned 'next' to each other. The latter reflects the fact that, throughout area CA1 of the hippocampus, pyramidal (excitatory) cells are arranged in a regular layer and surrounded by a relatively uniform scatter of astrocytes [2]. Accordingly, modelled astrocytes are distributed evenly across the e-network, with a fixed total cell number (for simplicity it is equal to the number of e-neurons in our case study). Such arrangement is to represent diffused (volume-transmitted) astrocyte-neuron signalling without loss of generality (Fig1 C).

**Network organization: Synaptic strengths and their distributions.**

The two neuronal networks, ***i*** and ***e***, have four types of synaptic connections: ***ee, ei, ie, ii*** (Fig 1 B) described as in [3]. For each type, ***ee, ei, ie, ii,*** the model provides several key parameters, such as synaptic strength ***w***, synaptic current rise time ***τ_r_*** and decay time ***τ_d_***, and probability of neurotransmitter release *p*. All connection strength values between *i*th and *j*th neurons, at any given time, comprise the classical connectivity matrix. One distinct and novel feature of the networks is that the value of *p* can be modified by the activity of nearby astrocytes, in accord with the current view in the field of glia-neuron signalling [4]. The synaptic strength ***w*** can also be modified by three mechanisms of synaptic plasticity incorporated in the model, described in the sections below.

In the brain, the synaptic strength ***w*** appears to depend on the distance between cortical neurons [5]: The model provides two complementary types of connectivity to imitate this observation. The first type, termed bell-shaped strength (*BSS*) model, incorporates a Gaussian distribution of ***w*** (centred at the 'presynaptic' cell) with uniform connection density between the nearest 50% of all network neurons and standard deviation σ (S2 Fig A). The second type, a bell-shaped density (*BSD*) model, incorporates uniform distribution of ***w*** but a Gaussian distribution of cell-cell connection density (S2 Fig B), where the number of connections decrease with distance from the 'presynaptic' cell.

In addition to having individual synaptic currents, the model enables tonic excitation or inhibition currents, a rapidly emerging feature of hippocampal networks [6].

It is important to note that the user can easily change the type of astroglia and neurons, synapses and their distribution type, types of plasticity and other biophysical parameters, using only a GUI (see S1 Fig A, for details) without changing the kernel located on a server.

**Model equations for membrane potential of neurons**

Dynamics of neuronal membrane potential *V* is described by a set of equations with the Hodgkin–Huxley formalism:

$$C\frac{dV}{dt}\text{ }=\text{ }I_{app}-I_{Na}-I_{K}-I_{L}-I_{syn}-I_{extra}$$

The computational kernel calculates the set of Eq. , where *I_Na_* and *I_K_* are the sodium and potassium currents respectively, *I_app_* is the current used to simulate the dynamics of external excitation, *I_L_* is the leaking current, *I_syn_* is the sum of synaptic currents. The *I_Na_* and *I_K_* are simulated using a previously reported approach [7-9]. The С++ code for calculating the Eq. with the kinetics for *i‑*neurons and *e‑*neurons was adopted from NEURON database (https://senselab.med.yale.edu; ModelDB accession no. 138421).

In addition to all specific currents, the model includes the GUI (S1 Fig) table allowing the introduction of any non-specific current (tonic or phasic) *I_extra_*. The latter is given by expression

$$I_{extra}=-g_{extra} a_{e}^{p_{j}} b_{e}^{q_{j}}(V-V_{re})$$

with the maximum conductance *g_extra_*, the activation variable *a_e_*, the inactivation variable *b_e_* and the reversal potential *V_re_*. The powers *p_j_* and *q_j_* represent the number of gating states of the ionic channels and are integers between 0 and 4 inclusively. Variables *a_e_* and *b_e_* are assumed to obey the first-order ordinary differential equation

$$\frac{dx}{dt}=k_{x}(V)(-x+x_{\infty}(V)), (x=a_{e},b_{e})$$

The steady state values of *x_∞_*(V) are sigmoid functions of the membrane potential

$$x_{\infty}(V)=\frac{1}{1+exp(-s_{x}(V-V_{x}))}, (x=a_{e},b_{e})$$

where *V_x_* and *s_x_* represent the threshold and the slope of a steady state curve, respectively. The rate coefficient *k_x_*(V) has the following voltage dependency:

$$k_{x}(V)=\frac{1}{t_{x}}\cosh\left(\frac{s_{x}(V-V_{x})}{2}\right), (x=a_{e},b_{e})$$

where *t_x_* is a time constant.

The software provides a possibility to modify the kinetics of channels using a GUI. In non-excitable astrocytes, the membrane potential *V* is set to a constant value, whereas the main dynamic variable is the intracellular calcium concentration.

ARACHNE also provides the option to add mod-files for currents generated by NEURON software tools.

**Model equations for synaptic currents**

Chemical synapses have conductances, release probabilities and spatial distributions. The synaptic current in the model is defined as *I = g_n_s_n_*(*V-V_nr_*)*,* where *n* = 1,...,4 denotes one of the four synaptic types - *ee, ii, ei,* or *ie*, respectively, *V_nr_* is a reverse potential*, g_n_* and *s_n_* are the synaptic conductance and the kinetics is defined [3] as:

$$\left(s_{EE}\right)_{j}=\sum_{k=0}^{Ne-1} s_{Ek}E_{j}$$

$$\left(s_{IE}\right)_{j}=\sum_{k=0}^{Ni-1} s_{Ik}E_{j}$$

$$\left(s_{EI}\right)_{j}=\sum_{k=0}^{Ne-1} s_{Ek}I_{j}$$

$$\left(s_{II}\right)_{j}=\sum_{k=0}^{Ni-1} s_{Ik}I_{j}$$

where

,

The variable *v* is dimensionless.

Eq. describes synapses from *e*-neurons to *R_j_-*neurons *(*where *R_j_* is either *e_j_-cell* or *i_j_* -cell depending on the postsynaptic cell), while Eq. describes synapses from *i*-neurons to *R_j_–*neurons*.* The total synaptic current received by a single neuron is dependent on the number of neurons in the network and the synaptic distributions.

**Equation for tonic currents**

Experimental data revealed [6, 10, 11] that the dynamics of the networks depend on the [GABA]_ex_, an inhibitory neurotransmitter. The [GABA]_ex_ activates the extrasynaptic receptors which generate the tonic currents. To take into account this mechanism, the neurons in the model include the following non-specific tonic current, *I_tonic_* Eq.:

$$I_{tonic}=G_{tonic}(V_{tonic}-V)$$

where *G_tonic_* is the tonic conductance, *V_tonic_* is the reverse potential of tonic current and *V* is the membrane potential. The non-specificity means that the user can set either excitatory or inhibitory tonic current.

Model affords two options for the tonic conductance: a constant conductance and an activity-dependent conductance.

In the brain, the extracellular neurotransmitter concentration varies depending on the frequency of synaptic release and the ion pumps activity. In turn, the extracellular modification of the neurotransmitter concentration affects the extrasynaptic receptor activations and thus modifies neuronal excitability and the frequency of synaptic release. To take into account the feedback between neurotransmitter concentration and network activity, a biophysical mechanism is added to the basic model, which relates the tonic current conductance to the firing frequency of i-neurons Eq..

Mathematically, the dynamics of *G_tonic_* is described by the following equations:

$\frac{dG_{tonic}}{dt}=N_{i} A_{f}(f_{m}[t-dt]+f_{b})-t_{p}(G_{tonic}-G_{b})$,

The critical dynamic variable of the equation is the i-neurons network firing frequency Eq.:

$$f_{m}=\frac{1}{T N_{i}}\sum_{i=1}^{N_{i}} AP_{i}(T,t)$$

The variable, *f_m_* is the average frequency of the *i-*neuron network calculated as a sum of all APs generated by all *i-*neurons within the time frame of *T*.

Other parameters are: *N_i_* is the number of neurons in the network, *A_f_* is the change of the tonic conductance as a single i-neuron generates a single AP during time *T*, *t_p_* is the rate of neurotransmitter uptake, *G_b_* is the reverse concentration of neurotransmitter uptake, *f_b_* is the basic frequency of network at resting state, the parameter *dt* is the time delay between the release of neurotransmitter and the activation of extrasynaptic receptors.

The main assumption of the equation is that the tonic conductance is linearity depended on the concentration of neurotransmitter.

With this biophysical mechanism, the model enables a feedback between the tonic current conductance and the network dynamics, as we have shown earlier using simpler network configurations [10].

**Equations for astrocyte network**

The astrocytic intracellular calcium dynamic outlined in [12] is described by the following equation :

$$\frac{dCa}{dt}=-J_{c}-J_{p}-J_{l}-J_{syn}$$

where $J_{p}=\frac{v_{3}Ca^{2}}{k_{3}^{2}+Ca^{2}}$is the flux through SERCA pumps, $J_{l}=c_{1}v_{2}(Ca-Ca_{ER})$ is the leak from the endoplasmic reticulum into the cytosol, $J_{syn}=g_{s}(Ca-Ca_{l})+g_{s}(Ca-Ca_{r})$ is the calcium fluxes through the gap junctions generated by the nearest left and right astrocytes, $J_{c}=Cv_{1}m_{\infty}^{3}n_{\infty}^{3}q^{3}(Ca-Ca_{ER})$ is a calcium-dependent current from the intracellular store, where *q* is the fraction of active IP3 receptors and *g_s_* is the conductance of the gap junction between astrocytes.

The parameters of the intracellular stores are defined by the following equations:

$$\begin{matrix} \frac{dq}{dt}=\alpha_{q}(1-q)-\beta_{q}q, m_{\infty}=\frac{P}{P+d_{1}}, n_{\infty}=\frac{Ca}{Ca+d_{5}} \\ \alpha_{q}=a_{2}d_{2}\frac{P+d_{1}}{P+d_{3}}, \beta_{q}=a_{2}Ca, Ca_{ER}=\frac{c_{0}-Ca}{c_{1}} \end{matrix}$$

Given that IP3 degrades with a time constant *τ_p_*, an equation for cytosolic IP3 is needed, specified as

,

where [*y*] is concentration of neurotransmitter release from the synapse, *r_ip_*_3_ [mM s^-1^] is the rate of IP3 release.

Local concentration of neurotransmitters [y] depends on the spike generation by nearest neurons and the uptakes by the pump. The dynamic is described by the following equation

$$\frac{d[y]}{dt}=\tau_{spike}\delta\left(t-t_{spike}\right)-t_{p}[y]$$

Where *τ*_spike_ is the time scale of the spikes and *t_p_* is the time course of uptake.

The calcium concentration of an astrocyte, [*Ca*]*_astro_,* influences the release probability of a nearby neuronal synapse according to the equation:

$p=p_{basic}+p_{Max}\left(1\text{ }-\frac{\left[Ca\right]_{ss}}{\left[Ca\right]_{astro}}\right)$*,*

where *p* is the release probability of the synapse from e-neuron to i-neuron and it is already defined in the basic model. The probability of synaptic release from i-neuron to e-neuron can be added to the model by user.

Astrocytes interact through the gap junctions as:

$J=\frac{([Ca]_{i}-[Ca]_{j})}{\tau_{j}}$*,*

where [Ca]_i_ and [Ca]_j_ are the calcium concentrations in the nearest astrocytes,*τ_a_* is the time constant of spatial relaxation of calcium fluxes between these astrocytes.

**Release probability distribution**

In the basic model, hetero- and homogeneous spatial distributions of the release probability *p* have been incorporated. The heterogeneous *p* pattern which is typical for excitatory synapses on CA1 pyramidal neurons [13] could be applied to the synapses make connections between the *ei* and *ie* networks. The ability to vary the spatial distribution of *p* is important for exploring the space-delimited influence of astrocytes on synaptic transmission [1].

The value of ***p*** can range between zero and one, which could be set manually or can be made dependent on the calcium activity of astrocytes.

**Setting the external input**

In the model, which has no hierarchical structure (for the sake of generality), external (sensory) input can be mimicked by a distributed excitatory stimulation of e-cells [14]. The network-wise distribution of such stimuli is given by the input's external pattern *(EP)* with the adjustable current amplitude. For small networks, the *EP* can be set using the method of a dynamic matrix (S2 Fig D), with matrix elements representing activated (black) or non-activated (white) neurons. For large networks, when the matrix size is huge, the *EP* can be defined using a binary drawing (S2 Fig C) with black pixels depicting activated ***e***-neurons. The total size of the *EP* in pixels, or the size of dynamic matrix, thus represents the total number of ***e***-neurons.

In the standard network memory paradigm, the brain circuits memorize a particular *EP* through the activity-induced changes in synaptic weights which forms a new connectivity (synaptic weight) matrix.

**Adopted mechanisms of synaptic plasticity**

To simulate the processes of memorization, the model includes three common mechanisms of synaptic plasticity: (a) spike-timing dependent plasticity (STDP), (b) frequency-dependent plasticity, and (c) synaptic plasticity due to activation of a neighbouring astrocyte.

STDP is a well-established [15] form of plasticity that occurs upon temporal coincidence of the postsynaptic action potential and the presynaptic synaptic discharge. The actual STDP rules vary widely depending on the type of neuron and its connections [16]. In the model, each type of the synaptic connection can have its own rule to modify synaptic strength (S2 Fig E and F).

It has long been suggested that presynaptic spiking occurring at lower or higher frequencies could, respectively, potentiate or depress synaptic efficacy [17]. To take the observation into account, the model provides a plasticity mechanism accounting for linear frequency-dependent regulation of synaptic strength (S2 Fig F).

A distinct mechanism of synaptic plasticity implemented in the model deals with modifications of *p* due to calcium-dependent astrocyte activity [18, 19]. The model is set so that when the calcium level inside an astrocyte exceeds a chosen threshold, the astrocyte 'releases' signalling molecules (such as ATP or adenosine) that affect *p* at the neighbouring ***ei***‑synapses in a tissue volume [19] see “Equations of astrocyte network”. Model provides an opportunity to switch off the astrocyte network thus keeping *p* unchanged throughout.

**Equation for the spike-time dependent plasticity (STDP)**

To fit a wide range of STDP rules into a single instruction, the following formula was introduced:

$$\frac{dW}{W}=A_{+}\left(1-\frac{1}{t}\right)^{S_{1}}+A_{-}\left(1-\frac{1}{t}\right)^{S2}+ A_{c}e^{t_{c}Abs\left(t\right)}\cos\left(C_{p}t\right)+A_{s}e^{t_{s}Abs\left(t_{a}(t+dt)\right)} \sin(S_{p}(t+dt))$$

where *W* and *dW* are the synaptic weigh and its modification, *t* is a time difference between the presynaptic and postsynaptic APs. Parameters *A_+_, A-, Ac* and *As* define the amplitude of *dW*. The model is designed so that for a given synapse there is only one non-zero amplitude parameter. This method allows the type of plasticity for a given synaptic connection to be chosen by using only parameters stored on the host computer and leaving the compiled computational code on the cluster unchanged.

Parameters *t_c_, t_s_, S*_1_*, S*_2_*, C_p_* and *S_p_* characterise the time course of STDP and *t_a_* is nondimensional scaling parameter.

**Equations for frequency-dependent plasticity**

The model provides a frequency dependent regulation of synaptic strength. The linear approximation between the synaptic strength *W* and the presynaptic and the postsynaptic frequencies *ω_pre_* and *ω_post_* is observed in different areas of the brain and described by a simple equation Eq.

$$\frac{\Delta W}{W}=\Delta t \tau\left(\alpha\omega_{post}-\beta\omega_{pre}\right)$$

where Δ*W* is the synaptic strength modification, which occurs in time Δ*t*; *α* and *β* set the synaptic strength sensitivity to the presynaptic and postsynaptic frequencies, respectively; and τ is the characteristic time of synaptic modification.

1. All-inclusive model of synaptic modifications

Classically, experimental studies documented activity-dependent alterations in synaptic weighting as an individual event statistically validated within a specific time window. In most cases, the temporal dynamics of synaptic efficacy change are either neglected or unattainable through reliable measurements. However, the characteristic time interval over which synaptic modifications continue to change ("remembered") is a key parameter in memorizing the external input to the neural network [14]. The present model enables the setting of a dynamic time-dependent modification of synaptic weights with the following integral-differential equation:

$$\frac{dw_{\text{ij}}}{dt}= \left[\tau\left(\alpha\omega_{\text{post}}(t)-\beta\omega_{pre}(t)\right)+\int_{t-T}^{t} d\tau_{1}\int_{t-T}^{t} d\tau_{2}\left\|\text{v}_{\text{i}}\left(\tau_{1}\right),\text{v}_{\text{j}}\left(\tau_{2}\right)\right\|\Delta\tilde{W}\left(\tau_{1}-\tau_{2}\right)\varepsilon\left(t-\tau_{1},T\right)\varepsilon\left(t-\tau_{2},T\right)\right]w_{\text{ij}}$$

where the time bin *T* defines an interval of correlation between times of presynaptic and postsynaptic APs.

The correlation function between APs is $\left\|\text{v}_{\text{i}}\left(\tau_{1}\right),\text{v}_{\text{j}}\left(\tau_{2}\right)\right\|=\sigma\left(\text{v}_{\text{i}}\left(\tau_{1}\right)\right)\cdot\sigma\left(\text{v}_{\text{j}}\left(\tau_{2}\right)\right)$, where $\sigma\left(x\right)=\frac{1}{1+\exp\left(-kx\right)}$ $\left(k>>1\right)$. The function provides selection of spike pairs so that the integral becomes valuable only in the vicinities of points (*τ*_1_, *τ*_2_) means that the integral has significant value only when *τ*_1_ and *τ*_2_ are within certain intervals depending on *k*.

The discrete version of Eq. is

, $\Delta t=t_{\text{k}}-t_{\text{k}-1}$.

The sum is taken for all pairs of spikes in the interval (*t*_k-1_*­* ‒ *T*, *t*_k-1_)

To quickly adapt experimental STDP curves to the model networks, the software provides a utility that calculates and visualizes the STPD curve base on inputted parameters, against the experimental data.

1. Modification of release probability due to activation of astrocyte

Astrocytes and neurons communicate via a special mechanism modulated by neurotransmitters from both sides. A distinct mechanism of synaptic plasticity is implemented in the model to deal with modifications of synaptic release probability due to a form of calcium-dependent astrocyte neurotransmission [18]. The model is designed so that when the calcium level inside an astrocyte exceeds a threshold, the astrocyte “releases” neuromodulator (such as ATP or adenosine) that may affect the release probability at neighbouring connections in a tissue volume [19]. The corresponding formulas are described in the “**Equations for astrocyte network”** section. The astrocyte network can also be switched off altogether, in which case the release probability among synapses will remain unchanged.

**The network recall**

The recall is a process associated with the network's response to the repeated *EP (external pattern)*. The network response is a combination of spiking (active) and non-spiking (passive) neurons. The quality of recall, ***C,*** is defined by the correlation between recalled and 'perfect' responses to the *EP*:

$$C=\frac{\sum_{i=1}^{m} x_{i}y_{i}+\sum_{j=m+1}^{n} x_{j}y_{j}}{\sqrt{(\sum_{i=1}^{m} x_{i}^{2}+\sum_{j=m+1}^{n} x_{j}^{2})(\sum_{i=1}^{m} y_{i}^{2}+\sum_{j=m+1}^{n} y_{j}^{2}})}$$

where *n* is total number of neurons of the network, *m* is a the maximum number of neurons in the active state of ideal response, *x* and *y* denote the state of any neuron of ideal and real responses correspondently.

After simplification of Eq., the *C* of a network with a single stored image is:

$$C=\frac{l}{\sqrt{m(l+k)}}$$

where ***l*** and ***k*** are numbers of active “correctly” and “incorrectly” activated neurons in the recalled response, respectively; ***m*** is number of active neurons in the ideal response. The condition of ***m*** = 0 in Eq. implies total darkness for networks, i.e. an input image array of nothing.

**Basic set of parameters**

Number of i-neurons is 100 and e-neurons is 200.

Radius of i-networks is 200 μm and e-networks is 250 μm.

Rate of signal propagation between neurons is 0.1 mm/ms.

Basic release probability of all synapses were set at p***_basic_*** = 0.2, p_max_ = 0.5 is the maximum release probability which can be reached by the synapse at any calcium concentration, and [Ca]ss = 100 nM is the resting calcium concentration of astrocyte.

Standard deviation of synaptic distribution are σ_ee_= 10 neurons, σ_ei_= 12.5 neurons, σ_ie_= 8 neurons, σ_ii_= 11 neurons.

The single synaptic conductances of the basic model are *g_ie_*=0.23 μS, *g_ei_*=0.29 μS, *g_ii_*=0.64 μS, *g_ee_*=4 nS.

Rise times are τ_e_=0.1 ms and τ_i_=0.3 ms, decay times are d_e_=3 ms and d_i_=9 ms.

The basic set of the parameters of the tonic current are *A_f_* = 0. 01 μS/cm^2^*, t_p_* = 0.02 ms^‑1^, *G_b_* =0.1 μM, *f_b_* = 0.1 Hz, *dt*=0.02 μs, *T*= 100 ms.

The parameters of astrocyte dynamics used in the model were adapted from [12]

$$\begin{matrix} v_{1}=6 s^{-1}, & k_{3}=0.1 \mu M, & d_{3}=0.9434 \mu M, & c_{0}=2.0 \mu M, & v_{2}=0.11 s^{-1} \\ d_{1}=0.13 \mu M, & d_{5}=0.08234 \mu M, & c_{1}=0.185 & \tau_{p}=7 s & v_{3}=0.9 \mu M s^{-1}, \\ d_{2}=1.049\mu M, & a_{2}=0.2\mu M^{-1} s^{-1}, & P_{c}=0.16 & r_{p}=7.2 mM s^{-1}, & y=0 \end{matrix}$$

The parameters of link between astrocyte and neurons are *τ*_spike_= 2 mM/ms and *t_p_* = 0.02 ms^-1^.

These are the basic set used in the model and can be easily altered using the GUI.

**Equations for extracellular electric field**

Electrical dynamics of brain networks is mainly registered using a field electrode located in the extracellular space. The electrode senses an extracellular current density generated by a population of neurons scattered around. Because field recordings are an essential method for neuroscientists, the basic model includes a mechanism to calculate the extracellular field at a given radial coordinate with respect to the centre of the network.

For simplicity, a model of the extracellular field generated by a single neuron is simulated as a monopole electrical current source between the neuron and the ground:

, where *I*(*r*) is density of transmembrane currents $C_{m}\frac{d\vec{V}_{e}}{dt}$ of the neurons ***e*,**,and $C_{m}\frac{d\vec{V}_{i}}{dt}$ of the neurons ***i***, *r* is the distance between the neuron and electrode, and *σ* is conductance of extracellular medium.

The electrical field generated by the network is

Model allows calculating the electric field for any single network as well as a superposition of both networks.

**References of Supplementary material**

1. Savtchenko LP, Rusakov DA. Regulation of rhythm genesis by volume-limited, astroglia-like signals in neural networks. Philosophical transactions of the Royal Society of London Series B, Biological sciences. 2014;369(1654):20130614.

2. Ferrante M, Ascoli GA. Distinct and synergistic feedforward inhibition of pyramidal cells by basket and bistratified interneurons. Frontiers in cellular neuroscience. 2015;9.

3. Olufsen MS, Whittington MA, Camperi M, Kopell N. New roles for the gamma rhythm: population tuning and preprocessing for the Beta rhythm. Journal of computational neuroscience. 2003;14(1):33-54.

4. Araque A, Carmignoto G, Haydon PG, Oliet SHR, Robitaille R, Volterra A. Gliotransmitters Travel in Time and Space. Neuron. 2014;81(4):728-39.

5. Holmgren C, Harkany T, Svennenfors B, Zilberter Y. Pyramidal cell communication within local networks in layer 2/3 of rat neocortex. The Journal of physiology. 2003;551(Pt 1):139-53.

6. Kochubey S, Semyanov A, Savtchenko L. Network with shunting synapses as a non-linear frequency modulator. Neural networks : the official journal of the International Neural Network Society. 2011;24(5):407-16.

7. Gloveli T, Dugladze T, Rotstein HG, Traub RD, Monyer H, Heinemann U, et al. Orthogonal arrangement of rhythm-generating microcircuits in the hippocampus. Proceedings of the National Academy of Sciences of the United States of America. 2005;102(37):13295-300.

8. Tort AB, Rotstein HG, Dugladze T, Gloveli T, Kopell NJ. On the formation of gamma-coherent cell assemblies by oriens lacunosum-moleculare interneurons in the hippocampus. Proceedings of the National Academy of Sciences of the United States of America. 2007;104(33):13490-5.

9. Kopell N, Borgers C, Pervouchine D, Malerba P, Tort A. Gamma and Theta Rhythms in Biophysical Models of Hippocampal Circuits. Spr Ser Comput Neuro. 2010;5:423-57.

10. Pavlov I, Savtchenko LP, Song I, Koo J, Pimashkin A, Rusakov DA, et al. Tonic GABAA conductance bidirectionally controls interneuron firing pattern and synchronization in the CA3 hippocampal network. Proceedings of the National Academy of Sciences of the United States of America. 2014;111(1):504-9.

11. Song I, Savtchenko L, Semyanov A. Tonic excitation or inhibition is set by GABA(A) conductance in hippocampal interneurons. Nature communications. 2011;2:376.

12. Volman V, Ben-Jacob E, Levine H. The astrocyte as a gatekeeper of synaptic information transfer. Neural computation. 2007;19(2):303-26.

13. Dobrunz LE, Stevens CF. Heterogeneity of release probability, facilitation, and depletion at central synapses. Neuron. 1997;18(6):995-1008.

14. Kitano K, Fukai T. Temporal characteristics of the predictive synchronous firing modeled by spike-timing-dependent plasticity. Learn Mem. 2004;11(3):267-76.

15. Froemke RC, Dan Y. Spike-timing-dependent synaptic modification induced by natural spike trains. Nature. 2002;416(6879):433-8.

16. Caporale N, Dan Y. Spike timing-dependent plasticity: a Hebbian learning rule. Annual review of neuroscience. 2008;31:25-46.

17. Saviane C, Savtchenko LP, Raffaelli G, Voronin LL, Cherubini E. Frequency-dependent shift from paired-pulse facilitation to paired-pulse depression at unitary CA3-CA3 synapses in the rat hippocampus. J Physiol-London. 2002;544(2):469-76.

18. Henneberger C, Papouin T, Oliet SH, Rusakov DA. Long-term potentiation depends on release of D-serine from astrocytes. Nature. 2010;463(7278):232-6.

19. Navarrete M, Araque A. Endocannabinoids potentiate synaptic transmission through stimulation of astrocytes. Neuron. 2010;68(1):113-26.
