# Supplementary figures and images for: ARACHNE: A neural-neuroglial network builder with remotely controlled parallel computing
Source: PLoS Comput Biol. 2017 Mar 31;13(3):e1005467. doi: 10.1371/journal.pcbi.1005467 (PMC5393895; doi:10.1371/journal.pcbi.1005467)

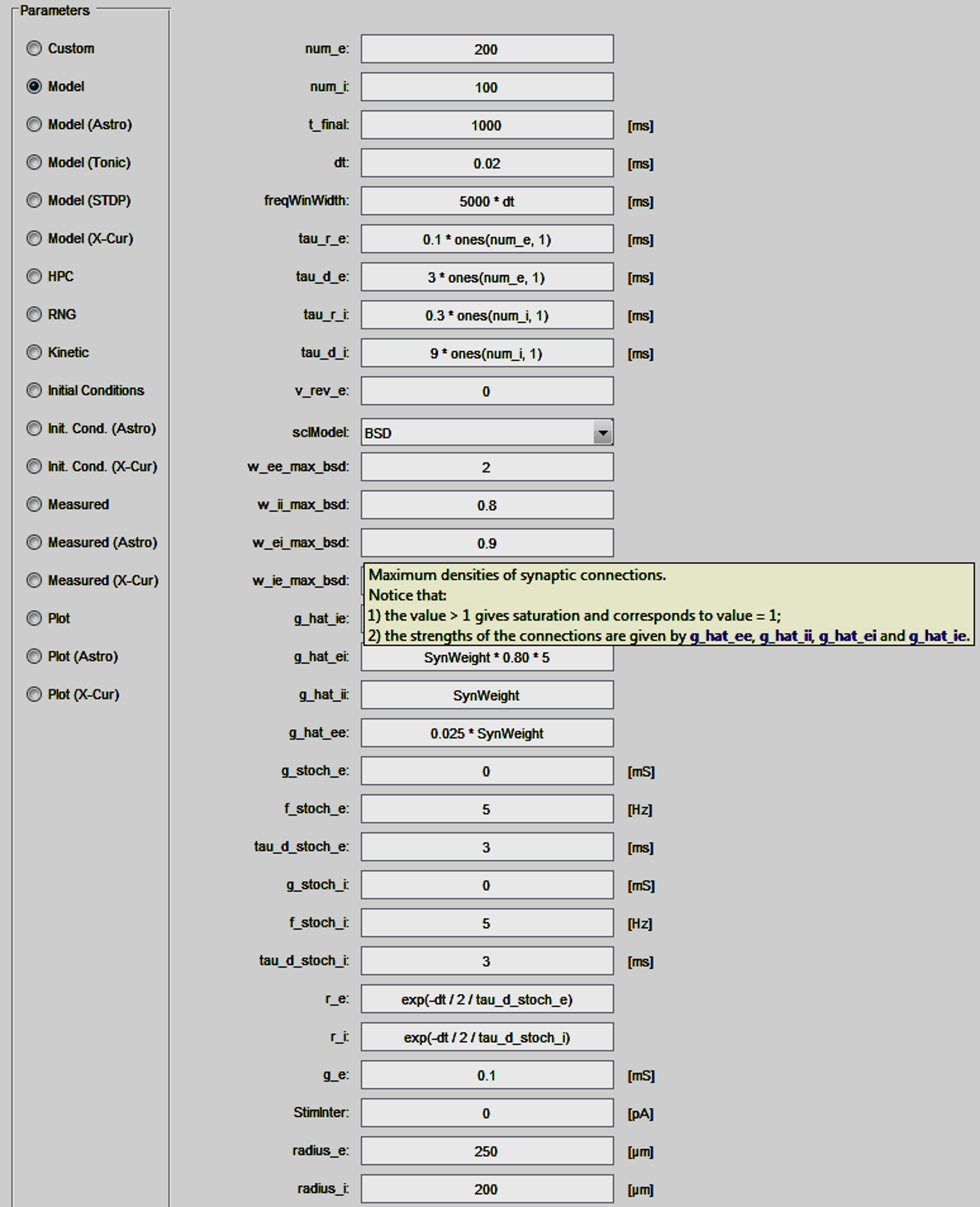

Supplement: S1 Fig — Example of GUI table of model parameters. (TIF) [file pcbi.1005467.s003.tif]

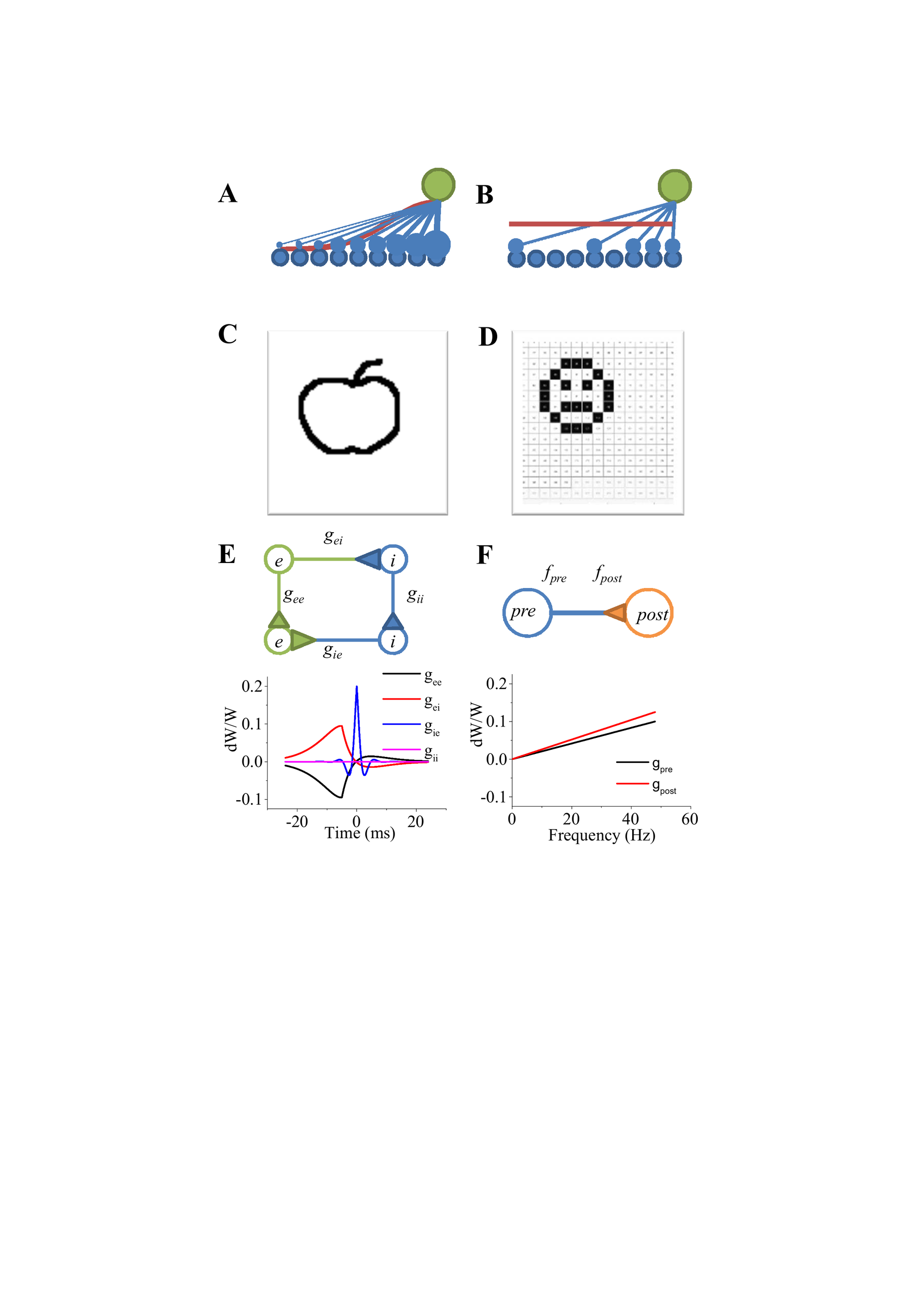

Supplement: S2 Fig — (a) BSS type includes a non-uniform density of synaptic weights (red line) and a uniform density of connections. (b) BSD type includes a uniform distribution of synaptic weights (red line) and non-uniformly distributed connections. (c) EP for large networks has been drawn in a graphic editor. (d) EP for small networks is prepared by the dynamic matrix. (e) (Top) Diagram of synaptic connections for STDP mechanisms. (Bottom) Examples of static rules of STDP. (f) (Top) Diagram of synaptic connections for the frequency dependent plasticity. (Bottom) Examples of rules for the frequency dependent plasticity. (TIF) [file pcbi.1005467.s004.tif]
