# Supplementary material for: ARACHNE: A neural-neuroglial network builder with remotely controlled parallel computing
Source: PLoS Comput Biol. 2017 Mar 31;13(3):e1005467. doi: 10.1371/journal.pcbi.1005467 (PMC5393895; doi:10.1371/journal.pcbi.1005467)
Supplement: S1 Code — (ZIP) [file pcbi.1005467.s005.zip › Arachne-master/Demo-version/InstructionBasic.docx]

Contents

[Preparing for the first launch of ARACHNE full version. 1](#_Toc466451424)

[Local machine side (GUI operating on MATLAB under Windows) 1](#_Toc466451425)

[ARACHNE with preinstalled cluster 1](#_Toc466451426)

[How to run a simulation on any remote cluster 2](#_Toc466451427)

[Preparing a HPC cluster (under Linux) 2](#_Toc466451428)

[Preparing local machine (under Windows) 2](#_Toc466451429)

[How to run simulation on local machine with OS Windows 3](#_Toc466451430)

# Demo version

The entire contents of Demo-version must be copied and installed anywhere on the local computer. To run GUI of ARACHNE the executable START_Arachne.exe must be launched. (See Manual.docx for details)

1. ## Demo version

This directory contains executables and directory "Core", to create and to compute networks on the cluster with a preinstalled kernel. These executables were compiled in MATLAB version 8.1.0.604 (R2013a). To run them on a local computer where this version of MATLAB is not installed (or the computer does not have any version of Matlab), user has to install corresponding version of MATLAB Runtime (R2013a). It is a free package that can be downloaded from the next webpage: <https://www.mathworks.com/products/compiler/mcr/>

The entire contents of Demo-version must be copied and installed anywhere on the local computer. To run GUI of ARACHNE the executable START_Arachne.exe must be activated. (See Manual.docx for details)

# Instruction

Download all files keeping the structure of the directory "Core" to your computer operating under OS Windows in any place.

Now you can launch START_ARACHNE.exe to execute the program.

1. Choose between 3 (or 2) different options: the option, 1, is for analysing of previous results, the option, 2, is for starting a new computation and the option, 3, is for continuing of previous computations
2. Launching the option 2 generates the window of GUI.
3. Using this GUI window user can a. check/modify the parameters of the “basic” networks model. See “Description parameters ARACHNE”. b. upload the file with another input parameters. c. save the new set of input parameters.
4. After modifying the parameters (if needed), then click OK, and the GUI generates the next window (External drivers for e-cells), in case, if Model(STDP)->EnableSTDP box checked. At this point, the user may specify the input image (the spatial patter of activation of e-cells) for the network storing. To do so, user has three options a) using a matrix generated by MATLAB (see the picture), where the number of element corresponds to the number of e-cell. Using a mouse one can click on any e-cell (technically it means that user add the depolarization current to the e-cell). This method of the external pattern generation is convenient for small networks b) drawing a black and white image with any graphic editor. It was designed for large networks. (See the example picture). The a black and white image can be downloaded. c) Black pixel in all cases indicates the depolarizing e-cell by 1 mV. The value of depolarization can be changed via the GUI (Model(STDP)->BlackValue).
5. After clicking OK the host send the file to the remote cluster located at 144.82.46.83 to start simulations.
6. When the simulation is completed, the cluster will generate results send the results back to the host and MATLAB will plot the results.
