# Supplementary material for: ARACHNE: A neural-neuroglial network builder with remotely controlled parallel computing
Source: PLoS Comput Biol. 2017 Mar 31;13(3):e1005467. doi: 10.1371/journal.pcbi.1005467 (PMC5393895; doi:10.1371/journal.pcbi.1005467)
Supplement: S1 Code — (ZIP) [file pcbi.1005467.s005.zip › Arachne-master/Instructions.docx]

Contents

[Preparing for the first launch 1](#_Toc467666512)

[Local machine side (under Windows). Full version 1](#_Toc467666513)

[Preparing HPC (OS Linux) cluster for running of ARACHNE 3](#_Toc467666514)

[How to run the simulation on your local computer without remote cluster. The host and remote parts are installed on the same computer. 4](#_Toc467666515)

# Preparing for the first launch

## Local machine side (under Windows). Full version

Download two directories ***host*** and ***worker*** from ***GITHUB*** repository, catalogue/Full-version

<https://github.com/LeonidSavtchenko/Arachne/tree/master/Full-version>

to your local computer:

1. Copy the directory “***<root>/host***” at any place of your local computer with Windows operating system and preinstalled MATLAB.
2. Copy the “***directory/worker***” on your cluster. ( See details “How to run a simulation on a remote cluster”)

To test the model ARACHNE, the cluster, located at 144.82.46.83, is already prepared. The following directory contains preinstalled software:

/home/reviewer/worker

Password: reviewer1

To start a simulation with the preinstalled configuration:

1. Go to the directory … “<root>/***host*** at your local computer
2. Launch the following Matlab script: “<root>/***host***/START_Arachne.m”.
3. Choose between 3 different options: option 1 is for analysing of previous results, option 2 is for starting a new computation and option 3 is for continuing of previous computations (See the next Fig).


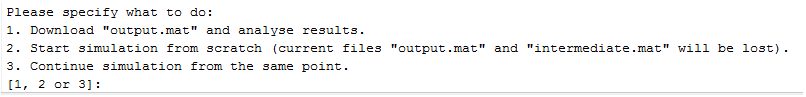


1. Option 2 generates the following window of GUI.


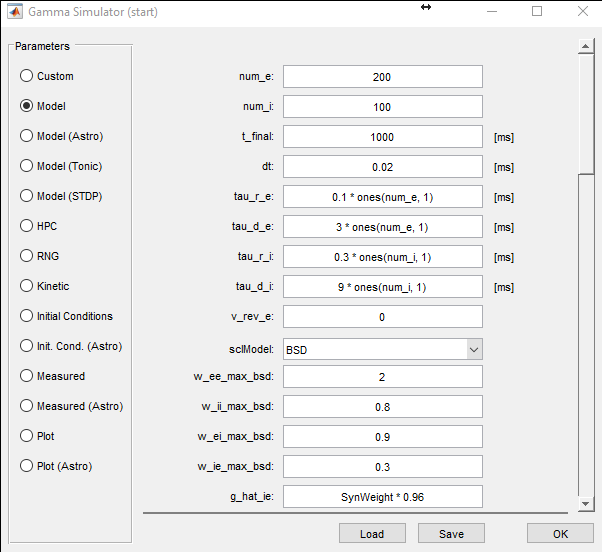


1. Using the GUI you can check/modify the parameters of the “***basic***” networks model. Three networks are already preinstalled. The description of parameters you can find in “***Description parameters ARACHNE***”.
2. After modifying the parameters, ARACHNE generates next stage where you can modify the external signal applied to networks. To do so, you have three options: 1) external condition generating the pattern with MATLAB code. It is very convenient for small networks and 2) distribution of the external signal in excitable cells can be set using a black and white picture drawing by any graphic editor. It was designed for large networks. 3) The patter of external signal can be downloaded as a picture. Black pixel in all cases depolarizes the cell membrane by 1 mV, but this setting can be changed


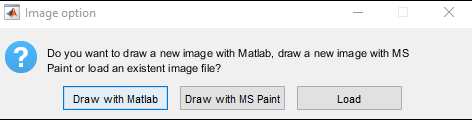


1. Option "Matlab" allows you to choose the each neuron from the matrix and give active current (activated) to any neuron by pressing a matrix element that changes colour.


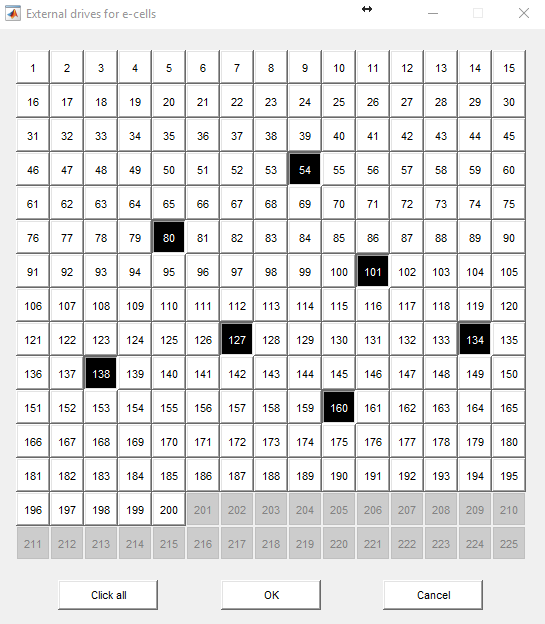


1. After that you can start the simulation on the remote cluster at 144.82.46.83.
2. When the simulation is completed, the cluster will generate results and your host computer will plot the results.

If you want to install ARACHNE on any others Linux/Windows clusters you need to do the following steps.

## Preparing HPC (OS Linux) cluster for running of ARACHNE

1. Before installation cluster part of ARACHNE, one need to be sure that “mpic++” compiler is present in the system path.
2. Download the folder “***worker***” with all its content from the GitHub/Full-version repository. This folder must be saved to the place shared between cluster nodes. For example, it can be saved in the directory “/home/<***username***>”.
3. Compile the application running script “<root>/***worker/build/lin_release.sh***.” If the compilation is successful, the file “***gs.exe***” must appear in the parental directory.

**Preparing your local computers (under Windows)**

1. Make sure that Matlab is installed on the local computer.
2. Download the folder “host” with its contents from GitHub repository to the local machine.
3. Open file on your host computer “<root>\***host\Core\scripts\win-lin\params.bat***” and adjust the following 4 variables: : “HEADNODEIP” – IP address of the cluster, “LOGIN” – Login name of your account of the cluster, “PASSWORD” – password of your account, “HEADNODEWORKERDIR” – the address of the location of “***gs.exe***” file on the cluster, to be consistent with your cluster.
4. Launch the following Matlab script: “<root>\***host\START_Arachne.m***”.

**When GUI appears, go to “HPC” Panel (see next picture) and adjust the following three elements:**


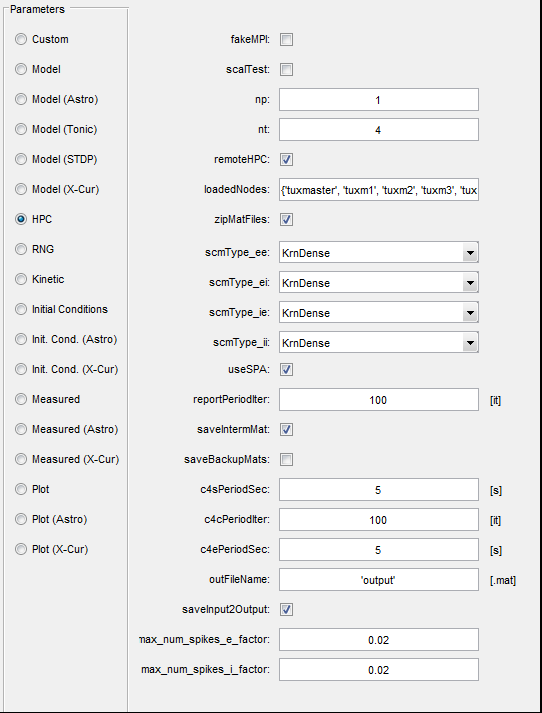


- Uncheck box fakeMPI
- Uncheck box scalTest
- Check box “remoteHPC” -- checked,
- edit box “nt” and”np” = the number of processor cores per one cluster node,
- check box “backgroundMode”.

1. After that you can click “OK” -- the simulation will be conducted on the remote cluster.
2. Description of all parameters you can find here “Panel: HPC”

#

#

# How to run the simulation on your local computer without remote cluster. The host and remote parts are installed on the same computer.

This option is an important when you need to start simulation with the host and remote parts are located on the same computer.

1. Make sure that you have Matlab installed on the local computer.
2. Make sure that Visual Studio Community installed on the local computer. This free IDE can be downloaded here:

<https://www.visualstudio.com/en-us/products/visual-studio-community-vs.aspx>

The “***Visual C++***” option must be checked during installation.

1. Download the folders “***host***” and “***worker***” keeping the structure and all of its content from GitHub repository to your local machine.
2. Open file ***“<root>\worker\build\vars.bat***” in any text editor and adjust the following three paths to be consistent with your machine: “VSDIR”, “MLDIR”, “GSDIR”. VSDIR is a director with Visual Studio, MLDIR is a director with Matlab, GSDIR is a directory with gs.exe
3. Run script “win_fakeMPI_release.bat” located in this directory. The file “gs_fakeMPI.exe” should appear in the parental directory after that.
4. Open file “<root>\host\Core\scripts\win-win\params.bat” and adjust “WORKERDIR” path.
5. Open file “<root>\host\Core\BasicParams\BasicParams.m” and set remoteHPC = false.
6. Launch the following Matlab script: “<root>\host\START_Arachne.m”.

**When GUI appears, go to “HPC” Panel and adjust the following three elements:**

- editbox “nt” = the number of cores in your processor,
- checkbox “backgroundMode” -- unchecked.

1. After that you can click “OK” -- the simulation will be conducted on your local machine instead of the cluster.
