# Supplementary material for: ARACHNE: A neural-neuroglial network builder with remotely controlled parallel computing
Source: PLoS Comput Biol. 2017 Mar 31;13(3):e1005467. doi: 10.1371/journal.pcbi.1005467 (PMC5393895; doi:10.1371/journal.pcbi.1005467)
Supplement: S1 Code — (ZIP) [file pcbi.1005467.s005.zip › Arachne-master/Manual.docx]

USER’S

MANUAL

ARACHNE

2016

# ARACHNE: A neural-neuroglial network builder with remotely controlled parallel computing.

**ARACHNE is a newly designed simulation environment in which neural network organization, optimization and execution take advantage of the pre-set, optimized parallel algorithms for remote computations and friendly user interface located on a local computer. With this strategy, even amateur is able to integrate the representative variety of biophysical mechanisms pertinent to nerve cells, within a single model in which the network complexity is limited only by the availability of remote computing resources, and not by the modelling kernel or its interface.**

This manual describes how to set up and run MPI+OMP version of tool ARACHNE. The general scenario of the simulation is as follows. A user works on a host computer using MATLAB installed under Windows/Android. MATLAB program generates input MAT-file and uploads it to remote HPC cluster operated under Linux/Windows. Then the cluster part starts a simulation without the communications with the host computer. The user’s computer connects to the remote process from time to time to a) know time of computation is and b) download intermediate results of the simulation (the results are shown in MATLAB Plots and save to the output file). When simulation completes, MATLAB host computer downloads the output MAT-file and visualises the final results.

Contents

[ARACHNE: A neural-neuroglial network builder with remotely controlled parallel computing. 2](#_Toc472298623)

[GETTING STARTED 5](#_Toc472298624)

[Preparing ARACHNE for the first launch 5](#_Toc472298625)

[Preparing HPC (OS Linux) cluster for running of ARACHNE 5](#_Toc472298626)

[Test ARACHNE using preinstalled cluster 5](#_Toc472298627)

[How to run the simulation on your local computer without remote cluster. 8](#_Toc472298628)

[Running the simulation 8](#_Toc472298629)

[Single experiment 8](#_Toc472298630)

[Scalability test 9](#_Toc472298631)

[Non-remote simulation modes 9](#_Toc472298632)

[ARACHNE parameters description 11](#_Toc472298633)

[The GUI Model Parameters 11](#_Toc472298634)

[Panel: Custom 11](#_Toc472298635)

[Panel: Model 11](#_Toc472298636)

[Synaptic connections localization models 13](#_Toc472298637)

[Panel: Model (Astro) 15](#_Toc472298638)

[Equations for astrocyte network 15](#_Toc472298639)

[Parameter of GUI for astrocyte networks. 15](#_Toc472298640)

[Panel: Model (Tonic) 16](#_Toc472298641)

[Panel: Model (STDP) 18](#_Toc472298642)

[Adopted mechanisms of synaptic plasticity 18](#_Toc472298643)

[Equation for the spike-time dependent plasticity (STDP) 18](#_Toc472298644)

[Equations for frequency-dependent plasticity 18](#_Toc472298645)

[Panel: Model (X-Cur) 19](#_Toc472298646)

[Panel: Model (MOD-Cur) 20](#_Toc472298647)

[Panel: HPC 21](#_Toc472298648)

[Panel: RNG (Random Number Generator) 25](#_Toc472298649)

[Panel: Kinetic (Kinetic of the basic currents) 25](#_Toc472298650)

[Panel: Initial Conditions 26](#_Toc472298651)

[Panel: Init. Cond. (Astro) 27](#_Toc472298652)

[Panel: Init. Cond. (X-Cur) 27](#_Toc472298653)

[Panel: Measured 27](#_Toc472298654)

[Panel: Measured (Astro) 27](#_Toc472298655)

[Panel: Measured (X/MOD-Cur) 28](#_Toc472298656)

[Panel: Plot 28](#_Toc472298657)

[Panel: Plot (Astro) 29](#_Toc472298658)

[Panel: Plot (X/MOD-Cur) 29](#_Toc472298659)

[How to change parameters on the remote cluster. 29](#_Toc472298660)

[(Example) How to changing deterministic external drives to E- and I-cells 29](#_Toc472298661)

[(Example) Changing system of equations 29](#_Toc472298662)

[(Example) Choosing and initialization of random number generators 30](#_Toc472298663)

[Model restrictions, memory requirements and performance 31](#_Toc472298664)

[Bit synaptic conductance matrices 31](#_Toc472298665)

[Memory allocated on a cluster node 31](#_Toc472298666)

[Limitations of the number of neurons caused by memory requirements 33](#_Toc472298667)

[Performance of ARACHNE 33](#_Toc472298668)

[Scalability curves for different types of matrices 34](#_Toc472298669)

[Structure of MATLAB host program 35](#_Toc472298670)

[Structure of HPC kernel 36](#_Toc472298671)

[Source code 36](#_Toc472298672)

[I/O directories 38](#_Toc472298673)

[Passing new parameters from host (MATLAB) to workers (C++) 38](#_Toc472298674)

[Passing a new scalar to an equation 38](#_Toc472298675)

[Passing a new vector to an element-wise operation 39](#_Toc472298676)

[The case of scattering of the vector 39](#_Toc472298677)

[The case of broadcasting of the vector 39](#_Toc472298678)

[Biophysics model 40](#_Toc472298679)

[Model equations for membrane potential dynamics 40](#_Toc472298680)

[Model equations for synaptic currents 40](#_Toc472298681)

[Network organization: Topology 40](#_Toc472298682)

[Network organization: Synaptic strengths and their distributions. 40](#_Toc472298683)

[Release probability distribution 41](#_Toc472298684)

[Setting the external input 41](#_Toc472298685)

[The network recall 42](#_Toc472298686)

[Basic set of parameters 42](#_Toc472298687)

[Equations for extracellular electric field 43](#_Toc472298688)

## GETTING STARTED

## Preparing ARACHNE for the first launch

The newest installation version currently available can be downloaded from https://github.com/LeonidSavtchenko/Arachne, which should be installed on the host and clusters computers.

Download two directories ***host*** and ***worker*** from ***GITHUB*** repository, catalogue/Full-version, which contains files for installation ARACHNE for any cluster operating under Linux/Windows and any host computer operating under Windows and with preinstalled MatLab.

The address of the catalog: <https://github.com/LeonidSavtchenko/Arachne> <Full-version>

1. Copy the directory “***<root>/host***” at any place of your local computer with Windows operating system and preinstalled MATLAB.
2. Copy the “***directory/worker***” on your cluster. (See details “How to run a simulation on a remote cluster”).

## Preparing HPC (OS Linux) cluster for running of ARACHNE

1. The cluster is prepared only once. Thereafter, only the host computer will be used. However, if the cluster will require substantial modification, the change in the cluster C++ code will be modified according the similar scenario.
2. Before installation the kernel of ARACHNE on the cluster, one need to be sure that “mpic++” (***Message Passing Interface***) compiler is present in the system path. The free version of mpic++ is here

<https://www.open-mpi.org/software/ompi/v2.0/>. Almost all modern clusters operating under Linux have this compiler in the system.

1. Download the folder “***worker***” with all its content from <https://github.com/LeonidSavtchenko/Arachne/><Full-version> repository. This folder must be saved to the place shared between cluster nodes. For example, it can be saved in the directory “/home/<***username***>”. To share files between a host computer and a remote cluster user can use free software “WinSCP” <https://winscp.net/eng/download.php>.
2. To compile the application, the following running script “<root>/***worker/build/lin_release.sh***.” must be activated typing in a command line ***sh. / lin_release.sh***. If the compilation is successful, the file “***gs.exe***” will appear in the parental directory.
3. The cluster is ready for the computation.

| Test ARACHNE using preinstalled cluster |
| --- |
| If you want to test ARACHNE before an installation to your own computers cluster, please use the preinstalled cluster located at 144.82.46.83. The following directory contains preinstalled software:  ***/home/reviewer/worker***  ***Login: reviewer***  ***Password: reviewer1***  To start a simulation with the cluster preinstalled configuration:   1. Go to the directory …<root>/***host*** at your local computer 2. Launch the following Matlab script: “…<root>/***host***/***START_Arachne.m***”. Make sure that Matlab is installed on the local computer. |

**Preparing “host” local computers (operating under Windows)**

1. System Requirements. ARACHNE supports MATLAB 7 and higher. The MATLAB code automatically adapts itself to the operating system on which it runs, eliminating the need for manual settings.
2. Download the folder “host” with its contents from <https://github.com/LeonidSavtchenko/Arachne/><Full-version> repository to the local machine operating under ***Windows***.
3. Open file on your host computer “<root>\***host\Code\scripts\win-lin\params.bat***” and adjust the following 4 variables: : “HEADNODEIP” – IP address of the cluster, “LOGIN” – Login name of your account of the cluster, “PASSWORD” – password of your account, “HEADNODEWORKERDIR” – the address of the location of “***gs.exe***” file on the cluster, to be consistent with your cluster.
4. ARACHNE host files are ready for preinstalled cluster.
5. Launch the following Matlab script: “<root>\***host\START_Arachne.m***”.
6.
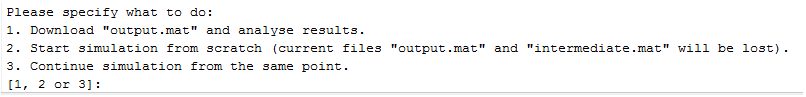

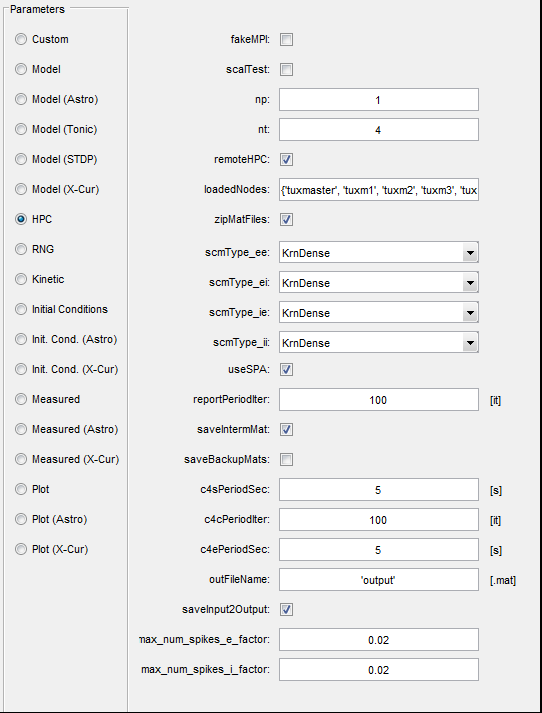
Choose between 3 different options: the option, 1, is for analysing of previous results, the option, 2, is for starting a new computation and the option, 3, is for continuing of previous computations (See the next Fig).
7. Launching the option 2 generates the following window of GUI.
8. ***Very important***. Uncheck box fakeMPI, Uncheck box scalTest, Check box “remoteHPC” -- checked, edit box “nt” and”np” = the number of processor cores per one cluster node, check box “backgroundMode”.
9. Using this GUI window user can
   1. check/modify the parameters of the “***basic***” networks model. See “***Description parameters ARACHNE***”.
   2. upload the file with another input parameters.
   3. save the new set of input parameters.
10. After modifying the parameters (if needed), then click OK, and the GUI generates the next window (***External drivers for e-cells***), in case, if ***Model(STDP)->EnableSTDP*** box checked. At this point, the user may specify the input image (the spatial patter of activation of e-cells) for the network storing. To do so, user has three options (see picture):


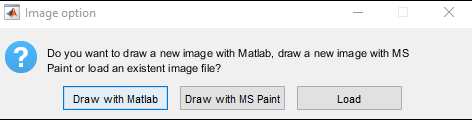


1. using a matrix generated by MATLAB (see the picture), where the number of element corresponds to the number of e-cell. Using a mouse one can click on any e-cell (technically it means that user add the depolarization current to the e-cell). This method of the external pattern generation is convenient for small networks


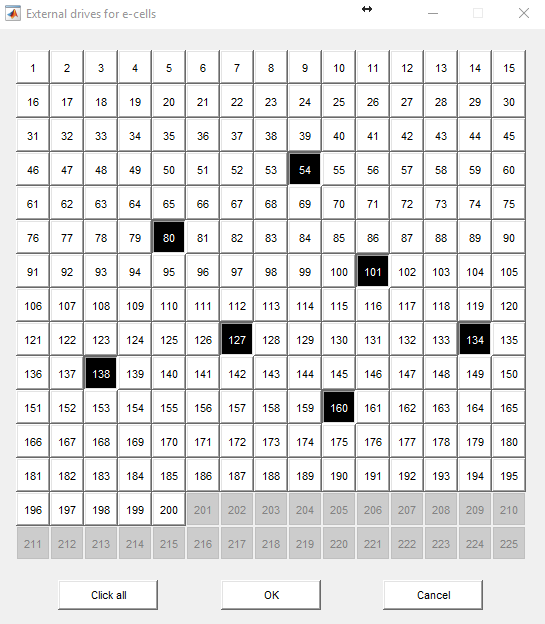


1. drawing a black and white image with any graphic editor. It was designed for large networks. (See the example picture).


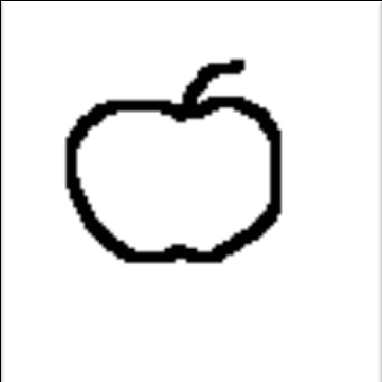


3) The a black and white image can be downloaded.

Black pixel in all cases indicates the depolarizing ***e***-cell by 1 mV. The value of depolarization can be changed via the GUI (***Model(STDP)->BlackValue)***.

1. After clicking OK the host send the file to the remote cluster located at 144.82.46.83 to start simulations.
2. When the simulation is completed, the cluster will generate results send the results back to the host and MATLAB will plot the results.

### How to run the simulation on your local computer without remote cluster.

The host and remote parts are installed on the same computer.

This option is an important when you need to start simulation with the host and remote parts are located on the same computer.

1. Make sure that you have Matlab installed on the local computer.
2. Make sure that Visual Studio Community installed on the local computer. This free IDE can be downloaded here:

<https://www.visualstudio.com/en-us/products/visual-studio-community-vs.aspx>

**Important.** The “***Visual C++***” option must be checked during installation.

1. Download the folders “***host***” and “***worker***” keeping the structure and all of its content from GitHub repository to your local machine.
2. Open file ***“<root>\worker\build\vars.bat***” in any text editor and adjust the following three paths to be consistent with your machine: “VSDIR”, “MLDIR”, “GSDIR”. VSDIR is a director with Visual Studio, MLDIR is a director with Matlab, GSDIR is a directory with gs.exe
3. Run script “win_fakeMPI_release.bat” located in this directory. The file “gs_fakeMPI.exe” should appear in the parental directory after that.
4. Open file “<root>\host\Code\scripts\win-win\params.bat” and adjust the following 2 paths: “MATLABHOSTDIR”, “WORKERDIR”.
5. Open file “<root>\host\Code\BasicParams\BasicParams.m” and set remoteHPC = false.
6. Launch the following Matlab script: “<root>\host\START_Arachne.m”.

**When GUI appears, go to “HPC” Panel and adjust the following three elements:**

- editbox “nt” = the number of cores in your processor,
- checkbox “backgroundMode” -- unchecked.

1. After that you can click “OK” -- the simulation will be conducted on your local machine instead of the cluster.

# Running the simulation

## Single experiment

1. Wake up all nodes of the cluster. (Alternatively, you can run simulation on the master node only.)
2. Open file “***host\START_Arachne.m***” in MATLAB and press F5.
3. Open Panel Hpc specify *remoteHPC = true*.

Specify number of processes “np” you are going to run on the cluster. (The number should not exceed the total number of processors on available cluster nodes.) Specify number of threads per process “nt”. (The number should not exceed the number of cores of a processor.)

If everything is OK, you will see messages in MATLAB console reporting about:

- Uploading of input MAT-file to the head node of the cluster.
- Progress of simulation.
- Downloading of output MAT-file from the head node.

Then windows with graphical representation of the results must appear.

If you need to stop the simulation, click on Command Window in MATLAB and press Ctrl + Break. This stops the client (local computer) part of simulation, but not the server performance. In order to stop server operations, one needs to run the first or the second script from the following:

1. “SCRIPT_TerminateBackgroundProcess.m”.
2. “SCRIPT_KillBackgroundProcess.m”.

## Scalability test

1.
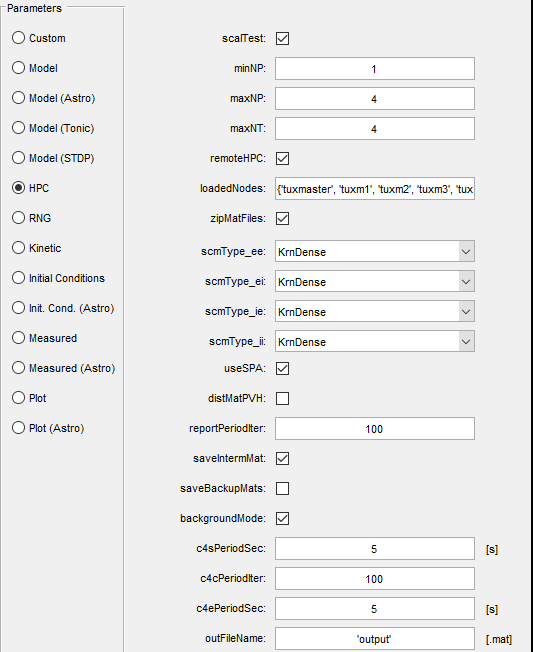
In GUI HPC specify *scalTest = true*.

Specify maximum number of processes “np” and maximum number of threads per process “nt” you are going to use in the test.

1. Specify the minimum number of processes “minNP” taking into account that a very large number of neurons and too small number of processes may cause memory deficit (see the paragraph below). On the other hand, make sure that the number of neurons is large enough to show the real scalability.

Here are some examples:

1. minNP = 1; np = 9; nt = 4; num_e = 200; num_i = 100; t_final = 500; radius_e = 250; radius_i = 200; v = 0.1; scmType_XY = KrnDense; distMatPVH = false;

BAD — not enough neurons to show real scalability;

1. minNP = 1; np = 9; nt = 4; num_e = 8600; num_i = 4300; t_final = 0.2; radius_e = 250; radius_i = 200; v = 0.1; scmType_XY = KrnDense; distMatPVH = false;

BAD — memory deficit happens for number of processes less than 5 (the amount of required physical memory per cluster node exceeds 1700 MB);

1. minNP = 8; np = 9; nt = 4; num_e = 8600; num_i = 4300; t_final = 0.2; radius_e = 250; radius_i = 200; v = 0.1; scmType_XY = KrnDense; distMatPVH = false;

GOOD.

If everything is OK, you will see messages in MATLAB console reporting that simulation is run for number of processes equal *minNP, minNP + 1, …, np* and number of threads per process equal *1, 2, …, nt* in sequence.

At the end the window must appear showing set of scalability curves. Each curve y(x) corresponds to specific number of threads and is defined by “x” being number of processes and “y” being inverse time of execution.

# Non-remote simulation modes

Besides the remote simulation mode described above, the simulator can be run in the following two modes:

1. Running from MATLAB on local machine under Windows on the same machine.
2. Running from MATLAB on head node of Linux cluster on the same cluster.

These two modes were developed mostly for debugging and testing purposes. In order to use the first mode, you need to adjust variables defined in the script “host\scripts\win-win\params.bat.” Usage of the second mode requires adjustment of variables defined in “host\scripts\lin-lin\params.sh.” The parameter “remoteHPC” defined in GUI HPC panel should be specified as “false” in both cases.

The first mode needs compilation of HPC kernel under Windows. The compilation requires the following software installed:

1. Microsoft MPI available for free by the following reference:

<https://www.microsoft.com/en-us/download/details.aspx?id=52981>

1. Microsoft Visual Studio Community 2015 or other C++ compiler. Notice that compilation with other C++ compiler may require changes in the code of HPC kernel because each compiler has its own specific requirements to the code.
2. MATLAB R2013a. Usage of some other version may require changes in code of HPC kernel.

MATLAB is necessary because it provides compile-time libraries and run-time libraries used for MAT-file read/write staff. The directory with MATLAB libraries must be added to the system path variable. You can access the variable as following:

1. Go to Windows Explorer -> My Computer -> Properties -> Advanced -> Environment Variables.
2. Select the variable with name “Path” and click “Edit” button.
3. Add “C:\Program Files\MATLAB\R2013a\bin\win64” to the end of the string. (Your addition to the string will be other if you installed MATLAB to some non-default location.)

Alternatively, you can copy stuff of the directory “C:\Program Files\MATLAB\R2013a\bin\win64” to the directory containing ARACHNE executable “gs.exe.”

Compilation of HPC kernel under Windows is performed with one of the following scripts located in the directory “worker”:

1. build_Windows_DEBUG.bat.
2. build_Windows_RELEASE.bat.

# ARACHNE parameters description

## The GUI Model Parameters

ARACHNE generates the table of parameters


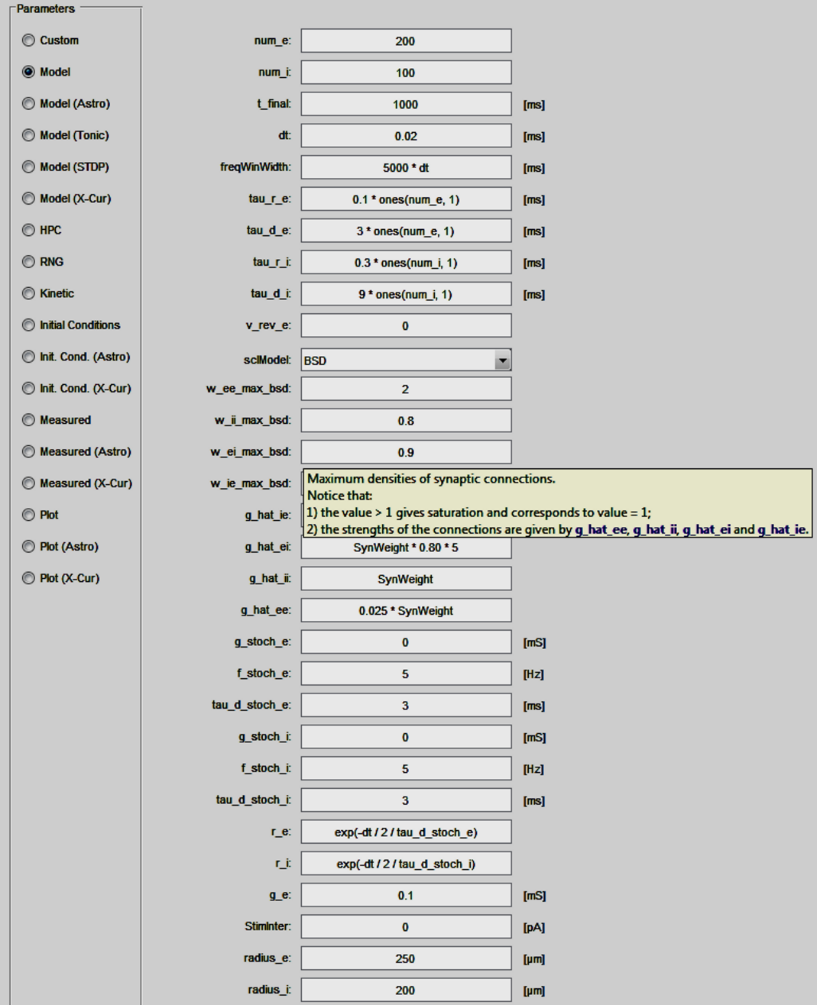


## Panel: Custom

Parameter: ***customCode*** Description: Text areas for custom m-code where user can define any additional variables accessible from the expressions in edit boxes.

**The basic model includes three networks. First network is a network of E-cells (excitatory networks), the second network is network of I-cells (Inhibitory cells) and a-cell (astrocyte networks)**

## Panel: Model

Parameter: ***num_e*** Description: Number of E-cells

Parameter: ***num_i*** Description: Number of I-cells

Parameter: ***t_final*** Description: Time of simulations

Parameter: ***dt*** Description: Time step used in the midpoint method

Parameter: ***freqWinWidth*** Description: Width of time window used for calculation of spikes frequencies in the following two cases: 1) calculation of overall i-network frequency if ***dynamicGTonicGABA*** is checked; 2) calculation of particular neurons frequencies if ***enableSTDP*** is checked.

Parameter: ***tau_r_e*** Description: Rise time constant of associated with E-cells synapses

Parameter: ***tau_d_e*** Description: Decay time constant associated with E-cells synapses

Parameter: ***tau_r_i*** Description: Rise time constant associated with I-cells synapses

Parameter: ***tau_d_i*** Description: Decay time constant associated with I-cells synapses

Parameter: ***v_rev_e*** Description: Synaptic reversal potential of e-cell

(See details in ***Synaptic connections localization models***)

Parameter: ***sclModel*** Description: Synaptic connections localization model BSS: bell-shaped strength and uniform density of connections BSD: uniform strength and bell-shaped density of connections


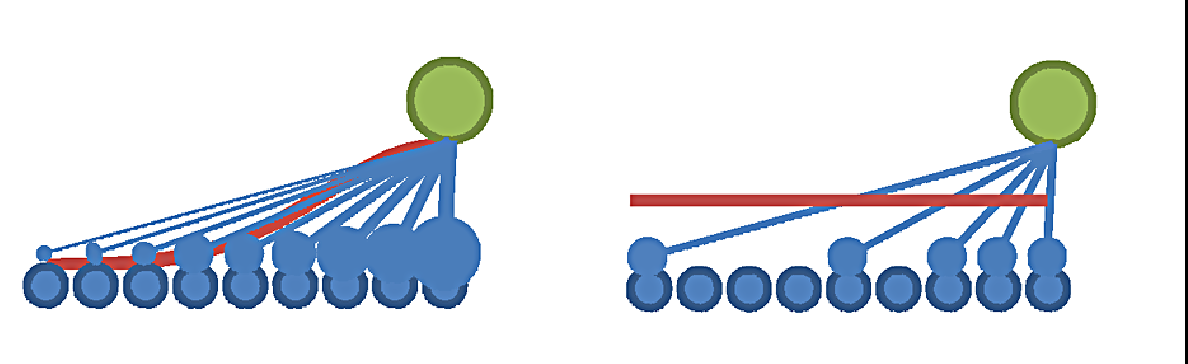


BSD

BSS

Figure. Two types of synaptic distribution.

Parameter: ***w_ee_max_bss*** Description: Maximum strengths of ee-synaptic connections (the density of synaptic connections is highest possible)

Parameter: ***w_ii_max_bss*** Description: Maximum strengths of ii-synaptic connections (the density of synaptic connections is highest possible)

Parameter: ***w_ei_max_bss*** Description: Maximum strengths of ei-synaptic connections (the density of synaptic connections is highest possible)

Parameter: ***w_ie_max_bss*** Description: Maximum strengths of ie-synaptic connections (the density of synaptic connections is highest possible)

Parameter: ***w_ee_max_bsd*** Description: Maximum densities of ee-synaptic connections. Notice that: 1) the value > 1 gives saturation and corresponds to value = 1; 2) the strengths of the connections are given by g_hat_ee , g_hat_ii , g_hat_ei and g_hat_ie .

Parameter: ***w_ii_max_bsd*** Description: Maximum densities of ii-synaptic connections. Notice that: 1) the value > 1 gives saturation and corresponds to value = 1; 2) the strengths of the connections are given by g_hat_ee , g_hat_ii , g_hat_ei and g_hat_ie .

Parameter: ***w_ei_max_bsd*** Description: Maximum densities of ei-synaptic connections. Notice that: 1) the value > 1 gives saturation and corresponds to value = 1; 2) the strengths of the connections are given by g_hat_ee , g_hat_ii , g_hat_ei and g_hat_ie .

Parameter: ***w_ie_max_bsd*** Description: Maximum densities of ie-synaptic connections. Notice that: 1) the value > 1 gives saturation and corresponds to value = 1; 2) the strengths of the connections are given by g_hat_ee , g_hat_ii , g_hat_ei and g_hat_ie .

Parameter: ***g_hat_ie*** Description: Conductance of synaptic ie-connections

Parameter: ***g_hat_ei*** Description: Conductance of ei-synaptic connections

Parameter: ***g_hat_ii*** Description: Conductance of ii-synaptic connections

Parameter: ***g_hat_ee*** Description: Conductance of ee-synaptic connections

Parameter: ***g_stoch_e*** Description: Maximum conductance of stochastic excitatory input pulses (a synaptic noises coming from outside these networks).

Parameter: ***f_stoch_e*** Description: Frequency of Poisson excitatory signal (a synaptic noise mean frequency)

Parameter: ***tau_d_stoch_e*** Description: Decay time of single excitatory synaptic noise

Parameter: ***g_stoch_i*** Description: Maximum conductance of inhibitory input pulses (a synaptic noises coming from outside these networks).

Parameter: ***f_stoch_i*** Description: Frequency of Poisson inhibitory signal (a synaptic noise mean frequency)

Parameter: ***tau_d_stoch_i*** Description: Decay time of a single inhibitory synaptic noise

Parameter: ***r_e*** Description: Synaptic delay of e-synapses

Parameter: ***r_i*** Description: Synaptic delay of i-synapses

Parameter: ***g_e*** Description: Parameter connection between i and e. The scaling of synaptic conductance between e and i neurons (ge* g_hat_ei). Dimensionless.

Parameter: ***StimInter*** Description: Constant depolarization of i-neurons in pA

Parameter: ***radius***_e Description: Radius of e-network

Parameter: ***radius***_i Description: Radius of i-network

These two variables specify the radii of ring networks in micrometres. Variable “radius_e” determines radius of e-network and variable “radius_i” specifies radius of i-network. The numbering of neurons in ring networks guarantees that the distance between the first neuron of e-network and the first neuron of i-network is minimal (see picture 1).


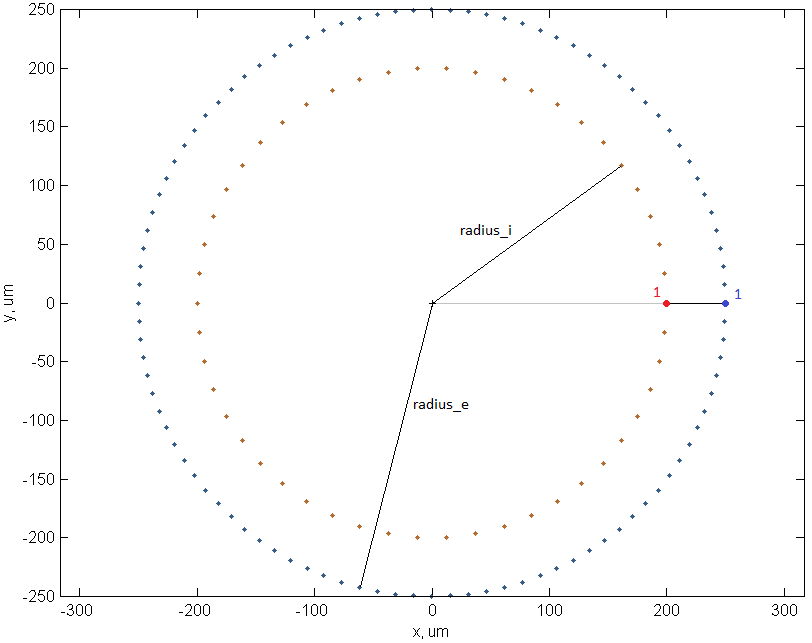


Picture 1.

Parameter: ***v*** Description: Rate of signal propagation between neurons. This parameter defines the delay of signal between neurons. The parameter specifies the rate of signal propagation in micrometres per millisecond.

Parameter: ***releaseProb***_ee Description: Release probability within e-network

Parameter: ***releaseProb***_ii Description: Release probability within i-network

Parameter: ***releaseProb***_ei Description: Release probability from e-network to i-network

Parameter: ***releaseProb***_ie Description: Release probability from i-network to e-network

Parameter: ***sigmaDivisor***_ee Description: Spatial variability of ee synapses

Parameter: ***sigmaDivisor***_ei Description: Spatial variability of ei synapses

Parameter: ***sigmaDivisor***_ie Description: Spatial variability of ie synapses

Parameter: ***sigmaDivisor***_ii Description: Spatial variability of ii synapses

## Synaptic connections localization models

There are two models of synaptic connections localization. In both model the normal distribution of one synaptic connection parameter is used:

.

The first model consists in bell-shaped strength and uniform density of connections. Synaptic conductance matrices of this model are presented at the picture 2 with following set of parameters:

num_e = 1000; num_i = 500;

w_ee_max = 1.3; w_ii_max = 0.35; w_ei_max = 0.5; w_ie_max = 0.15;

sigma_ee = 100; sigma_ei = 75; sigma_ie = 75; sigma_ii = 50.


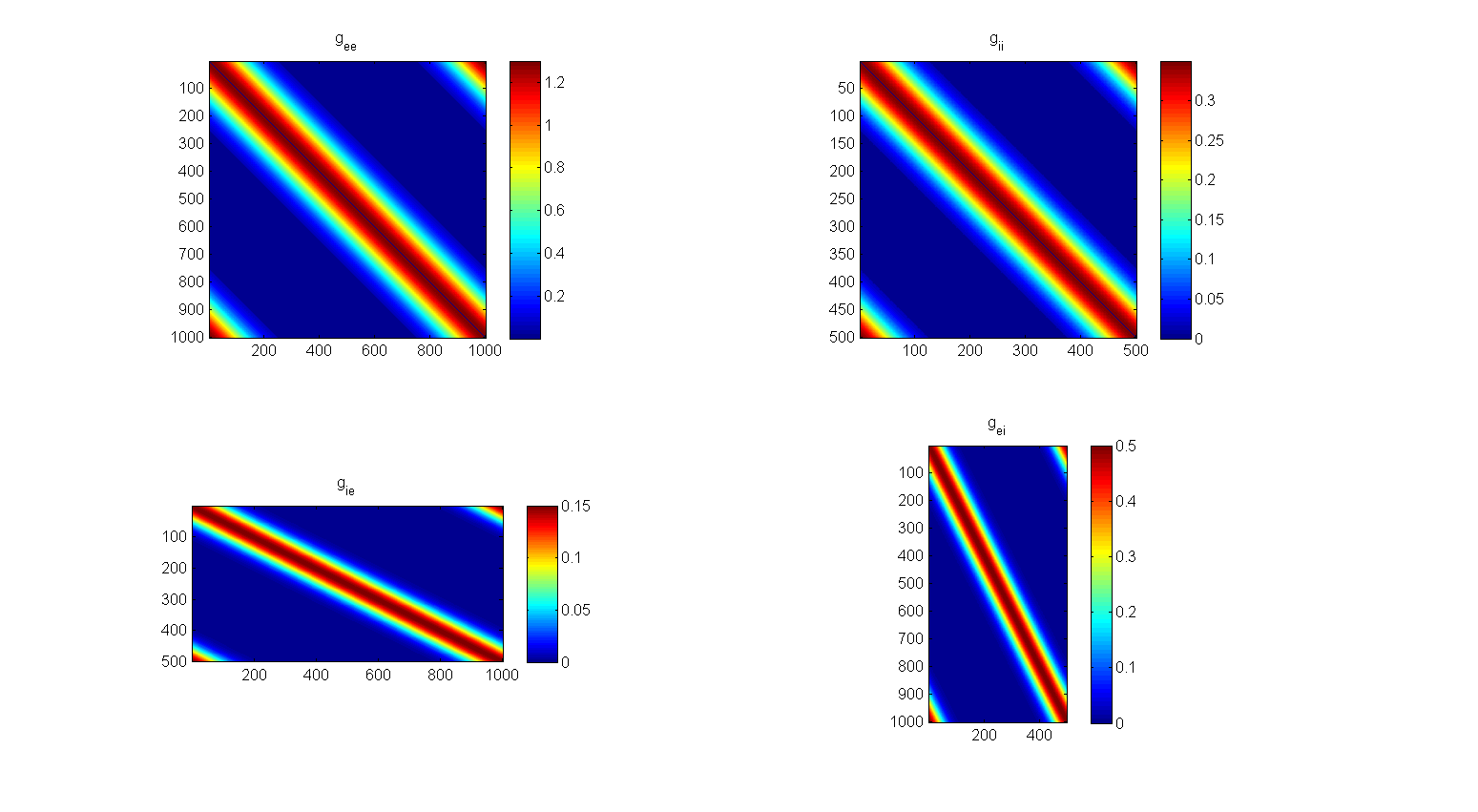


Picture 2

The second model consists in uniform strength and bell-shaped density of connections. If in this model the parameter “w_XY_max” is more than unit, the density of connections between neurons becomes saturated (compare matrices “g_ee” and “g_ie” at the picture 1). Synaptic conductance matrices of this model are presented at the picture 3 with following set of parameters:

num_e = 1000; num_i = 500;

g_hat_ie = 1.2; g_hat_ei = 1.0; g_hat_ii = 1.0; g_hat_ee = 1.0;

w_ee_max = 2; w_ii_max = 0.8; w_ei_max = 0.9; w_ie_max = 0.3;

sigma_ee = 100; sigma_ei = 75; sigma_ie = 75; sigma_ii = 50.


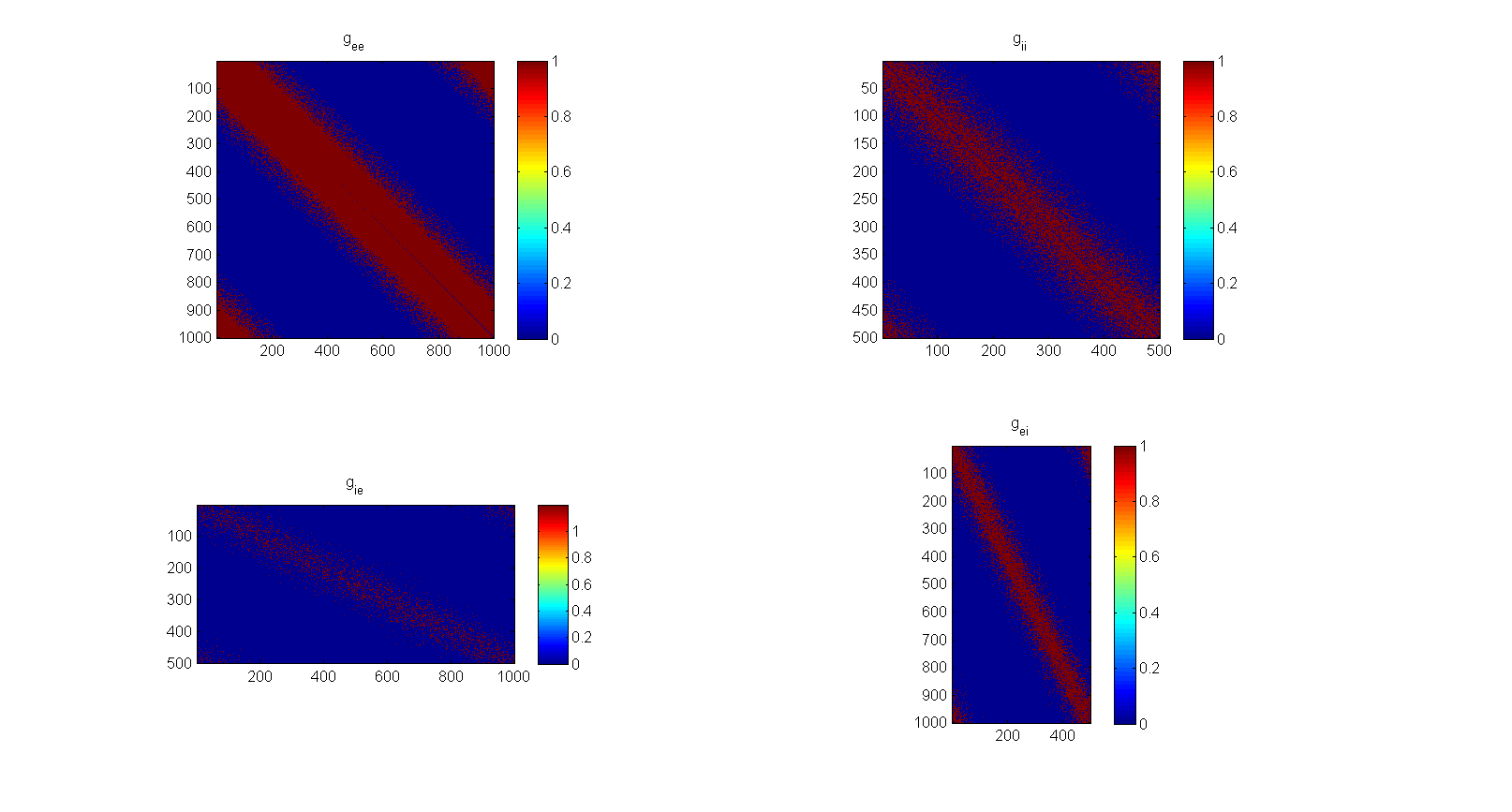


Picture 3

In this model of localization of synaptic connections the value w_XY_max >= 1 gives saturated density of connections.

In both models the elements on principal diagonals of intranetwork matrices (“g_ee” and “g_ii”) are always equal to zero to avoid influence of neuron on itself.

## Panel: Model (Astro)

### Equations for astrocyte network

In the model, the astrocytic intracellular calcium dynamic is described by the following equation :

where is the flux through SERCA pumps, is the leak from the endoplasmic reticulum into the cytosol, is the calcium fluxes through the gap junctions generated by the nearest left and right astrocytes, is a calcium voltage-dependent current from the intracellular store, where *q* is the fraction of active IP3 receptors and *gs* is the conductance of the gap junction between astrocytes.

The parameters of the intracellular stores are defined by the following equations:

Given that IP3 degrades with a time constant of *τp*, an equation for cytosolic IP3 is needed, specified as

,

where [*y*] is concentration of neurotransmitter release from the synapse, *rip*3 [mM s-1]is the rate of IP3 release.

The dimension and values of parameters see in the section *“Basic set of parameters”*

Local concentration of neurotransmitters depends on the spike generation by nearest neurons and the uptakes by the pump. The dynamics is described by the following equation

Where *τ*spike is the time scale of spike, and *tp* is a time course of uptake.

The calcium concentration of astrocyte [*Ca*]*astro* influences the release probability of a nearby neuronal synapse according to the equation

where *p* is a release probability of the synapse from e-neuron to the i-neurons. Astrocytes interact through the gap junctions.

All of this parameters user can modify using the GUI.

### Parameter of GUI for astrocyte networks.

Parameter: ***enableAstro*** Description: Whether to enable the astrocyte network

Parameter: ***astroTimeScale*** Description: Astrocytes evolution time scale divisor. This time constant defines how often the astrocyte network dynamics updated.

Parameter: ***v1*** Description: Parameter of astrocyte network

Parameter: ***v2*** Description: Parameter of astrocyte network

Parameter: ***v3*** Description: Parameter of astrocyte network

Parameter: ***d1*** Description: Parameter of astrocyte network

Parameter: ***d2*** Description: Parameter of astrocyte network

Parameter: ***d3*** Description: Parameter of astrocyte network


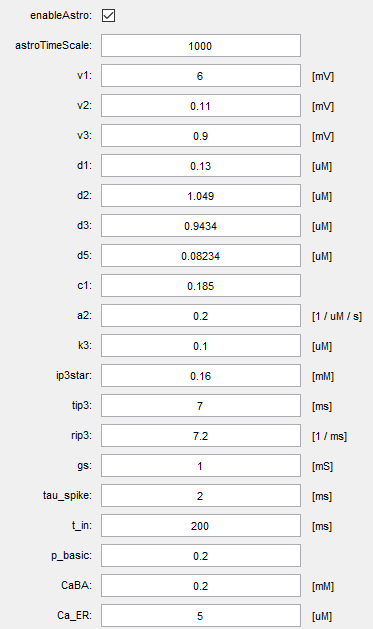
 Parameter: d5 Description: Parameter of astrocyte network

Parameter: c1 Description: Parameter of astrocyte network

Parameter: a2 Description: Parameter of astrocyte network

Parameter: k3 Description: Parameter of astrocyte network

Parameter: ip3star Description: IP3 basic concentration

Parameter: tip3 Description: IP3 time constant

Parameter: rip3 Description: Parameter of astrocyte network

Parameter: gs Description: Parameter of astrocyte network

Parameter: tau_spike Description: Parameter of astrocyte network

Parameter: t_in Description: Parameter of astrocyte network

Parameter: p_basic Description: Release probability

Parameter: CaBA Description: Resting Ca concentration

Parameter: Ca_ER Description: Ca concentration inside inside endoplasmic reticulum

## Panel: Model (Tonic)

#### Equation for tonic currents

Experimental data revealed that the dynamics of the networks depend on the concentration of extracellular neurotransmitter. The neurotransmitter activates the extrasynaptic receptors which generate the tonic currents. To take into account this mechanism, the neurons in the model include the following non-specific tonic current, *Itonic* Eq.:

where *Gtonic* is a tonic conductance, *Vtonic* is a reverse potential of tonic current and *V* is the membrane potential. The non-specificity means that the user can set either excitatory or inhibitory tonic current.

Model affords two options for the tonic conductance: constant conductance and network activity dependent conductance.

In the brain, the extracellular neurotransmitter concentration varies depending on the frequency of synaptic release and the ion pumps activity. In turn, the extracellular modification of the neurotransmitter concentration affects the extrasynaptic receptor activations and thus modifies neuronal excitability and thus the frequency of synaptic release. To take into account the feedback between neurotransmitter concentration and network activity, a new biophysical mechanism is added to the model, which relates the tonic current conductance to the firing frequency of i-neurons.

Mathematically, the dynamics of *Gtonic* is described by the following equations.

##### The first model

, a

The critical dynamic variable of the equation is the i-neurons network firing frequency Eq.:

The variable, *fm* is the average frequency of the interneuron network calculated as a sum of all APs generated by all i-neurons within the time frame of **.

Other parameters are: *Ni* is the number of neurons in the network, *Af* is the change of the tonic conductance as a single interneuron generates a single AP during time *T*, *tp* is the rate of neurotransmitter uptake, *Gb* is the reverse concentration of neurotransmitter uptake, *fb* is the basic frequency of network at resting state, the parameter *dt* is the time delay between the release of neurotransmitter and the activation of extrasynaptic receptors.

The main assumption of the equation is that the tonic conductance is linearity depended on the neurotransmitter.

With this biophysical mechanism, the model enables a feedback between the tonic current conductance and the network dynamics, as we have shown earlier using simpler network configurations.

##### The second model

, b

where

with [*GABA*] being concentration, *T* being calculation time, Δ*t* being bin time of calculation of AP number, *ni* being the number of spikes in *i*-network within Δ*t* interval.


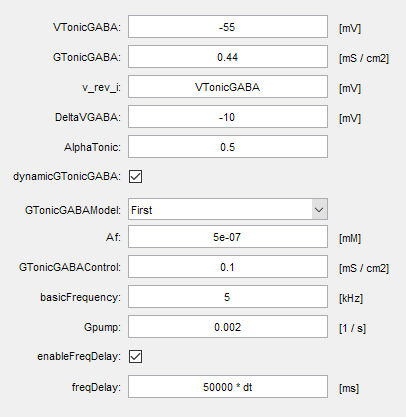
 Parameter: ***VTonicGABA*** Description: Reverse potential of tonic current

Parameter: ***GTonicGABA*** Description: Conductance of tonic current. If ***dynamicGTonicGABA*** is checked, then this parameter gives the initial condition.

Parameter: ***v_rev_i*** Description: Reverse potential of synaptic GABA

Parameter: ***DeltaVGABA*** Description: Difference between reverse potential of i- and e- neurons

Parameter: ***AlphaTonic*** Description: Ratio of tonic conductance between e- and i-neurons.

Parameter: ***dynamicGTonicGABA*** Description: The type of GTonicGABA model. ***Unchecked***: no time dependency, it's a constant equal ***GTonicGABA***. ***Checked***: the function of time defined by an ODE dependent on ***GTonicGABAModel***.

Parameter: ***GTonicGABAModel*** Description: The type of GTonicGABA model that can be either ***First*** or ***Second*** for the equations a and b respectively.

Parameters of the ***First*** model:

Parameter: ***Af*** Description: Rate of GABA concentration on frequency

Parameter: ***GTonicGABAControl*** Description: Rating tonic conductance

Parameter: ***basicFrequency*** Description: The basic frequency which keeps constant of Gtonic conductance

Parameters of the ***Second*** model:

Parameter: ***G0*** Description: Shift parameter

Parameter: ***Rate*** Description: Scale parameter

Parameter: ***V*** Description: Effective volume

Parameter: ***N*** Description: Number of molecules release during of single AP

Parameter: ***Na*** Description: Avogadro number

Parameter: ***GABAb*** Description: Rating tonic concentration

Parameter: ***Gpump*** Description: Rate of GABA pumping

Parameter: ***enableFreqDelay*** Description: Whether to delay the frequency parameter.

Parameter: ***freqDelay*** Description: The delay for i-network frequency. Time delay between frequency modification and conductance modification

## Panel: Model (STDP)

### Adopted mechanisms of synaptic plasticity

To simulate the processes of memorization, the model includes three common mechanisms of synaptic plasticity: (a) spike-timing dependent plasticity (STDP), (b) frequency-dependent plasticity, and (c) synaptic plasticity due to activation of a neighbouring astrocyte.

A distinct mechanism of synaptic plasticity implemented in the model deals with modifications of *p* due to calcium-dependent astrocyte activity. The model is set so that when the calcium level inside an astrocyte exceeds a chosen threshold, the astrocyte 'releases' signalling molecules (such as ATP or adenosine) that affect *p* at neighbouring ***ei***‑synapses in tissue volume. Model provides an opportunity to switch off the astrocyte network thus keeping *p* unchanged throughout.

### Equation for the spike-time dependent plasticity (STDP)

To fit a wide range of STDP rules into a single instruction, the following formula was introduced:

where *W* and *dW* are the synaptic weigh and its modification, *t* is a time difference between the presynaptic and postsynaptic APs. Parameters *A+, A-, Ac* and *As* define the amplitude of *dW*. The model is designed so that for a given synapse there is only one non-zero amplitude parameter. This method allows the type of plasticity for a given synaptic connection to be chosen by using only parameters stored on the host computer and leaving the compiled computational code on the cluster unchanged.

Parameters *tc, ts, S*1*, S*2*, Cp* and *Sp* characterise the time course of STDP and *ta* is nondimensional scaling parameter.

### Equations for frequency-dependent plasticity

The model provides a frequency dependent regulation of synaptic strength. The linear approximation between the synaptic strength *W* and the presynaptic and the postsynaptic frequencies *ωpre* and *ωpost* is observed in different areas of the brain and described by a simple equation Eq.

where Δ*W* is the synaptic strength modification, which occurs in time Δ*t*; *α* and *β* set the synaptic strength sensitivity to the presynaptic and postsynaptic frequencies, respectively; and  is a characteristic time of synaptic modification.

**The table of parameters for all type of synaptic plastisity**


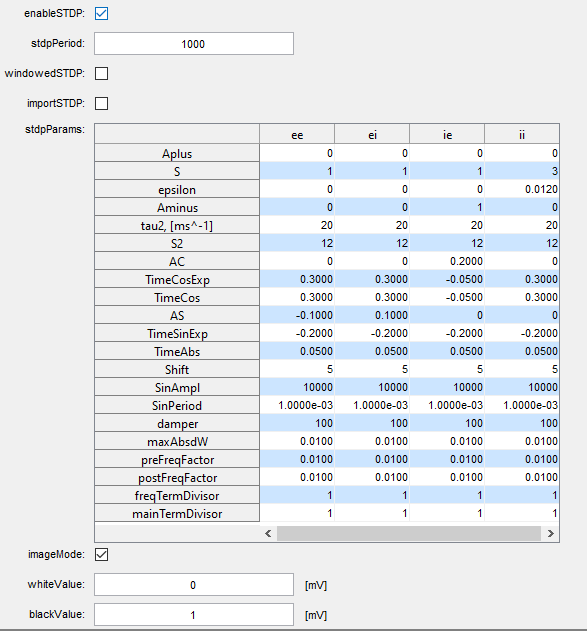
 Parameter: ***enableSTDP*** Description: Whether to correct matrices of synaptic conductance according to Hebbian theory

Parameter: ***stdpPeriod*** Description: How often Hebbian correction is done (in iterations)

Parameter: ***windowedSTDP*** Description: Whether smooth window (Epsilon) is applied in the analyzed STDP interval

Parameter: ***importSTDP*** Description: Whether STDP models are imported from text files

Parameter: ***stdpParams*** Description: Parameters of spike-timing-dependant plasticity (Hebbian correction) for each matrix of synaptic conductance

***See formula 5 for description***

Parameter: ***stdpFile_ee*** Description: Name of the file to import STDP model from (ee-interaction)

Parameter: ***stdpFile_ei*** Description: Name of the file to import STDP model from (ei-interaction)

Parameter: ***stdpFile_ii*** Description: Name of the file to import STDP model from (ii-interaction)

Parameter: ***stdpFile_ie*** Description: Name of the file to import STDP model from (ie-interaction)

Parameter: ***stdpCommonParams*** Description: Common parameters of spike-timing-dependant plasticity

Parameter: ***imageMode*** Description: Whether to apply image drive to e-cells

Parameter: ***whiteValue*** Description: The value of the drive corresponding to white cells in the image

Parameter: ***blackValue*** Description: The value of the drive corresponding to black cells in the image

## Panel: Model (X-Cur)

The basic state of ARACHNE includes a GUI table allowing to introduce any non-specific current (tonic or phasic) *Iextra*. The latter is given by expression

with the maximum conductance *gextra*, the activation variable *ae*, the inactivation variable *be* and the reversal potential *Vre*. The powers *pj* and *qj* represent the number of gating states of the ionic channels and are integers between 0 and 4 inclusively. Variables *ae* and *be* are assumed to obey the first-order ordinary differential equation

The steady state values of *x∞*(V) are sigmoid functions of the membrane potential

, where *Vx* and *sx* represent the threshold and the slope of the steady state curve, respectively. The rate coefficient *kx*(V) has the following voltage dependency

, where *tx* is a time constant.

The software provides a possibility to modify the kinetics using the GUI. In non-excitable astrocytes, the membrane potential *V* is set to a constant, whereas the main dynamic variable is the intracellular calcium concentration.

***The parameters can be modify using GUI.***


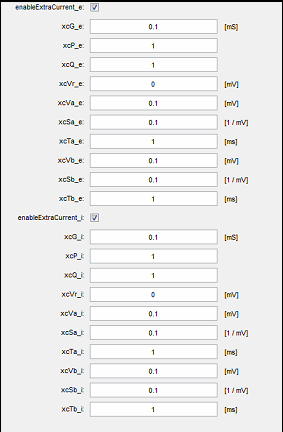


Parameter: ***enableExtraCurrent***_e Description: Whether to enable the extra current for e-neurons

Parameter: ***xcG_e*** Description: The maximum conductance

Parameter: ***xcP_e*** Description: The multiplicity of gating elements in the ionic channels

Parameter: ***xcQ_e*** Description: The multiplicity of gating elements in the ionic channels

Parameter: ***xcVr_e*** Description: The reversal potential of this current

Parameter: ***xcVa_e*** Description: The threshold of the steady state curve in the a ODE

Parameter: ***xcSa_e*** Description: The slope of the steady state curve in the a ODE

Parameter: ***xcTa_e*** Description: The time constant in the a ODE

Parameter: ***xcVb_e*** Description: The threshold of the steady state curve in the b ODE

Parameter: ***xcSb_e*** Description: The slope of the steady state curve in the b ODE

Parameter: ***xcTb_e*** Description: The time constant in the b ODE

Parameter: ***enableExtraCurrent_i*** Description: Whether to enable the extra current for i-neurons

Parameter: ***xcG_i*** Description: The maximum conductance

Parameter: ***xcP_***i Description: The multiplicity of gating elements in the ionic channels

Parameter: ***xcQ***_i Description: The multiplicity of gating elements in the ionic channels

Parameter: ***xcVr***_i Description: The reversal potential

Parameter: ***xcVa***_i Description: The threshold of the steady state curve in the a ODE

Parameter: ***xcSa***_i Description: The slope of the steady state curve in the a ODE

Parameter: ***xcTa***_i Description: The time constant in the a ODE

Parameter: ***xcVb***_i Description: The threshold of the steady state curve in the b ODE

Parameter: ***xcSb***_i Description: The slope of the steady state curve in the b ODE

Parameter: ***xcTb***_i Description: The time constant in the b ODE

## Panel: Model (MOD-Cur)

Arachne supports importing custom currents from MOD files and embedding them into the modelled neurons. On this panel, user can select the files and assign those parameters of the currents which are usually assigned in HOC code. The set of MOD currents can be different for neurons of e-type and i-type, but it is the same within all neurons of one type. By default, there is no MOD currents imported into the program. If there is more than 1 MOD file selected or more than 1 current defined in a file, then all the currents are summed up.

User can tune the program to watch values of MOD currents in the selected neurons making settings on “Measured (X/MOD-Cur)” and “Plot (X/MOD-Cur)” panels. If any neurons are selected for watching, the curves of the currents versus time will be shown after the simulation completes or the progress snapshot is taken for the running simulation with “SCRIPT_TakeSnapshot”.

After user imports any currents with GUI, the MOD files are translated into C++ code automatically to provide maximum performance of computation for the currents. The new code is embedded into HPC kernel source code which is recompiled automatically afterwards. As a result, starting simulation with new MOD currents takes longer than usually. But if user uses the same set of MOD currents for the second time (including the same parameters assigned for each current), the translation and compilation steps are skipped.

Currently Arachne does not provide full-fledged support of MOD files ignoring “UNITS”, “KINETIC” and some other blocks.


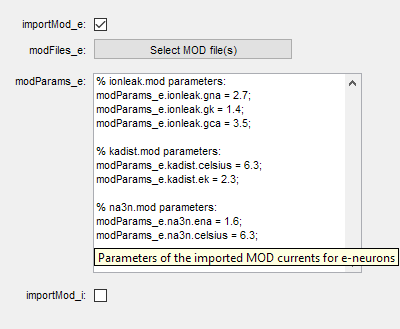
 Parameter: ***importMod_e*** Description: Whether to import MOD files with extra currents for e-neurons

Parameter: ***modFiles_e*** Description: Selector of MOD files with extra currents for e-neurons

Parameter: ***modParams_e*** Description: Parameters of the imported currents for e-neurons

Parameter: ***importMod_i*** Description: Whether to import MOD files with extra currents for i-neurons

Parameter: ***modFiles_i*** Description: Selector of MOD files with extra currents for i-neurons

Parameter: ***modParams_i*** Description: Parameters of the imported currents for i-neurons

## Panel: HPC

Parameter: ***fakeMPI*** Description: Whether to use the fake MPI version of gamma sumulator

Parameter: ***scalTest***. Description: On/off scalability test unchecked: run HPC kernel once for number of processes equal np and number of threads equal nt checked: run HPC kernel sequentially for number of processes equal minNP , minNP + 1, ..., maxNP and number of threads equal 1, 2, ..., maxNT

Parameter: ***np*** Description: Number of MPI processes. The parameter specifies the number of parallel processes to launch.

When you’re changing it and going to run simulation on the cluster, make sure that “np” does not exceed the number of available cluster nodes. The number of available nodes is specified in the files “hostfile_BusyMaster” and “hostfile_IdleMaster” living in the directory “/home/reviewer/gs/worker/hostfiles”. The first file corresponds to the case when the master node works the same as a slave; the second file is for the case when the master node is not loaded. In the case of scalability test (see “scalTest” parameter below) “np” specifies the maximum number of processes in the test.


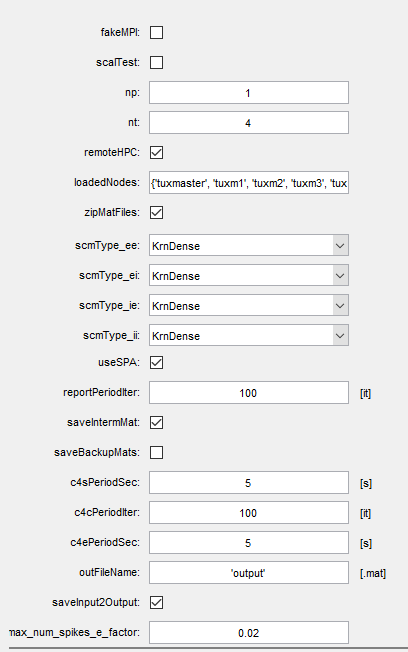
 Parameter ***idleMaster***. The parameter specifies whether the master node of the cluster idles. true — all slave nodes do a job, the master node idles. false — all slave nodes and the master node do a job.

Parameter: ***nt*** Description: Number of OMP threads per MPI process. The parameter specifies the number of threads per process. While one process is run on one processor, one thread is run on one core of the processor. The best performance is achieved when the number of processes equals number of cluster nodes (i.e. the total number of processors) and the number of threads per process equals number of processor cores. In the case of scalability test “nt” specifies the maximum number of threads per processes in the test.

Parameter: ***minNP*** Description: Minimum number of MPI processes. The parameter specifies the minimum number of processes (cluster nodes) used in scalability test. (The maximum number equals np.) Usage of this parameter is worth when

a) memory deficit does not allow running scalability test for small number of loaded cluster nodes;

b) testing for small number of processes is expected to be too long or does not provide an interest.

Parameter: ***maxNP*** Description: Maximum number of MPI processes

Parameter: ***maxNT*** Description: Maximum number of OMP threads per MPI process

Parameter: ***remoteHPC*** Description: Whether to use remote High Performance Computing server unchecked: call HPC kernel on this machine/cluster checked: call HPC kernel on remote machine/cluster

Parameter: l***oadedNodes*** Description: List of nodes to run simulation on

Parameter: ***zipMatFiles*** Description: Whether input/output MAT-files should be zipped before and unzipped after transferring between local machine and head node of the cluster. unchecked: the files are transferred without compressing checked: the files are compressed before and decompressed after transferring

Parameter: ***scmType_ee*** Description: Type of synaptic conductance matrix. These parameters specify the types of synaptic conductance matrices. The type of a matrix determines how and where the matrix will be created and populated, and what algorithm will be used for computation of the current on actual iteration

**AllZeros**: The matrix is not generated explicitly. All elements are zeros

**AllEqual**: The matrix is not generated explicitly. All elements are equal The matrix is not generated explicitly because it has primitive structure. The main advantages of these types are:

a) matrix can have giant size not requiring a lot of memory;

b) calculation of the current on actual iteration has excellent performance.

But it’s clear that these models of synaptic conductance matrix are too simple and make sense only in combination with other types.

Notice that these two types of matrices can be used only when useHC = false

**HstDense**: The matrix is generated in Matlab and saved to input MAT-file with other data. The matrix is generated in dense form

**HstSparse**: The matrix is generated in Matlab and saved to input MAT-file with other data. The matrix is generated in sparse form. The matrix is generated in MATLAB host program, saved to the input MAT-file with other data, uploaded to the head node of the cluster and scattered among all nodes. The difference between dense and sparse storage scheme is that only nonzero elements positions and values are stored in the second case. It provides memory economy in the case if the number of nonzero elements is much less than the number of zeroes.

The main advantage of these two types is that the matrices can be populated in MATLAB in variety of ways. There is no restriction on number of different values in a matrix. There is no need to recompile HPC kernel if algorithm of matrix population is changed.

The disadvantages are as follows:

a) amount of physical memory on local machine and head node of cluster puts limitation on matrix size;

b) uploading big matrices to the head node may take a lot of time;

c) scattering matrices from the head node to all nodes takes time as well.

Notice that type “HstSparse” can be used only when sclModel = BSD and useHC = false

**KrnDense**: The matrix is generated in HPC kernel before 1st iteration already in distributed form. An element of the matrix is stored in floating-point-number format

**KrnSparse**: The matrix is generated in HPC kernel before 1st iteration already in distributed form.

The matrix is generated in sparse form the matrix is generated in distributed sparse form in HPC kernel.

The matrix is generated in HPC kernel before 1st iteration already in distributed form.

These types are counterparts of types HstDense and HstSparse that provide the advantage that there is no need to upload and scatter big matrices. Since matrices are generated already in distributed form, the total amount of memory required by a matrix is divided by number of cluster nodes. As a result, bigger matrices can be generated in comparison with types HstDense and HstSparse. The disadvantage is that there is no such flexible way to populate synaptic conductance matrices. If any changes in the algorithm, HPC kernel should be recompiled.

Notice that type “KrnSparse” can be used only when sclModel = BSD and useHC = false

**KrnOneBit**: The matrix is generated in HPC kernel before 1st iteration already in distributed form.

An element of the matrix is stored in one bit An element of the matrix is stored in one bit.

The matrix has dense structure and is generated in HPC kernel before 1st iteration already in distributed form.

The advantage of this mode compared with “KrnDense” type is that the matrix stored in bit-packed format requires significantly less memory than the matrix stored in the floating-point format. The matrix of type “KrnOneBit” can be populated with only two different values: zero value and some other value.

The feature of type “KrnOneBit” in comparison with other ones is that it requires rounding of number of rows in the matrix: the number of rows has to be evenly divisible by 64. If this is not the case, the rounding is performed automatically to the nearest integer that fits the requirement. As a result, the number of neurons used in simulation becomes less.

Notice that type “KrnOneBit” can be used only when sclModel = BSD and useHC = false

**KrnInPlace**: The matrix is re-generated in HPC kernel on each iterations.

Parameter: ***scmType_ei*** Description: Type of synaptic conductance matrix. Each element is generated each time it’s used.

The matrix is re-generated on each iteration once again. The random number generator that produces the sequence of elements of the matrix is reseed with the same seed before each computation of the matrix-to-vector product. This type of synaptic conductance matrix provides vanishing limitations of the model size, but the performance of this type is the worst among all other types. The reason is that the call of random number generator for each element takes considerable time in total.

Notice that type “KrnInPlace” can be used only when useHC = false

**AllZeros**: The matrix is not generated explicitly. All elements are zeros

**AllEqual**: The matrix is not generated explicitly. All elements are equal

**HstDense**: The matrix is generated in Matlab and saved to input MAT-file with other data. The matrix is generated in dense form

**HstSparse**: The matrix is generated in Matlab and saved to input MAT-file with other data. The matrix is generated in sparse form

**KrnDense**: The matrix is generated in HPC kernel before 1st iteration already in distributed form. An element of the matrix is stored in floating-point-number format

**KrnSparse**: The matrix is generated in HPC kernel before 1st iteration already in distributed form.

The matrix is generated in sparse form

**KrnOneBit**: The matrix is generated in HPC kernel before 1st iteration already in distributed form.

An element of the matrix is stored in one bit

**KrnInPlace**: The matrix is re-generated in HPC kernel on each iteration

Parameter***: scmType_ie*** Description: Type of synaptic conductance matrix

**AllZeros**: The matrix is not generated explicitly. All elements are zeros

**AllEqual**: The matrix is not generated explicitly. All elements are equal

**HstDense**: The matrix is generated in Matlab and saved to input MAT-file with other data. The matrix is generated in dense form

**HstSparse**: The matrix is generated in Matlab and saved to input MAT-file with other data. The matrix is generated in sparse form

**KrnDense**: The matrix is generated in HPC kernel before 1st iteration already in distributed form. An element of the matrix is stored in floating-point-number format

**KrnSparse**: The matrix is generated in HPC kernel before 1st iteration already in distributed form. The matrix is generated in sparse form

**KrnOneBit**: The matrix is generated in HPC kernel before 1st iteration already in distributed form. An element of the matrix is stored in one bit

**KrnInPlace**: The matrix is re-generated in HPC kernel on each iteration

Parameter: scmType_ii

Description: Type of synaptic conductance matrix **AllZeros**: The matrix is not generated explicitly. All elements are zeros

**AllEqual**: The matrix is not generated explicitly. All elements are equal

**HstDense**: The matrix is generated in Matlab and saved to input MAT-file with other data. The matrix is generated in dense form

**HstSparse**: The matrix is generated in Matlab and saved to input MAT-file with other data. The matrix is generated in sparse form

**KrnDense**: The matrix is generated in HPC kernel before 1st iteration already in distributed form. An element of the matrix is stored in floating-point-number format

**KrnSparse**: The matrix is generated in HPC kernel before 1st iteration already in distributed form. The matrix is generated in sparse form

**KrnOneBit**: The matrix is generated in HPC kernel before 1st iteration already in distributed form. An element of the matrix is stored in one bit

**KrnInPlace**: The matrix is re-generated in HPC kernel on each iteration

Parameter: useSPA Description: Use Single Precision Arithmetics instead of double precision one. The parameter specifies whether to use Single Precision Arithmetics instead of double precision one.

true — to use float data type in all computations. (4-byte floating-point numbers provide approx. 7 decimal digits accuracy.)

false — to use double data type. (8-byte floating-point numbers provide approx. 16 decimal digits accuracy.)

In general, usage of floats instead of doubles leads to the better performance and worse accuracy.

But in the case of ARACHNE the worse accuracy is not relevant because the algorithm of simulation is so that the noise is introduced into the data on each iteration explicitly. Therefore, the difference between single-precision results and double-precision ones can be considered as a part of the intrinsic noise.

Parameter: distMatPVH Description: In what mode matrices of presynaptic voltage history will be presented unchecked: local mode checked: distributed mode The parameter specifies in which mode matrices of presynaptic voltage history are held.

true — matrices are held in distributed mode (each node of cluster keeps only part of each matrix).

false — matrices are held in local mode (each node of cluster keeps each matrix in whole).

If this option is enabled, amount of required physical memory per cluster node decreases, but the time of simulation session considerably increases due to communication between processes.

Use distMatPVH = true only if there isn’t enough memory for local mode of presynaptic voltage history matrices. In this case the number of processes has to be as little as it is possible.

Parameter: ***reportPeriodIter*** Description: How often HPC kernel should report its progress? The report will be done once per iterations. The parameter specifies how often HPC kernel should report its progress (i.e. the number of current iteration and the duration of an iteration). The report is done once per “reportPeriodIter” iterations.

Parameter: ***saveIntermMat*** Description: Whether to save intermediate data file "intermediate.mat that makes it possible to stop simulation and continue afterwards from the same point. The file is saved when: 1) specified t_final is reached; 2) termination is requested by user; 3) saveBackupMats == true and current iteration number is evenly divisible by backupPeriodIter . Notice that for avoidance of unpractical usage of physical memory this parameter can be equal “true” only when distMatPVH = false.

Parameter: ***saveBackupMats*** Description: Whether backup files should be saved periodically. Saving backup files makes it possible to recover simulation progress in the case of abnormal termination. If saveIntermMat == false, then only "output.mat" is saved, if saveIntermMat == true, then both "output.mat" and "intermediate.mat" are saved periodically.

Parameter: ***backupPeriodIter*** Description: How often backup files should be saved? The saving will be done once per ***backupPeriodIter*** iterations.

Parameter: ***backgroundMode*** Description: Whether to run HPC kernel in background mode. The parameter specifies whether to run HPC kernel in background mode.

true — HPC kernel is run in background mode (MATLAB can be idle).

false — HPC kernel is run in foreground mode (MATLAB is busy).

The key difference between foreground and background modes is that the second mode does not require persistent connection between MATLAB host program and HPC kernel at time of the whole simulation session. In the first mode HPC kernel dumps information about current progress into console and MATLAB host shows this output to user as is. In the second case HPC kernel dumps information about current progress into status file from time to time and MATLAB host picks it up periodically, analyses and shows current progress to user.

Parameter: ***c4sPeriodSec***. Description: How often Matlab host should check the status of HPC kernel? (The status check means that the host program determines if HPC kernel is running and if so, what the current iteration number is.) The check is done once per c4ePeriodSec seconds. (c4e = Check For Execution.)

Parameter: ***PeriodIter*** Description: How often HPC kernel should check for a command from Matlab host? (There are two commands: terminate, dump snapshot.) The check will be done once per iterations.

Parameter: ***c4ePeriodSec*** Description: How often Matlab host should check if HPC kernel has executed requested command? (There are two commands: terminate, dump snapshot.) The check will be done once per seconds.

Parameter: ***outFileName*** Description: Output MAT-file name

Parameter: ***saveInput2Output*** Description: Whether to save input parameters to output MAT-file

Parameter: max_num_spikes_e_factor Description: The limit for number of spikes per one simulation session. It will be used to preallocate the following arrays in HPC kernel: idx_e_spikes, t_e_spikes and other. The limit for number of spikes of a type is computed in HPC kernel as: max_num_spikes_e = int32( num_e * m_steps * max_num_spikes_e_factor );

Parameter: max_num_spikes_i_factor Description: The limit for number of spikes per one simulation session. It will be used to preallocate the following arrays in HPC kernel: idx_i_spikes, t_i_spikes and other. The limit for number of spikes of a type is computed in HPC kernel as: max_num_spikes_i = int32( num_i * m_steps * max_num_spikes_i_factor );

## Panel: RNG (Random Number Generator)


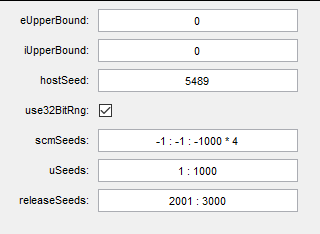
 Parameter: ***eUpperBound*** Description: Maximum value of u_e

Parameter: ***iUpperBound*** Description: Maximum value of u_i

Parameter: ***hostSeed*** Description: Seed for Matlab random number generator

Parameter: ***use32BitRng*** Description: Random Number Generator to use unchecked: fine-grained 64-bit Random Number Generator checked: coarse-grained 32-bit Random Number Generator. The parameter specifies whether to use coarse-grained 32-bit random number generator or fine-grained 64-bit one.

true — to use coarse-grained 32-bit random number generator.

false — to use fine-grained 64-bit random number generator.

The 32-bit generator (std::mt19937) provides higher performance and worse quality of random numbers, while the 64-bit generator (std::mt19937_64) provides lower performance and better quality of the numbers. Usage of the 64-bit generator makes sense only with double precision arithmetics (useSPA = false).

Parameter: ***scmSeeds*** Description: Random number generator seeds to generate matrices in HPC kernel

Parameter: uSeeds Description: Random number generator seeds to generate vectors u_e and u_i on each iteration

Parameter: releaseSeeds Description: Random number generator seeds to support release probabilities (i.e. space-time-random connections between neurons)

## Panel: Kinetic (Kinetic of the basic currents)

The biophysics description of kinetics constant of voltage gated current in i- and e-neurons with 4 basic kinetics constant ***h, m, n***: Example of ***h*** kinetics for e-neurons

With basic voltage equation of e-neurons

Parameter: h_i_v_1 Description: Defines the current in neurons

Parameter: m_i_v_1 Description: Defines the current in neurons

Parameter: n_i_v_1 Description: Defines the current in neurons

Parameter: h_e_v_1 Description: Defines the current in neurons

Parameter: m_e_v_1 Description: Defines the current in neurons

Parameter: n_e_v_1 Description: Defines the current in neurons

Parameter: h_i_v_2 Description: Defines the current in neurons

Parameter: m_i_v_2 Description: Defines the current in neurons

Parameter: n_i_v_2 Description: Defines the current in neurons

Parameter: h_e_v_2 Description: Defines the current in neurons

Parameter: m_e_v_2 Description: Defines the current in neurons

Parameter: n_e_v_2 Description: Defines the current in neurons

Parameter: h_i_a_1 Description: Defines the current in neurons

Parameter: m_i_a_1 Description: Defines the current in neurons

Parameter: n_i_a_1 Description: Defines the current in neurons

Parameter: h_e_a_1 Description: Defines the current in neurons

Parameter: m_e_a_1 Description: Defines the current in neurons

Parameter: n_e_a_1 Description: Defines the current in neurons

Parameter: h_i_a_2 Description: Defines the current in neurons

Parameter: m_i_a_2 Description: Defines the current in neurons

Parameter: n_i_a_2 Description: Defines the current in neurons

Parameter: h_e_a_2 Description: Defines the current in neurons

Parameter: m_e_a_2 Description: Defines the current in neurons

Parameter: n_e_a_2 Description: Defines the current in neurons

Parameter: h_i_b_1 Description: Defines the current in neurons

Parameter: m_i_b_1 Description: Defines the current in neurons

Parameter: n_i_b_1 Description: Defines the current in neurons

Parameter: h_e_b_1 Description: Defines the current in neurons

Parameter: m_e_b_1 Description: Defines the current in neurons

Parameter: n_e_b_1 Description: Defines the current in neurons

Parameter: h_i_b_2 Description: Defines the current in neurons

Parameter: m_i_b_2 Description: Defines the current in neurons

Parameter: n_i_b_2 Description: Defines the current in neurons

Parameter: h_e_b_2 Description: Defines the current in neurons

Parameter: m_e_b_2 Description: Defines the current in neurons

Parameter: n_e_b_2 Description: Defines the current in neurons

Parameter: phi Description: Defines the current in neurons

## Panel: Initial Conditions

***Initial condition for dynamics variances***

Parameter: v_e Description: Voltage of e-neuron at time 0.

Parameter: n_e Description: Kinetics constant

Parameter: m_e Description: Kinetics constant

Parameter: h_e Description: Kinetics constant

Parameter: s_e Description: Kinetics constant

Parameter: v_i Description: Voltage of i-neurons

Parameter: n_i Description: Kinetics constant

Parameter: m_i Description: Kinetics constant

Parameter: h_i Description: Kinetics constant

Parameter: s_i Description: Kinetics constant

Parameter: s_stoch_e Description: Parameter of synaptic activation of e-neurons

Parameter: s_stoch_i Description: Parameter of synaptic activation of inter neurons

## Panel: Init. Cond. (Astro)

Parameter: Ca Description: Calcium concentration in astrocyte

Parameter: ip3 Description: Concentration of IP3

Parameter: q Description: Kinetic parameter of Ca current

Parameter: y Description: Rate of release of IP3

## Panel: Init. Cond. (X-Cur)

Parameter: xcA_e Description: Vector of activation variables for e-neurons

Parameter: xcB_e Description: Vector of inactivation variables for e-neurons

Parameter: xcA_i Description: Vector of activation variables for i-neurons

Parameter: xcB_i Description: Vector of inactivation variables for i-neurons

## Panel: Measured

Parameter: opRadii Description: Radii of the observation points in polar coordinate system

Parameter: opAngles Description: Angles of the observation points in polar coordinate system

Parameter: electrolCond Description: The electolytic conductance

Parameter: stabAnalysis Description: Stabilization analysis mode unchecked: compute Frequency_e, Frequency_i, syncoef_e, syncoef_i just once at the end of the simulation; checked: compute Frequency_e, Frequency_i, syncoef_e, syncoef_i on each iteration (this affects performance and does not follow the original Matlab code). On/off stabilization analysis mode.

true — to compute “Frequency_e”, “Frequency_i”, “syncoef_e”, “syncoef_i” on each iteration and to show plots at the end of simulations.

false — to compute “Frequency_e”, “Frequency_i”, “syncoef_e”, “syncoef_i” just once at the end of simulations.

Notice that computation of the parameters on each iteration affects performance.

Parameter: gatherSCM Description: Show matrices of synaptic conductance at end of simulation

Parameter: watchGTonicGABA Description: Whether to collect data GTonicGABA vs time

Parameter: watchedCellIdx_e Description: Array of indexes of e-cells to watch presynaptic voltage of (the curves of voltage vs time will be shown)

Parameter: watchedCellIdx_i Description: Array of indexes of i-cells to watch presynaptic voltage of (the curves of voltage vs time will be shown)

Parameter: watchedSynIdx_ee Description: Array of indexes of ee-synapses to watch synaptic conductance for

Parameter: watchedSynIdx_ei Description: Array of indexes of ei-synapses to watch synaptic conductance for

Parameter: watchedSynIdx_ie Description: Array of indexes of ie-synapses to watch synaptic conductance for

Parameter: watchedSynIdx_ii Description: Array of indexes of ii-synapses to watch synaptic conductance for

Parameter: frequencyParam Description: Network frequency

Parameter: syncoefParam Description: Network synchronization

## Panel: Measured (Astro)

Parameter: watchedAstroIdx

Description: Array of indexes of watched cells that specifies: 1) astrocytes to watch Ca of (the curves of Ca vs time will be shown); 2) astrocytes to watch the activation parameter "y" of; 3) e-neurons to watch the release probability "e->i" of (the curves of releaseProb_ei vs time will be shown). The array can be empty.

Parameter: gatherCaColormap Description: Whether to gather data for Ca colormap

Parameter: caColormapPeriodIter Description: How often to get the Ca vector being a column of the colormap (in iterations)

## Panel: Measured (X/MOD-Cur)

Parameter: ***watchedExtraCurrentIdx***_e Description: Array of neurons indexes to watch extra currents in e-network.

Parameter: ***watchedExtraCurrentIdx***_i Description: Array of neurons indexes to watch extra currents in i-network.

Parameter: ***watchedModCurrentIdx***_e Description: Array of neurons indexes to watch MOD-currents in e-network.

Parameter: ***watchedModCurrentIdx***_i Description: Array of neurons indexes to watch MOD-currents in i-network.

## Panel: Plot

Parameter: ***plotRastr*** Description: Plot rastergram

Parameter: ***plotQR*** Description: Plot the quality of recall coefficient

Parameter: ***plotQRPeriod*** Description: Plot 1/Frequency_e computed from the beginning of the latest recall session

Parameter: ***plotSCM*** Description: Plot synaptic conductance matrices

Parameter: ***plotFrequency*** Description: Plot network frequencies

Parameter: ***plotSynCoef*** Description: Plot network synchronization coefficients

Parameter: ***winSizeDivisor*** Description: The parameter used when moving averages and moving sample standard deviations are computed. Size of the moving window is computed as size of the signal divided by winSizeDivisor. The lower value of the divisor, the stronger smoothing.

Parameter: ***stdDevFactor*** Description: The factor used when we plot range "moving average +- stdDevFactor * moving sample standard deviation"

Parameter: ***plotGTonicGABA*** Description: Plot GTonicGABA

Parameter: ***plotPresynVoltages*** Description: Plot presynaptic voltage vs time for selected neurons

Parameter: ***plotSynCondVsTime*** Description: Plot synaptic conductance vs time for selected synapses

Parameter: ***plotPotentials*** Description: Plot potentials in observation points

Parameter: ***plotSpectra*** Description: Plot power spectra of potentials in observation points

Parameter: ***numFreqFactor*** Description: Bin calculation of extracellular field (spectrum). The number of frequencies to show in the spectra: numFreq = numFreqFactor * numTicks where numFreq is the number of frequencies, numTicks is the number of time steps.

Parameter: ***subtractMean*** Description: Whether to subtract mean value from potentials to delete the impulse in spectra at zero frequency

Parameter: ***winType*** Description: Type of weight window used to preprocess the potentials in order to suppress side lobes of impulses in the spectra. Use '@rectwin' to turn off this kind of preprocessing. See the list of supported window types here: [***http://www.mathworks.com/help/signal/ref/window***](http://www.mathworks.com/help/signal/ref/window). If Signal Processing Toolbox is not installed, then rectangular window is used.

Parameter: ***winOpts*** Description: Parameters of the window (see for types info)

## Panel: Plot (Astro)

Parameter: plotCaStyle Description: Plot style for calcium curves:

- DoNotPlot: Do not plot any curve;
- PlotCurvesSeparately: Plot each curve separately (on individual figure);
- PlotCurvesTogether: Plot all curves together (on the same figure).

Parameter: plotActParamStyle Description: Plot style for activation parameter curves

Parameter: plotReleaseProbStyle Description: Plot style for release probability curves

Parameter: plotCaColormap Description: Whether to plot Ca colormap

## Panel: Plot (X/MOD-Cur)

Parameter: ***plotStyleExtraCurrent_e*** Description: Plot style for Extra current for e-neurons curves:

- DoNotPlot: Do not plot any curve;
- PlotCurvesSeparately: Plot each curve separately (on individual figure);
- PlotCurvesTogether: Plot all curves together (on the same figure).

Parameter: ***plotStyleExtraCurrent_i*** Description: Plot style for Extra current for i-neurons curves

Parameter: ***plotStyleModCurrent_e*** Description: Plot style for MOD current for e-neurons curves

Parameter: ***plotStyleModCurrent_i*** Description: Plot style for MOD current for i-neurons curves

## How to change parameters on the remote cluster.

1. Change or delete the parameters.
2. Log in to the master node and open the following C++ file:

“/home/reviewer/gs/worker/***.h”.

1. Update the code for the function “AtomicCorrection” as you need. Notice that in C++ the numbering of array elements begins from zero.
2. Compile HPC kernel running script “***build_Linux_RELEASE.sh***” living in the same directory.

If compilation is successful, the MATLAB host program being run for the next time will use the new just compiled HPC kernel.

## (Example) How to changing deterministic external drives to E- and I-cells

1. Log in to the master node and open the following file:

“/home/reviewer/gs/worker/ExternalDrives.cpp”.

1. Update the code for the functions “ComputeExternalDrive_e” and “ComputeExternalDrive_i” as you need.

You can follow the example provided in the file “ExternalDrives.cpp” for the case of “I_e” being

I_e(t, idx) = sin(PI **·** idx / (num_e – 1)) exp(–0.5 **·** t)

with “idx” being end-to-end index of the distributed vector: idx = 0, 1, …, num_e – 1.

1. Compile HPC kernel running script “build_Linux_RELEASE.sh” living in the same directory.

If compilation is successful, the MATLAB host program being run for the next time will use the new just compiled HPC kernel.

## (Example) Changing system of equations

The system of equations is defined by the following functions: “h_e_inf”, “h_i_inf”, “m_e_inf”, “m_i_inf”, “n_e_inf”, “n_i_inf”, “tau_h_e”, “tau_h_i”, “tau_n_e”, “tau_n_i”.

Each function is defined in a separate CPP file under the following directory:

“/home/reviewer/gs/worker”.

The exception is for functions “m_e_inf” and “m_i_inf”. They are in both C++ files (“m_e_inf.cpp” and “m_i_inf.cpp”) and MATLAB files (“m_e_inf.m” and “m_i_inf.m”). If any changes, make sure that the code is identical in corresponding files.

When you are changing any functions, follow these rules:

1. Make sure that a function does not encounter uncertainty for some specific values of input argument. For example, function “m_e_inf” encounters uncertainty 0 / 0 in the cases when input argument v equals –54 and –27. You must provide special handling of the cases because otherwise the function can return NaN (Not-A-Number) at some moment of simulation making all further process spoiled.
2. Avoid usage of types float or double explicitly, use template type T instead. Each function is compiled in two versions: T = float and T = double. The version used at time of simulation depends on value of variable “useSPA” specified in file “HpcParams.m”. Do not specify rational constants as 1.2 or 3.4 because they would have double type, specify them as T(1.2) and T(3.4) instead. However, integer constants can be specified without indication of type T.
3. Avoid division of integer numbers. For example, defining variable x as

T x = T(0.5) + 1 / 3; you will actually have x equal T(0.5). The reason is that quotient of integer numbers is rounded to integer. The correct forms are:

T x = T(0.5) + T(1) / 3;

T x = T(0.5) + 1 / T(3);

1. Do not forget to compile HPC kernel running script “build_Linux_RELEASE.sh” living in the same directory.

If you added some new functions, make sure that they are specified with “inline” keyword and listed in files “GammaSimulator.h” and “GammaSimulator.cpp”.

## (Example) Choosing and initialization of random number generators

Random number generators are used in three places.

1. The MATLAB host program uses one to prepare input data for the simulation.

The generator is initialized with the seed specified by variable “seed” in file “PrepareInputData.m”.

1. If “scmType_XY” is equal to “KrnDense”, “KrnSparse”, “KrnOneBit” or “KrnInPlace”, then the C++ worker program uses one generator per thread to initialize local chunks of distributed matrices “g_ee”, “g_ei”, “g_ie”, and “g_ii”. Each thread must have random number generator initialized with unique seed. If “scmType_XY” is equal to “KrnInPlace”, then the matrices are generated SAME on different iterations.

The seeds for all threads of all processes are collected into one-dimensional array “scmSeeds” defined in file “PrepareInputData.m”. This array is saved to input MAT-file with other data. Each value of a seed, including positive values and negative values, corresponds to unique state of random number generator.

The requirements for “scmSeeds” are as follows:

a) “scmSeeds” must be integer vector of length not less than np **·** nt **·** 4;

b) “scmSeeds” must contain values in range [-2147483648, 2147483647] except 0;

c) “scmSeeds” must not contain equal values;

d) “scmSeeds” must not overlap with “uSeeds” (see below).

1. The C++ part of the software uses one generator per thread to initialize the local chunks of distributed vectors “u_e” and “u_i” in the file “DoSimulation.cpp”. Each thread must have random number generator initialized with a unique seed. The DIFFERENT vectors are generated on different iterations.

The seeds for all threads of all processes are collected into one-dimensional array “uSeeds” defined in the file “START_Arachne.m.” This array is saved to the input MAT-file with other data. Each value of a seed, including positive values and negative values, corresponds to unique state of random number generator.

The requirements for “uSeeds” are as follows:

a) “uSeeds” must be integer vector of length not less than np **·** nt;

b) “uSeeds” must contain values in range [-2147483648, 2147483647] except 0;

c) “uSeeds” must not contain equal values;

d) if “scmType_XY” is equal to “KrnDense”, “KrnSparse”, “KrnOneBit” or “KrnInPlace”, then “uSeeds” must not overlap with “scmSeeds”.

It’s possible to choose quality of random number generators used in HPC kernel. Boolean variable “use32BitRng” defined in the file “HpcParams.m” switches between 32-bit and 64-bit random number generators.

32-bit generator std::mt19937 produces integer random numbers in the interval [0, 232). When these numbers are converted to floating-point ones and projected to the interval [0, 1), the step of the grid becomes equal approx. 2.3283 · 10–10. It's sufficient when useSPA = true because the step supported by the floats

eps(single(1)) = 1.1921 **·** 10–7.

But it's not sufficient when useSPA = false because the step supported by the doubles

eps(double(1)) = 2.2204 **·** 10–16.

64-bit generator std::mt19937_64 produces integer random numbers in the interval [0, 264). When these numbers are converted to floating-point ones and projected to the interval [0, 1), the step of the grid becomes equal approx. 5.4210 **·** 10–20. It’s sufficient for reaching the limit of granularity of double-precision arithmetic.

Changing Parameters in the case of simulation continuation

If the parameter “saveIntermMat” is specified as true, then HPC kernel saves all necessary data so that simulation can be continued after stopping. In that case you cannot change any parameters of simulation; it will continue with the same parameters independently on any changes made in files “HpcParams.m” and “ModelParams.m”. The only exception is for the following three parameters:

1. np — the number of processes;
2. nt — the number of threads per process;
3. idleMaster — the flag indicating whether the master node idles.

You can change these parameters, and this change will be applied in the next simulation session. The only restriction is that the product np **·** nt must be the same as before.

# Model restrictions, memory requirements and performance

## Bit synaptic conductance matrices

Synaptic conductance matrices have a bit structure if *scmType_XY = KrnOneBit*. They provide compromise between the cases “performance is low, but big models are allowed” (*scmType_XY = KrnInPlace*) and “performance is high, but big models are not allowed” (*scmType_XY = KrnDense*). The principal limitation of the 1-bit mode is that a synaptic conductance matrix can be populated with only two different values (zero and some other value).

In order to achieve the best performance, the number of rows in 1-bit element matrix should be evenly divisible by 64. If this is not the case, then the number of rows (and the number of neurons of a type) is rounded to the nearest less integer that is evenly divisible by the factor.

Let’s consider the following example:

num_e = 3000; num_i = 5000;

scmType_ee = KrnSparse; scmType_ei = KrnOneBit;

scmType_ie = KrnSparse; scmType_ii = KrnSparse.

The type “scmType_ei” requires rounding of number of neurons (since it is equal to “KrnOneBit”), while three other types do not require any rounding. The number of rows in matrix “g_ei”, i.e. “num_e”, should be evenly divisible by 64. As a result, the numbers of neurons will be rounded as follows:

num_e = 2944; num_i = 5000.

## Memory allocated on a cluster node

The memory requirements are checked by the function that lives in the file “host\OtherUtils\CheckMemReq.m”. If memory allocated by four synaptic conductance matrices and four matrices of presynaptic voltage history exceeds the threshold “memPerNodeLimit”, then the function prevents simulation launching to avoid the memory deficit on cluster nodes.

Each type of synaptic conductance matrix specified by “scmType_XY” parameter has specific memory requirements checked by the function that lives in the file “host\OtherUtils\CountMatrixMemReq.m”. The requirements for physical memory per cluster node (in MB) for synaptic conductance matrices are estimated as follows:

| **Synaptic conductance matrix type** | **Memory allocated for the matrix on a cluster node (in MB)** |
| --- | --- |
| AllZeros  AllEqual  KrnInPlace | **reqMemPerNode = 0**.  This is a rough estimate that takes into scope that the matrices are not stored in memory explicitly. |
| HstDense | **reqMemPerNode = numElem · elemSize · (np + 1) / np / 220**  with “numElem” being the number of elements in the matrix,  “elemSize” being the size of an element. The size is equal 4 if *useSPA = true* and 8 otherwise.  This estimate takes into scope that the most memory is allocated on the master node after loading of the matrix from MAT-file and scattering. |
| HstSparse | **reqMemPerNode = (f(intSize) + f(intSize / 2) / np) / 220**  with **f(size) = numElem · (elemSize + size) + (numCols + 1) · size**,  “numElem” being the rough estimate of the number of nonzero elements in the matrix which depends on the height and the standard deviation of the bell:  **numElem = round(w_max · (exp(-A2) - 1 + A · sqrt(π) · erf(A)) / B),**  **B = A2 / (numRows - 1) / (numCols - 1),**  **A = sqrt(0.5) · (max(numRows, numCols) - 1) / sigma**,  “elemSize” being equal to 8 (MATLAB does not support single-precision sparse arrays),  “intSize” being equal to 8 (MATLAB does not support 4-bit integer sparse arrays).  This estimate takes into scope that the most memory is allocated on the master node after loading of the matrix from MAT-file and scattering. |
| KrnDense | if *useHC = true* and *saveIntermMat = true* or *showSCM = true*  **reqMemPerNode = numElem · elemSize · (np + 1) / np / 220**  else  **reqMemPerNode = numElem · elemSize / np / 220**.  The estimate is similar to that one for “HstDense”, but if we don’t need to gather the matrix on the master node for writing to MAT-file this estimate is (np + 1) times lower. |
| KrnSparse | **reqMemPerNode = (numElem · (elemSize + intSize) + (numCols + 1) · intSize) / np / 220**.  with “numElem” being similar to that one for “HstSparse”,  “intSize” being equal 4,  “elemSize” being the size of an element. The size is equal 4 if *useSPA = true* and 8 otherwise. |
| KrnOneBit | **reqMemPerNode = numElem / blockSize · intSize / np / 220**  with “numElem” being the number of elements in the matrix,  “blockSize” being 64,  “intSize” being 8. |

When scalability test is run, the worst value for number of processes “minNP” is substituted instead of “np”.

Notice that mode “KrnOneBit” requires the number of rows in the matrix to be evenly divisible by the 64. If this is not the case, rounding to the nearest lower integer value that fits the requirement is performed. As a result, allocated memory becomes lower (see [previous paragraph](#_1-bit_synaptic_conductance)).

The requirements for physical memory per cluster node (in MB) for presynaptic voltage history matrices depend on value of variable “distMatPVH” (see the paragraph [The file "HpcParams.m"](file:///C:\Users\lsavtchenko\AppData\Roaming\Skype\My%20Skype%20Received%20Files\HpcParams.m#_The_file_)) and are estimated as follows:

| **distMatPVH** | **Memory allocated for the matrix on a cluster node (in MB)** |
| --- | --- |
| false | **reqMemPerNode = numElem · elemSize / 220**  with “numElem” being the number of elements in the matrix,  “elemSize” being the size of an element. The size is equal 4 if *useSPA = true* and 8 otherwise. |
| true | **reqMemPerNode = numElem · elemSize / np / 220**  The estimate is similar to that one for *distMatPVH = false*, but np times lower |

Notice that if *saveIntermMat = true*, we have to gather presynaptic voltage history matrices for writing them to intermediate MAT-file, i.e. the memory for whole matrix has to be allocated on master node. Therefore this value of variable “saceIntermMat” is forbidden when *distMatPVH = true* to avoid unpractical usage of memory.

## Limitations of the number of neurons caused by memory requirements

Let’s consider the following example:

num_e = 2 · num_i; radius_e = 250; radius_i = 200; v = 0.1; sclModel = BSD; useHC = false;

w_ee_max = 2; w_ii_max = 0.8; w_ei_max = 0.9; w_ie_max = 0.3;

sigma_ee = 100; sigma_ei = 75; sigma_ie = 75; sigma_ii = 50;

np = 9; useSPA = true; saveIntermMat = false; memPerNodeLimit = 1700.

The maximum number of neurons with which simulation can be launched is estimated as follows:

| **scmType_XY** | **Maximum number of neurons** | |
| --- | --- | --- |
|  | **distMatPVH = true** | **distMatPVH = false** |
| KrnDense | num_e = 29000; num_i = 14500 | num_e = 8800; num_i = 4400 |
| KrnSparse | num_e = 39600; num_i = 19800 | num_e = 8800; num_i = 4400 |
| KrnOneBit | num_e = 39400; num_i = 19700 | num_e = 8800; num_i = 4400 |
| KrnInPlace | num_e = 40000; num_i = 20000 | num_e = 9000; num_i = 4500 |

## Performance of ARACHNE

The main parameters that determine ARACHNE performance are the following:

1. the number of processes “np”;
2. the number of threads “nt”;
3. the numbers of neurons “num_e” and “num_i”;
4. the parameters of ring networks “radius_e”, “radius_i” and “v”;
5. the types of synaptic conductance matrices “scmType_XY” with “XY” being “ee”, “ei”, “ie”, “ii”;
6. the mode of presynaptic voltage history matrices “distMatPVH”;
7. the variable “useHC” which specifies whether to correct synaptic conductance matrices according to Hebbian theory;
8. the maximum heights of corresponding bells “w_XY_max” (for sparse matrix only);
9. the standard deviations of corresponding normal distributions “sigma_XY” (for sparse matrix only).

In general case, the most time-consuming operation performed on each iteration is computation of the actual current. This operation has quadratic complexity while other operations have linear complexity. The current is computed 8 times on each iteration. For each matrix of synaptic conductance (“g_ee”, “g_ei”, “g_ie”, “g_ii”) the operation is provided twice.

Let’s consider complexity of current computation for a matrix “g_XY” of size “num_X” by “num_Y”. Denote *N1 = num_X + num_Y* — the sum of height and width of the matrix, *N2 = num_X · num_Y* — the number of elements in the matrix, *N3* — the number of nonzero elements in sparse matrix which depends on “num_X”, “num_Y”, “w_XY_max” and “sigma_XY”, *n = np · nt* — the total number of workers. Then complexity of current computation for the matrix “g_XY” is estimated as follows:

| **Synaptic conductance matrix type** | **Complexity** |
| --- | --- |
| AllZeros | O(num_Y / n) |
| AllEqual | O(N1 / 2 / n) |
| HstDense  KrnDense  KrnOneBit  KrnInPlace | O(N2 / 2 / n) |
| HstSparse  KrnSparse | if *distMatPVH = false*  O(N3 / n)  if *distMatPVH = true*  O(N3 · num_X / 2 / n) |

That means that doubling of the number of workers and preserving all other parameters unchanged brings halving of calculation time. The statement is true with the following remarks:

1. When ARACHNE is running with *distMatPVH = true*, this statement is true only if number of neurons and processes is rather little. The cause of this effect is that communication between processes on each iteration becomes huge when the number of neurons is rather big. Usually with little number of neurons and processes the mode *distMatPVH = false* with better performance can be used. If there is a memory deficit and ARACHNE can be running only with *distMatPVH = true*, the number of processes must be as little as it is possible.
2. When ARACHNE is running with *np > 1*, there is the overhead due to communication between processes. As a result, performance for *np = 2* becomes approximately twice better than performance for *np = 1* only when the number of neurons is large enough to compensate the overhead.
3. When ARACHNE is running with *nt > 1*, the overhead due to communication between threads is much less than overhead due to communication between processes. As a result, performance for *nt = 2* becomes approximately twice better than performance for *nt = 1* for much less number of neurons, than it was necessary in the point 1.
4. The “O” symbol used in the table denotes linear dependence on the argument, but does not state the slope coefficient. The slope depends on synaptic conductance matrix type. For example, the slope for “KrnInPlace” matrix type is higher that the slope for “KrnDense” matrix type. (The product is computed slower for the first type and faster for the second type.) At the same time, the slope coefficients are equal for the following types of synaptic conductance matrices:
5. “HstDense” and “KrnDense”;
6. “HstSparse” and “KrnSparse”.

## Scalability curves for different types of matrices

Let’s consider the following example:

num_e = 8700; num_i = 4350; radius_e = 250; radius_i = 200; v = 0.1;

sclModel = BSD; useHC = false; t_final = 0.2;

w_ee_max = 2; w_ii_max = 0.8; w_ei_max = 0.9; w_ie_max = 0.3;

sigma_ee = 1000; sigma_ei = 750; sigma_ie = 750; sigma_ii = 500;

scalTest = true; np = 9; nt = 4; minNP = 8; useSPA = true; use32BitRng = true;

memPerNodeLimit = 1700; stabAnalysis = false; saveIntermMat = false; saveBackupMats = false.

When *distMatPVH = false* the simulation scales, but the scalability is far away from the ideal. The cause of this effect is memory deficit which make impossible to consider enough neurons to show real scalability.

| 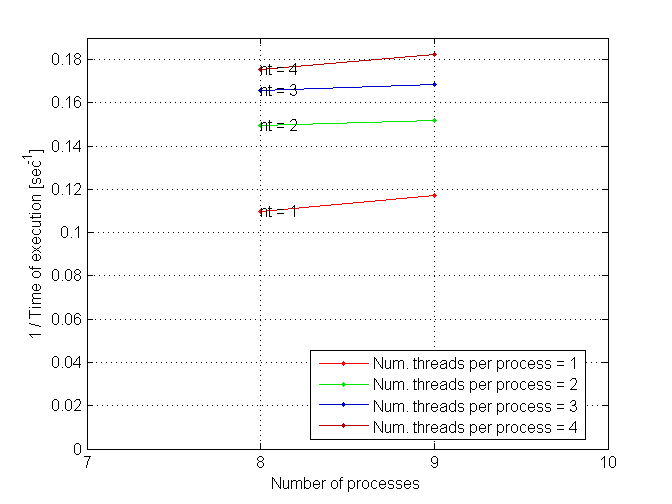 | 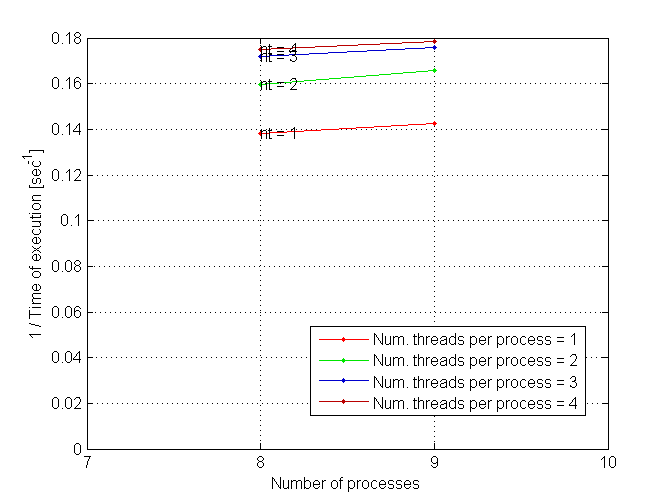 |
| --- | --- |
| Picture 4.1. *scmType_XY = KrnDense* | Picture 4.2. *scmType_XY = KrnSparse* |
| 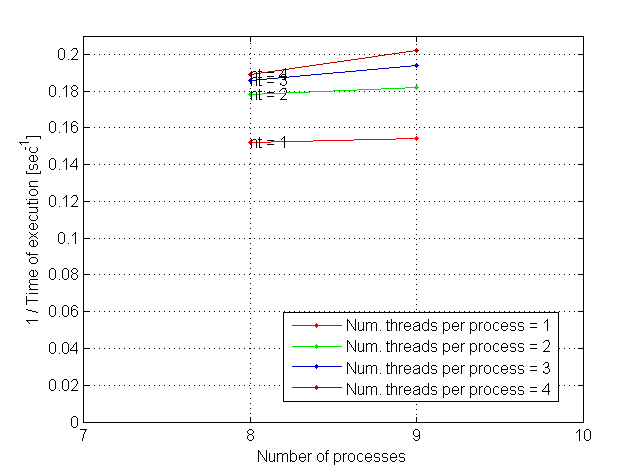 | 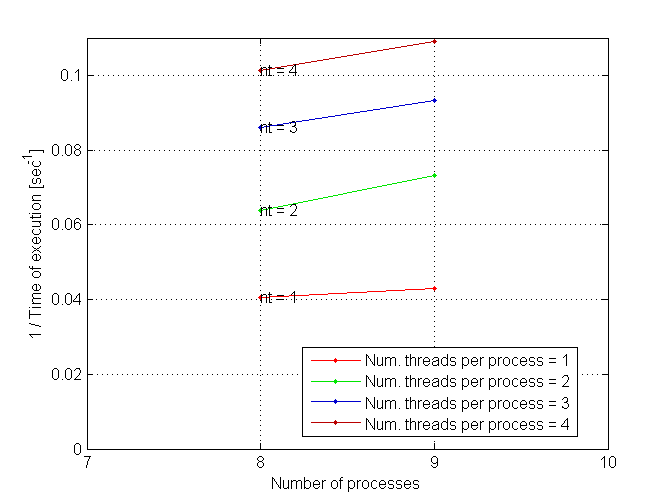 |
| Picture 4.3. *scmType_XY = KrnOneBit* | Picture 4.4. *scmType_XY = KrnInPlace* |

When *distMatPVH = true* there is no scalability in most cases because of active communication between processes. The simulation scales only with little number of neurons and processes, i. e. when communication between processes isn’t very sizeable, but in this case the option *distMatPVH = false* is better to use.

# Structure of MATLAB host program

The main file of the host program is “**START_Arachne.m”**. It’s used for

1. starting of simulation from scratch;
2. attaching to and monitoring of simulation running in background at the moment;
3. continuing previous simulation session or continuing simulation after a crash and a recovery;
4. running scalability test;
5. grabbing results of previous simulation session and showing them to user.

The next three scripts are used for servicing of background simulation mode:

1. **“SCRIPT_GetSnapshot.m”** — this script does the request to HPC kernel to dump current results of simulation. After the dumping, the results file “output.mat” is taken by the host program (downloaded if *remoteHPC = true* or copied otherwise) and analysed. Then the results are shown to user graphically. When HPC kernel creates a snapshot, it also creates a backup point that can be used for progress recovery.
2. **“SCRIPT_TerminateBackgroundProcess.m”** — this script does the request to HPC kernel to save current results and terminate.
3. **‘SCRIPT_KillBackgroundProcess.m”** — this scripts terminates all HPC kernel processes forcibly. Current progress is lost, but it’s possible to recover some old progress from backup file(s) if the variable “saveBackupMats” was equal “true”.

Notice that the scripts 1 and 2 described above execute asynchronous operations between host and kernel, therefore, require some time. They are controlled by variables c4cPeriodIter and c2ePeriodIter defined in the file “HpcParams.m”.

The next two scripts are used for other servicing of ARACHNE:

1. **“SCRIPT_RecoverBackupProgress.m”** — this script recovers progress of simulation from the latest backup file(s). It’s worth in the case if simulation was stopped unexpectedly for some reason not depending on the program (e.g. impact of hardware, network, other software etc.). In order to make ARACHNE save backup file(s) periodically, specify *saveBackupMats = true* in the file “HpcParams.m”. Both files “output.mat” and “intermediate.mat” are subjects of recovery, but the latter file can be recovered only if simulator was set up to save the file (i.e. *saveIntermMat = true*). There are two backup storages used by HPC kernel in turn and backup storage pointer file that points to the storage that will be used for saving next time. Such approach makes it possible to recover even in the case if crash happens at the moment of backup file(s) saving.
2. **“SCRIPT_CleanUp.m”** — this script cleans up HPC kernel I/O directories (see [the paragraph](#_I/O_directories) below). After running of the script, it’s impossible to get results of the latest simulation, continue or recover progress. All the data are deleted. Simulation can be run only from scratch.

The script **“UTILITY_GetMaxModelSize.m”** is used to get the maximum size of model which meets memory requirements. It calls “ModelParams.m” and “HpcParams.m” to initialize all required parameters except of “num_e” and “num_i”, asks user to enter the ratio “num_i / num_e” and computes the maximum number of neurons for adjusted model and HPC parameters.

# Structure of HPC kernel

## Source code

| **File** | Description |
| --- | --- |
| main.cpp | The file contains ARACHNE HPC kernel entry point function “main”. It reads variables “useSPA” and “use32BitRng” from input MAT-file and calls templated function “main_templated” with appropriate type arguments. The templated function calls three modules to read all other stuff of the MAT-file, run the simulation itself and save results to output MAT-file(s). |
| MatFileIOUtils.h  MatFileIOUtils.cpp | Utility functions to read scalars, vectors and matrices from input MAT-file and broadcast or scatter them among processes.  Utility functions to save scalars, vectors and matrices to output MAT-files. |
| GammaSimulator.h  GammaSimulator.cpp | Templated class containing all data and functions comprising internal logic of33 simulation. |
| ReadAllocateWrite.cpp | The modules of ARACHNE responsible for reading of all variables from input MAT-file, allocating memory for them and other internal objects, writing of simulation results to output MAT-files. |
| DoSimulation.cpp | The time evolution cycle module. |
| UpdateIdxTNumSpikes.cpp | Selection of cells spiked at current time step. Update of the arrays that track spiked cells. |
| h_e_inf.cpp  h_i_inf.cpp  m_e_inf.cpp  m_i_inf.cpp  n_e_inf.cpp  n_i_inf.cpp  tau_h_e.cpp  tau_h_i.cpp  tau_n_e.cpp  tau_n_i.cpp | The functions defining system of equations. In the most they migrated from the original software without changes. The only new feature is the control of uncertainties added to avoid spoiling of simulation in the case if a function encounters division 0 / 0. |
| ExternalDrives.cpp | The functions defining external drives to E- and I-cells (migrated from file “params.m”). |
| LocalVector.h  LocalVector.cpp | Templated class representing local vector being analogue of one-dimensional dense array in MATLAB. |
| DistVector.h  DistVector.cpp | Templated class representing distributed vector, i.e. the vector with data evenly divided among processes. |
| ScmType.h | Enumerable that specifies the type of synaptic conductance matrix. |
| LocalMatrix.h  LocalMatrix.cpp | Templated class representing local dense matrix being analogue of two-dimensional dense array in MATLAB. |
| LocalSparseMatrix.h  LocalSparseMatrix.cpp | Templated class representing local sparse matrix being analogue of two-dimensional sparse array in MATLAB. The difference is that MATLAB does not support single-precision sparse arrays, while this class does support. |
| DistMatrixBase.h | Templated abstract base class for all distributed matrix classes: “ZeroDistMatrix”, “ConstDistMatrix”, “DistMatrix”, “DistSparseMatrix”, “OneBitDistMatrix” and “InPlaceDistMatrix”. The class represents a matrix with columns evenly divided among all processes (i.e. each process contains all the rows, but only some number of columns). All the classes derived from this class must implement the method that computes the current on actual iteration. |
| ZeroDistMatrix.h  ZeroDistMatrix.cpp | Templated class representing distributed matrix with all elements equal zero. The class is used if *scmType_XY = AllZeros*. |
| ConstDistMatrix.h  ConstDistMatrix.cpp | Templated class representing distributed matrix with all equal elements. The value of all elements equal to is stored in an object of the class. The class is used if *scmType_XY = AllEqual*. |
| DistMatrix.h  DistMatrix.cpp | Templated class representing distributed dense matrix with all elements stored in floating-point format. The class is used if *scmType_XY = HstDense* or *scmType_XY = KrnDense*. |
| DistSparseMatrix.h  DistSparseMatrix.cpp | Templated class representing distributed sparse matrix with all elements stored in floating-point format. The class is used if *scmType_XY = HstSparse* or *scmType_XY = KrnSparse*. |
| OneBitDistMatrix.h  OneBitDistMatrix.cpp | Templated class representing distributed dense matrix populated with only two different values. Each matrix element is stored in one bit of memory. Unset bit corresponds to zero matrix element, set bit corresponds to some value. (The value is a scalar contained in the class object.) The class is used if *scmType_XY = KrnOneBit*. |
| InPlaceDistMatrix.h  InPlaceDistMatrix.cpp | Templated class representing distributed dense matrix generated in place. The matrix allocates just a little amount of physical memory; it’s reconstructed as a sequence of elements many times at time of simulation. The class object contains random number generator seeds to make each local chunk of the matrix re-generated same. The class is used if *scmType_XY = KrnInPlace*. |
| DistMatrixFactory.h  DistMatrixFactory.cpp | Factory function that creates distributed synaptic conductance matrices with parameters read from the file "input.mat". If “scmType_XY” is equal to “KrnDense”, “KrnSparse”, “KrnOneBit” or “KrnInPlace”, then each matrix is initialized with other set of seeds for random number generators. |
| ElementwiseOp1.cpp  ElementwiseOp2.cpp  ElementwiseOp3.cpp  ElementwiseOp4.cpp | Element-wise operations being part of mathematical core of ARACHNE. Each operation has many input vectors and many output vectors. The vectors are processed in element-wise manner by different processes/threads independently. |
| ComputeSynCoef.cpp | Method that computes synchronization coefficient between neuron spikes. |
| DistEnv.h  DistEnv.cpp | Distributed environment variable and utility function staff. |
| FixCurrentProgress.cpp | Method that prints overall progress and refreshes status file in background simulation mode.  Method that saves backup files. |
| OtherFileIOUtils.h  OtherFileIOUtils.cpp | I/O file utility functions that are not related to reading from and writing to MAT-files. They provide support of background simulation mode and backup saving of the progress. |
| DistributionWrapper.h  DistributionWrapper.cpp | Wrapper of the random number generator facade class std::uniform_real_distribution<T>. The wrapper itself plays minor technical role making it possible to use the generator in particular software. |
| GetTypeTagUtils.h | Templated utility functions that return MATLAB data type tag and MPI data type tag given template argument. |
| MathUtils.h  MathUtils.cpp | Various mathematical functions used in tests. |

Notice that functions “m_e_inf” and “m_i_inf” live not only in C++, but also in MATLAB files “m_e_inf.m” and “m_i_inf.m”. If any changes, make sure that the code is identical.

## I/O directories

All files HPC kernel works with live in the directory “worker/iofiles.” The directory contains some subdirectories listed below:

1. “iofiles/host-kernel” — this directory is used to store all files MATLAB host passes to HPC kernel. The files are:
2. “input.mat” — input data for simulation,
3. “terminate” — empty file signalling about termination request,
4. “snapshot” — empty file signalling about snapshot request.
5. “iofiles/kernel-host” — this directory is used to store all files HPC kernel passes to MATLAB host. The files are:
6. “output.mat” — the file with results of simulation,
7. empty file with name pattern “iter %i - %i; %g sec” or “iter %i (%i - %i); %g sec” used to report current progress of HPC kernel running in background mode.
8. “iofiles/kernel-host/snapshot”— this directory is used to store the snapshot file “output.mat” dumped by HPC kernel in background mode per user’s request.
9. “iofiles/kernel-kernel” — this directory is used to store the file “intermediate.mat” saved by HPC kernel and loaded afterwards to continue previous simulation session (the files “input.mat” and “output.mat” are read in that case as well).
10. “iofiles/backup” — this directory is used to store backup data. There are two backup storage directories: “iofiles/backup/backup-1” and “iofiles/backup/backup-2” used by HPC kernel in turn. Each backup storage directory contains two subdirectories: “kernel-host” for the file “output.mat” and “kernel-kernel” for the file “intermediate.mat.” There is empty pointer file in the directory “iofiles/backup” that points to the backup storage directory that will be used for the next backup saving. The name of the file “1” or “2” indicates the storage.

# Passing new parameters from host (MATLAB) to workers (C++)

## Passing a new scalar to an equation

Let’s consider the case when you need to make some equation dependent on some new floating-point scalar variable defined in MATLAB.

For example, you want to make function “tau_n_e” defined in file “tau_n_e.cpp” dependent on variable “myScalar” defined in MATLAB code.

1. Open file “PrepareInputData.m” and find “if useSPA” statement. Add your variable to the body of the statement as it was done for other variables.
2. Find “input = ...” statement and add the name “myScalar” to the list using single quotes (notice that MATLAB syntax uses single quotes ′ instead of double quotes ″ to denote string literals).
3. Log in to the master node and open the file

“/home/reviewer/gs/worker/GammaSimulator.h”.

Find the place where the following variables “dt”, “dt05”, “v_rev_e”, … are declared and add the declaration of new variable “myScalar” in the same way.

1. Open file “/home/reviewer/gs/worker/ReadAllocateWrite.cpp” and find the place where the variables “dt”, “v_rev_e”, “v_rev_i”, … are read from MAT-file.

Add one more line for the new variable as follows:

myScalar = ReadCheckBroadcastScalar<T>("myScalar").

1. Open file “/home/reviewer/gs/worker/tau_n_e.cpp” and utilize the new variable as you need. For example, you can replace line

T beta_n = T(0.5) **·** exp(–(v + 57) / 40);

with the following line:

T beta_n = myScalar **·** exp(–(v + 57) / 40).

1. Run script “/home/reviewer/gs/worker/build_Linux_RELEASE.sh” to recompile the program.

## Passing a new vector to an element-wise operation

Let’s consider the case when you need to make some element-wise operation dependent on some new floating-point vector defined in MATLAB.

### The case of scattering of the vector

The scattering means that the vector read from input MAT-file on the master process is divided into parts so that each process receives only one part of the vector.

Let’s suppose that you want to make function “ElementwiseOperation1” defined in file “ElementwiseOp1.cpp” dependent on vector “myVector” of length “num_e” defined in MATLAB code.

1. Open file “PrepareInputData.m” and find “if useSPA” statement. Add your vector to the body of the statement as it was done for other variables.
2. Find “input = ...” statement and add the name “myVector” to the list using single quotes.
3. Log in to master node and open the file

“/home/reviewer/gs/worker/GammaSimulator.h”.

Find the place where the following vectors “v_e”, “n_e”, “m_e”, … are declared and add the declaration of new vector “myVector” in the same way.

1. Open file “/home/reviewer/gs/worker/ReadAllocateWrite.cpp” and find the place where the vectors “v_e”, “n_e”, “m_e”, … are read from MAT-file.

Add one more line for the new vector as follows:

myVector = ReadCheckScatterVector<T>("myVector", num_e);

1. Open file “/home/reviewer/gs/worker/ElementwiseOp1.cpp” and utilize the new vector as you need. Use “myVector[idx]” to read an element of the vector in the cycle.
2. Run script “/home/reviewer/gs/worker/build_Linux_RELEASE.sh” to recompile the program.

### The case of broadcasting of the vector

The broadcasting means that the vector read from input MAT-file on master process is cloned among all processes. In general, passing a vector in such way from MATLAB to HPC kernel is similar to the case of scattering of the vector described above. The only differences are as follows:

1. The vector should be read form MAT-file with help of other method:

myVector = ReadCheckBroadcastVector<T>("myVector", num_e);

1. Access to elements of broadcasted vectors in element-wise operations should not be done by the same indices as for scattered vectors. The reason is as follows. Length of local portion of scattered vector depends on number of processes while local length of broadcasted vector does not depend on number of processes. As a result, access by “myVector[idx]” may cause out-of-range error if “idx” is the same index variable used to access scattered vectors of length “num_e”.

## Biophysics model

### Model equations for membrane potential dynamics

Dynamics of membrane potential *V* of both types of neurons is described by a set of equations with the Hodgkin–Huxley formalism:

The computational kernel located on a remote cluster calculates the set of Eq. , where *INa* and *IK* are the sodium and potassium currents respectively, *Iapp* is a current used to simulate the dynamics of external excitation, *IL* is a leaking current, *Isyn* is a sum of synaptic currents. The *INa* and *IK* are simulated using a previously reported approach. The С++ code for calculating the Eq. with the kinetics for i-neurons and e-neurons was adopted from the NEURON database (https://senselab.med.yale.edu; ModelDB accession no. 138421).

The ARACHNE also provides the option to add mod-files generated by NEURON software tool.

### Model equations for synaptic currents

Chemical synapses have varied conductances, release probabilities and spatial distributions. The synaptic current in the model is defined as *I = gnsn*(*V-Vnr*)*,* where *n* = 1,...,4 denotes one of the four synaptic types - *ee, ii, ei,* or *ie*, respectively, *Vnr* is a revers potential*, gn* and *sn* are the synaptic conductance and kinetics defined as:

where

,

The variable *v* is dimensionless.

Eq. describes synapses from *e*-neurons to *Rj-*neurons *(*where *Rj* is either *ej-cell* or *ij* -cell depending on the postsynaptic cell), while Eq. describes synapses from *i*-neurons to *Rj–*neurons*.* The total synaptic current received by a single neuron is dependent on the network size and the synaptic distributions.

### Network organization: Topology

ARACHNE basic network connectivity is represented by circle. This (a) enables full cell inter-connectivity, without edge effects or specific boundary conditions, (b) makes all cell locations a priori equivalent, and (c) can be described by a single size parameter, radius *R*.

The sizes of the ***i***- and ***e***-neurons networks can vary independently. In contrast, the ***a*** and ***e*** networks have similar sizes and are positioned 'next' to each other. The latter reflects the fact that, throughout area CA1 of the hippocampus, pyramidal cells are arranged in a regular layer and surrounded by a relatively uniform scatter of astrocytes. Accordingly, modelled astrocytes are distributed evenly across the pyramidal cell network, with a fixed total cell number (in our case study it is equal to the number of e-neurons, for simplicity). Such an arrangement is to represent diffuse (volume-transmitted) astrocyte-neuron signalling without loss of generality.

### Network organization: Synaptic strengths and their distributions.

The two neuronal networks, ***i*** and ***e***, have four types of synaptic connections: ***ee, ei, ie, ii*** described. For each type, ***ee, ei, ie, ii,*** the model provides several key parameters, such as synaptic strength ***w***, synaptic current rise time ***r*** and decay time ***d*** , and probability of neurotransmitter release *p*. All connection strength values between *i*th and *j*th neurons, at any given time, comprise the classical connectivity matrix. One distinct and novel feature of the model is that the value of *p* can be modified by the activity of nearby astrocytes, in accord with the current view in the field of glia-neuron signalling. Synaptic strength ***w*** can also be modified by the three mechanisms of synaptic plasticity incorporated to the model, described in the sections below.

In the brain, the synaptic strength ***w*** appears to depend on the distance between cortical neurons: to recapitulate this observation the model provides two complementary types of connectivity. The first type, termed bell-shaped strength (*BSS*) model, incorporates a Gaussian distribution of ***w*** (centred at the 'presynaptic' cell) with the uniform connection density between the nearest 50% of all network neurons and standard deviation . The second type, a bell-shaped density (*BSD*) model, incorporates uniform distribution of ***w*** buta Gaussian distribution of cell-cell connection density, with the number of connections decreasing with distance from the 'presynaptic' cell.

In addition to having individual synaptic currents, the model enables tonic excitation or inhibition currents, a rapidly emerging feature of hippocampal networks.

It is important to note that the user can easily change the type of astroglia and neurons, synapses and their distribution type, and other type of plasticity other biophysical parameters, using only a GUI without changing the core program on the server.

### Release probability distribution

In the basic model, hetero- and homogeneous spatial distributions of the release probability *p* have been incorporated. The heterogeneous *p* pattern which is typical for excitatory synapses on CA1 e-neurons could be applied to the synapses make connections between the networks *ei* and *ie*. The ability to vary the spatial distribution of *p* is important for exploring the space-delimited influence of astrocytes on synaptic transmission.

The value of ***p*** can range between zero and one, which could be set manually or can be made dependent on the calcium activity of astrocytes.

### Setting the external input

In the model, which has no hierarchical structure (for the sake of generality), external (sensory) input can be mimicked by a distributed excitatory stimulation of e-cells. The network-wise distribution of such stimuli is given by the input's external pattern *(EP)* with the adjustable current amplitude. For small networks, the *EP* can be set using the method of a dynamic matrix, with matrix elements representing activated or non-activated neurons. For large networks, when the matrix size is large, the *EP* can be defined using a binary drawing with black pixels depicting activated e-neurons. The total size of the *EP* in pixels, or the size of dynamic matrix, thus represents the total number of ***e***-neurons.

In the standard network memory paradigm, brain circuits memorize a particular *EP* through the activity-induced changes in synaptic weights, which forms a new connectivity (synaptic weight) matrix.

#### All-inclusive model of synaptic modifications

Classically, experimental studies documented activity-dependent alterations in synaptic weighting as an individual event statistically validated within a specific time window. In most cases, the temporal dynamics of synaptic efficacy change are either neglected or unattainable through reliable measurements. However, the characteristic time interval over which synaptic modifications continue to change ("remembered") is a key parameter in memorizing the external input to the neural network. The present model enables the setting of a dynamic time-dependent modification of synaptic weights with the following integral-differential equation:

where the time bin *T* is an user defined interval of a correlation between times of presynaptic and postsynaptic APs.

The correlation function between APs is , where . The function provides selection of spike pairs so that the integral becomes valuable only in the vicinities of points (*τ*1, *τ*2) means that the integral has significant value only when *τ*1 and *τ*2 are within certain intervals depending on *k*.

The discrete version of Eq. is

, . The sum is taken for all pairs of spikes in the interval (*t*k-1*­* ‒ *T*, *t*k-1)

To quickly adapt experimental STDP curves to the model networks, the software provides a utility that calculates and visualizes the STPD curve base on inputted parameters, against the experimental data.

### The network recall

The recall is a process associated with the network's response to the repeated *EP*. The response is a pattern of spiking (active) and non-spiking (passive) neurons. The quality of recall, ***C,*** is defined by the correlation between recalled and 'perfect' responses to the *EP*:

where *n* is total number of neurons of the network, *m* is a the maximum number of neurons in the active state of ideal response, *x* and *y* denote the state of any neuron of ideal and real responses correspondently.

After simplification of Eq., the *C* of a network with a single stored image is:

where ***l*** and ***k*** are numbers of active “correctly” and “incorrectly” activated neurons in the recalled response, respectively; ***m*** is number of active neurons in the ideal response. The condition of ***m*** = 0 in Eq. implies total darkness for networks, i.e. an input image array of nothing.

### Basic set of parameters

Number of i-neurons is 100 and e-neurons is 200.

Radius of i-networks is 200 μm and e-networks is 250 μm.

Rate of signal propagation between neurons is 0.1 mm/ms.

Basic release probability of all synapses was set a pbasic = 0.2, pmax = 0.5 is maximum release probability which can reach the synapse at any calcium concentration, and [Ca]ss = 100 nM is a resting calcium concentration of astrocyte.

Standard deviation of synaptic distribution are ee= 10 neurons, ei= 12.5 neurons, ie= 8 neurons, ii= 11 neurons.

The single synaptic conductance of the basic model are *gie*=0.23 S, *gei*=0.29 S, *gii*=0.64 S, *gee*=4 nS.

Rise times are e=0.1 ms and i=0.3 ms, decay times are de=3 ms and di=9 ms.

The basic values of the parameters of tonic current are *Af* = 0. 01 S/cm2*, tp* = 0.02 ms‑1, *Gb* =0.1 μM, *fb* = 0.1 Hz, *dt*=0.02 μs, *T*= 100 ms.

The parameters of astrocyte dynamics used in the basic model are adapted from [21]

The parameters of link between astrocyte and neurons are *τ*spike= 2 mM/ms and *tp*= 0.02 ms-1.

These were the basic values used in the model and can be easily altered using GUI by the user.

### Equations for extracellular electric field

Electrical dynamics of brain networks is mainly registered using a field electrode located in the extracellular space. The electrode senses an extracellular current density generated by a population of neurons scattered around. Because field recordings are an essential method for neuroscientists, the present model includes a mechanism to calculate the extracellular field at a given radial coordinates with respect to the center of the network.

For simplicity, a model of the extracellular field generated by a single neuron is simulated as a monopole electrical current source between the neuron and the ground:

, where *I*(*r*) is density of transmembrane currents of the neurons ***e*,**,and of the neurons ***i***, *r* is the distance between the neuron and electrode, and *σ* is conductance of extracellular medium.

The electrical field generated by the network is

Model allows calculating the electric field for any single network as well as a superposition of both networks.
